# Association of Multiple-trait Polygenic Risk Score with Obesity and Cardiometabolic Diseases in Korean Population

**DOI:** 10.1093/gpbjnl/qzaf102

**Published:** 2025-11-09

**Authors:** Jinyeon Jo, Nayoung Ha, Yunmi Ji, Ahra Do, Je Hyun Seo, Bumjo Oh, Sungkyoung Choi, Eun Kyung Choe, Woojoo Lee, Jang Won Son, Sungho Won

**Affiliations:** Department of Public Health Sciences, Graduate School of Public Health, Seoul National University, Seoul 08826, Republic of Korea; Department of Public Health Sciences, Graduate School of Public Health, Seoul National University, Seoul 08826, Republic of Korea; Interdisciplinary Program in Bioinformatics, College of Natural Sciences, Seoul National University, Seoul 08826, Republic of Korea; Department of Public Health Sciences, Graduate School of Public Health, Seoul National University, Seoul 08826, Republic of Korea; RexSoft Corp., Seoul 08826, Republic of Korea; Veterans Medical Research Institute, Veterans Health Service Medical Center, Seoul 05368, Republic of Korea; Department of Family Medicine, Seoul Metropolitan Government, Seoul National University Boramae Medical Center, Seoul 07061, Republic of Korea; Department of Family Medicine, Seoul National University College of Medicine, Seoul 03080, Republic of Korea; Department of Mathematical Data Science, Hanyang University (ERICA), Ansan 15588, Republic of Korea; Division of Colorectal Surgery, Department of Surgery, Seoul National University College of Medicine, Seoul 03080, Republic of Korea; Healthcare Research Institute, Seoul National University Hospital Healthcare System Gangnam Center, Seoul 06236, Republic of Korea; Department of Public Health Sciences, Graduate School of Public Health, Seoul National University, Seoul 08826, Republic of Korea; Institute of Health and Environment, Seoul National University, Seoul 08826, Republic of Korea; Division of Endocrinology and Metabolism, Department of Internal Medicine, Bucheon St. Mary’s Hospital, College of Medicine, The Catholic University of Korea, Seoul 06591, Republic of Korea; Department of Public Health Sciences, Graduate School of Public Health, Seoul National University, Seoul 08826, Republic of Korea; RexSoft Corp., Seoul 08826, Republic of Korea; Institute of Health and Environment, Seoul National University, Seoul 08826, Republic of Korea

**Keywords:** Obesity, Cardiometabolic disease, Genome-wide association study, Polygenic risk score, Multiple-trait polygenic risk score

## Abstract

We conducted a comprehensive genetic investigation of obesity in a cohort of 93,673 Korean individuals, categorized by body mass index and waist circumference using Korean-specific and international criteria. To explore the genetic architecture of obesity and its related comorbidities, we performed genome-wide association studies and constructed polygenic risk scores (PRSs) using both conventional single-trait and advanced multiple-trait models, including the PRSsum approach. Our analyses identified genome-wide significant loci and demonstrated their higher heritability for general obesity than for abdominal obesity, and for moderate obesity than for severe obesity. East Asian populations showed stronger genetic correlations between abdominal obesity and obesity-related diseases. Both single-trait and multiple-trait PRSs stratified individuals by risk, with low-PRS individuals exhibiting reduced risk for obesity, hypertension, and type 2 diabetes, while high-PRS individuals displayed elevated risk, particularly under the multiple-trait model. Interaction and mediation analyses revealed distinct genetic pathways through which obesity contributes to disease development. Collectively, our findings revealed key loci and shared genetic mechanisms linking obesity and its comorbidities in the Korean population. These insights highlight the value of multiple-trait PRS models and underscore the importance of ancestry-specific genetic research for addressing the obesity epidemic.

## Graphical Abstract

**Figure:**
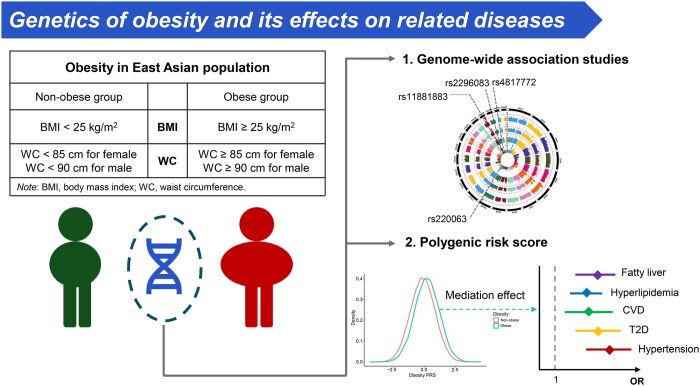

## Introduction

Obesity remains a major global health challenge, and its prevalence has steadily increased over the past few decades. According to the World Obesity Atlas 2023, approximately 38% of the global population is classified as overweight or obese [1]. This growing trend has substantially increased the risk of various chronic conditions, including type 2 diabetes (T2D), cardiovascular disease (CVD), hypertension (HT), dyslipidemia, and certain cancers, thereby exacerbating the global healthcare burden [2–8].

The clinical presentation and health impact of obesity vary considerably across ethnic groups. In particular, East Asian (EAS) populations tend to develop obesity-related cardiometabolic disorders at lower body mass index (BMI) thresholds than individuals of European ancestry [9,10]. Recognizing these population-specific differences, the Asia-Pacific World Health Organization (WHO) and the Korean Society for the Study of Obesity have proposed lower BMI cutoffs for defining obesity in EAS populations [11–14]. Moreover, EAS individuals often exhibit discordant obesity metrics, such as low BMI but relatively higher waist circumference (WC) [15], along with a higher prevalence of T2D [16]. These unique patterns underscore the need for a population-specific approach to obesity research, in contrast to studies focused on non-Hispanic White (NHW) populations of European ancestry.

Although genome-wide association studies (GWASs) have identified numerous single nucleotide polymorphisms (SNPs) associated with obesity-related indicators [17–23], most of these studies have concentrated on NHW populations and have primarily focused on BMI [24–28]. Consequently, their applicability in EAS populations remains limited [29–32]. In addition, existing generic risk prediction models, typically based on single-trait polygenic risk scores (PRSs), often fail to fully capture the complex interrelated genetic structures connecting obesity with related disorders.

In this study, we aimed to address these gaps by investigating shared genetic components underlying obesity and obesity-related diseases, including cardiometabolic disorders, in a large-scale Korean cohort. Unlike previous Korean research that focused on genetic heterogeneity among obesity subtypes stratified by metabolic health status [33], our research applied advanced methodologies, namely multiple-trait PRS models, including the integrative PRSsum approach. This framework enables the identification of shared genetic contributors to obesity and its comorbidities while improving the accuracy of risk prediction. By leveraging multiple-trait PRS strategies tailored to the EAS population, our findings offer insights for developing precise, population-specific preventive strategies to combat the growing obesity epidemic.

## Results

### Demographic characteristics of research participants

Our discovery dataset comprised 85,947 Korean individuals (51,317 females and 34,630 males). **Table 1** and **Table 2** summarize the demographic characteristics of the dataset, including the proportion of individuals with obesity. General and abdominal obesity were assessed using BMI- and WC-based criteria, respectively, as described in the Materials and methods section. Specifically, BMI-based classifications included BMI25 (≥ 25 kg/m^2^), BMI30 (≥ 30 kg/m^2^), and BMI40 (≥ 40 kg/m^2^), while WC-based classifications included WC1 (≥ 85 cm for females and ≥ 90 cm for males) and WC2 (≥ 95 cm for females and ≥ 100 cm for males). As shown in Table 1, 33.29% of participants were classified as obese and 2.96% as severely obese based on BMI. Similarly, Table 2 shows that 25.49% exhibited abdominal obesity, with 3.68% classified as severely obese based on WC.

**Table 1:** Baseline characteristics according to the incident general obesity in the discovery dataset

Variable	Total (*n* = 84,762)	BMI25 (prevalence = 33.29%)			BMI30 (prevalence = 2.96%)			Missing data *n* (%)
		Non-obese (*n* = 56,543)	Obese (*n* = 28,219)	*P* value	Non-obese (*n* = 82,251)	Obese (*n* = 2511)	*P* value	
Age (year): mean ± SD	52.77 ± 9.35	52.24 ± 9.39	53.85 ± 9.17	< 0.001	52.78 ± 9.34	52.51 ± 9.63	0.17	0 (0%)
Sex: *n* (%)				< 0.001			< 0.001	0 (0%)
Female	50,625 (59.73%)	36,064 (71.24%)	14,561 (28.76%)		49,065 (96.92%)	1560 (3.08%)		
Male	34,137 (40.27%)	20,479 (59.99%)	13,658 (40.01%)		33,186 (97.21%)	951 (2.79%)		
BMI (kg/m^2^): mean ± SD	23.92 ± 2.95	22.31 ± 1.80	27.16 ± 1.98	< 0.001	23.68 ± 2.63	31.81 ± 1.91	< 0.001	0 (0%)
WC (cm): mean ± SD				< 0.001			< 0.001	6834 (8.06%)
Female	78.76 ± 8.33	75.50 ± 6.47	86.67 ± 6.91		78.20 ± 7.75	96.08 ± 6.95		
Male	85.77± 7.50	82.03 ± 5.94	91.42 ± 5.91		85.36 ± 7.08	101.01 ± 6.65		
Body fat ratio (%): mean ± SD	25.56 ± 6.26	23.98 ± 5.74	28.83 ± 6.01	< 0.001	25.27 ± 6.03	35.28 ± 5.71	< 0.001	72,832 (85.93%)
Abdominal fat ratio (%): mean ± SD	0.88 ± 0.05	0.86 ± 0.04	0.93 ± 0.04	< 0.001	0.88 ± 0.05	0.99 ± 0.04	< 0.001	72,832 (85.93%)
SBP (mm Hg): mean ± SD	121.88 ± 15.31	119.53 ±14.91	126.57 ± 15.02	< 0.001	121.60 ± 15.23	130.92 ± 15.34	< 0.001	6328 (7.47%)
DBP (mm Hg): mean ± SD	76.21 ± 10.12	74.70 ± 9.85	79.23 ± 9.98	< 0.001	76.05 ± 10.08	81.50 ±10.23	< 0.001	6328 (7.47%)
FPG (mg/dl): mean ± SD	95.32 ± 19.99	93.39 ±18.50	99.16 ± 22.19	< 0.001	95.07 ± 19.73	103.48 ± 25.83	< 0.001	7831 (9.24%)
HbA1c (%): mean ± SD	5.72 ± 0.73	5.64 ±0.66	5.89 ± 0.84	< 0.001	5.71 ± 0.72	6.08 ± 0.95	< 0.001	39,833 (46.99%)
TG (mg/dl): mean ± SD	127.65 ± 87.13	114.89 ±77.74	153.11 ± 98.53	< 0.001	126.44 ± 86.24	167.31 ± 105.12	< 0.001	6401 (7.55%)
HDL (mg/dl): mean ± SD				< 0.001			< 0.001	6353 (7.50%)
Female	54.97 ± 13.32	56.69 ± 13.53	50.81 ± 11.78		55.15 ± 13.34	49.40 ± 11.34		
Male	48.26 ± 11.68	50.06± 12.16	45.57 ± 10.35		48.38 ± 11.71	43.92 ± 9.53		
HT: case *n* (%)	16,163 (22.42%)	8059 (17.02%)	8104 (32.73%)	< 0.001	15,196 (21.75%)	967 (43.42%)	< 0.001	12,661 (14.94%)
T2D: case *n* (%)	5845 (7.67%)	2832 (5.55%)	3013 (11.96%)	< 0.001	5434 (7.34%)	411 (18.59%)	< 0.001	8523 (10.06%)
CVD: case *n* (%)	3291 (4.64%)	1789 (3.83%)	1502 (6.20%)	< 0.001	3133 (4.55%)	158 (7.29%)	< 0.001	13,801 (16.28%)
HL: case *n* (%)	8339 (11.65%)	4813 (10.20%)	3526 (14.44%)	< 0.001	7970 (11.48%)	369 (17.01%)	< 0.001	13,153 (15.52%)
FL: case *n* (%)	3524 (5.78%)	1600 (3.90%)	1924 (9.67%)	< 0.001	3288 (5.55%)	236 (13.73%)	< 0.001	23,792 (28.07%)

*Note*: BMI25, BMI ≥ 25 kg/m²; BMI30, BMI ≥ 30 kg/m². BMI, body mass index; SBP, systolic blood pressure; DBP, diastolic blood pressure; FPG, fasting plasma glucose; TG, triglyceride; HDL, high-density lipoprotein; HT, hypertension; T2D, type 2 diabetes; CVD, cardiovascular disease; HL, hyperlipidemia; FL, fatty liver; SD, standard deviation.

**Table 2:** Baseline characteristics according to the incident abdominal obesity in the discovery dataset

Variable	Total (*n* = 79,113)	WC1 (prevalence = 25.49%)			WC2 (prevalence = 3.68%)			Missing data *n* (%)
		Non-obese (*n* = 58,946)	Obese (*n* = 20,167)	*P* value	Non-obese (*n* = 76,202)	Obese(*n* = 2911)	*P* value	
Age (year): mean ± SD	53.33 ± 8.82	52.61 ± 8.75	55.45 ± 8.66	< 0.001	53.24 (8.79)	55.68 (9.21)	< 0.001	0 (0%)
Sex: *n* (%)				< 0.001			< 0.001	0 (0%)
Female	49,005 (59.71%)	37,623 (76.77%)	11,382 (23.23%)		47,102 (96.12%)	1903 (3.88%)		
Male	30,108 (39.98%)	21,323 (70.82%)	8785 (29.18%)		29,100 (96.65%)	1008 (3.35%)		
BMI (kg/m^2^): mean ± SD	23.92 ± 2.94	22.90 ± 2.28	26.95 ± 2.58	< 0.001	23.70 ± 2.69	30.02 ± 2.87	< 0.001	1185 (1.38%)
WC (cm): mean ± SD				< 0.001			< 0.001	0 (0%)
Female	78.84 ± 8.37	75.38 ± 5.68	90.29 ± 4.91		78.03 ± 7.42	99.00 ± 4.12		
Male	85.74 ± 7.51	82.15 ± 5.28	94.48 ± 4.24		85.14 ± 6.84	103.31 ± 3.90		
Body fat ratio (%): mean ± SD	25.56 ± 6.26	24.23 ± 5.83	29.43 ± 5.85	< 0.001	25.21 ± 6.01	34.25 ± 5.87	< 0.001	67,188 (84.93%)
Abdominal fat ratio (%): mean ± SD	0.88 ± 0.05	0.87 ± 0.04	0.93 ± 0.04	< 0.001	0.88 ± 0.05	0.97 ± 0.04	< 0.001	67,188 (84.93%)
SBP (mm Hg): mean ± SD	121.88 ± 15.39	120.15 ± 15.04	126.92 ± 15.28	< 0.001	121.57 ± 15.28	129.87 ± 15.88	< 0.001	26 (0.03%)
DBP (mm Hg): mean ± SD	76.32 ± 10.18	75.25 ± 9.99	79.43 ± 10.09	< 0.001	76.14 ± 10.13	80.98 ± 10.37	< 0.001	26 (0.03%)
FPG (mg/dl): mean ± SD	94.96 ± 19.61	93.31 ± 17.99	99.76 ± 23.02	< 0.001	94.64 ± 19.20	103.35 ± 26.80	< 0.001	1585 (2.00%)
HbA1c (%): mean ± SD	5.71 ± 0.72	5.64 ± 0.65	5.91 ±0.86	< 0.001	5.69 ± 0.70	6.08 ± 0.99	< 0.001	3495 (42.34%)
TG (mg/dl): mean ± SD	128.12 ± 87.77	118.73 ± 81.59	155.53 ± 98.74	< 0.001	126.62 ± 86.58	167.34 ± 107.42	< 0.001	104 (0.13%)
HDL (mg/dl): mean ± SD				< 0.001			< 0.001	5 (0.01%)
Female	54.83 ± 13.32	56.24 ± 13.48	50.16 ± 11.65		55.08 ± 13.34	58.56 ± 11.32		
Male	48.19 ± 11.67	49.44 ± 12.01	45.32 ± 10.19		48.37 ± 11.69	44.36 ± 10.16		
HT: case *n* (%)	15,972 (21.96%)	9419 (17.47%)	6553 (34.85)	< 0.001	14,732 (21.05%)	1240 (45.26%)	< 0.001	6388 (8.07%)
T2D: case *n* (%)	5506 (7.29%)	3137 (5.45%)	2469 (12.76%)	< 0.001	5092 (6.86%)	514 (18.64%)	< 0.001	2180 (2.76%)
CVD: case *n* (%)	3076 (4.30%)	1885 (3.54%)	1191 (6.52%)	< 0.001	2876 (4.17%)	200 (7.58%)	< 0.001	7527 (9.51%)
HL: case *n* (%)	8361 (11.50%)	5549 (10.27%)	2812 (15.03%)	< 0.001	7888 (11.27%)	473 (17.43%)	< 0.001	6394 (8.08%)
FL: case *n* (%)	3524 (5.78%)	2073 (4.48%)	1451 (9.92%)	< 0.001	3265 (5.53%)	259 (13.43%)	< 0.001	18,188 (22.99%)

*Note*: WC1, ≥ 85 cm for females and ≥ 90 cm for males; WC2, ≥ 95 cm for females and ≥ 100 cm for males. WC, waist circumference.

**Figure 1:**
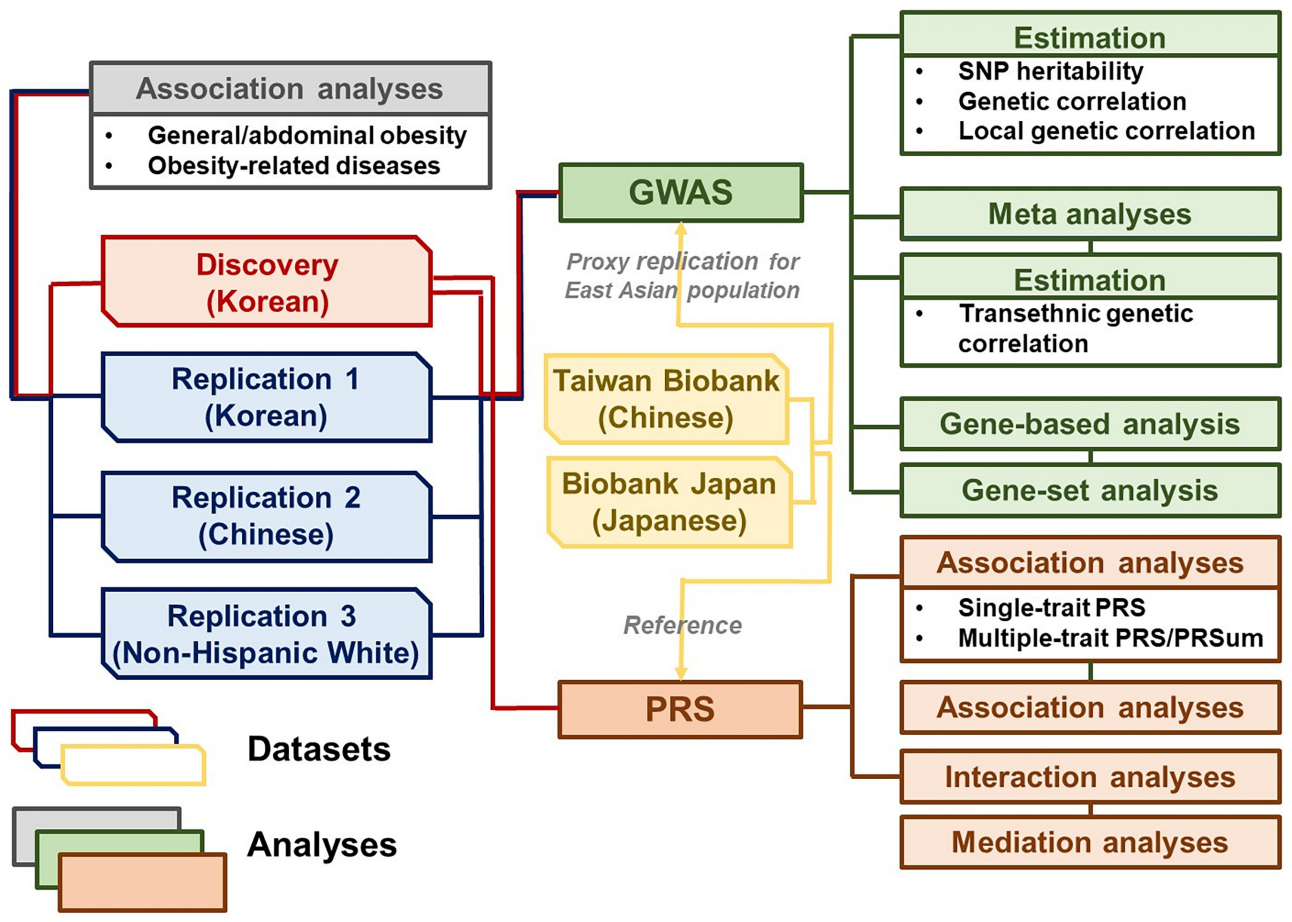
Analysis scheme This figure outlines the overall workflow of GWAS and PRS construction in both the discovery and replication datasets. GWAS, genome-wide association study; PRS, polygenic risk score.

Compared to individuals without obesity, individuals with obesity tended to be older, and the proportion of males with moderate obesity was notably higher. The obese group also displayed elevated levels of anthropometric measures (BMI, WC, body fat ratio, and abdominal fat ratio), and less favorable biochemical profiles, including elevated systolic blood pressure (SBP)/diastolic blood pressure (DBP), increased blood sugar (BS) for fasting plasma glucose (FPG), and unhealthy blood lipids levels of triglyceride (TG)/high density lipoprotein (HDL). A higher prevalence of obesity-related diseases was observed in this group. These trends were consistently observed across the replication datasets of the Korean, Chinese, and NHW populations, namely REP1_Kor_, REP2_Chi_, and REP3_NHW_, respectively (Tables S1–S3). An overview of the study and analysis framework is presented in **Figure 1**.

### GWASs, meta-analyses, and correlation analyses

**Figure 2:**
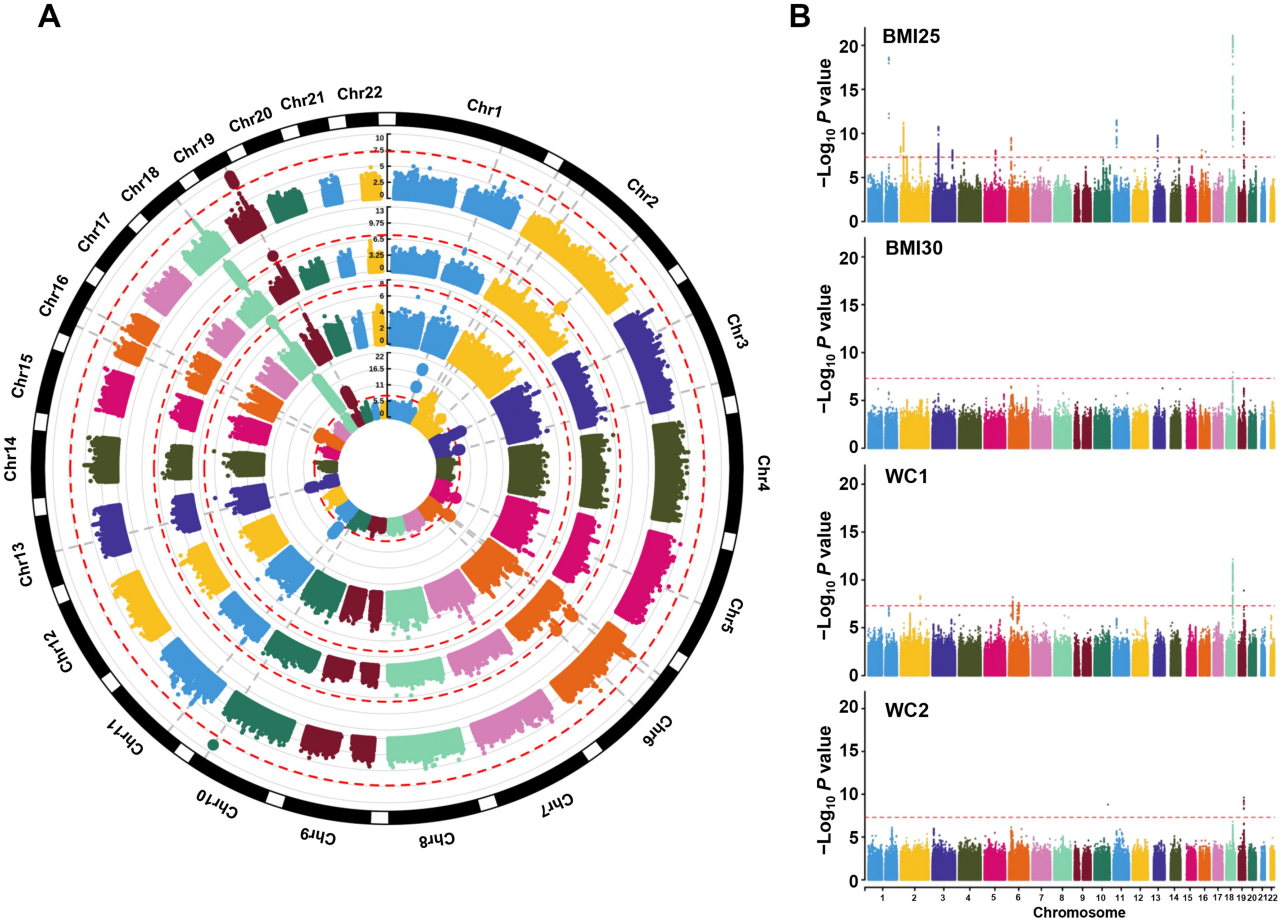
Manhattan plots constructed from GWAS results for obesity in the discovery dataset Circular and standard Manhattan plots are shown for four obesity phenotypes: BMI25 (BMI ≥ 25 kg/m^2^), BMI30 (≥ 30 kg/m^2^), WC1 (WC ≥ 85 cm for females / 90 cm for males), and WC2 (≥ 95 cm for females / 100 cm for males). The red dashed line indicates the genome-wide significance threshold. **A**. Circular plots arranged from inner to outer rings in the order of BMI25, BMI30, WC1, and WC2. **B**. Corresponding standard Manhattan plots. Chr, chromosome; BMI, body mass index; WC, waist circumference.

We conducted GWASs for obesity using discovery and three replication datasets, with Manhattan plots shown in **Figure 2** and Figures S1–S3. From the GWASs of general and abdominal obesity, we identified 24 genome-wide significant SNPs, of which 13 were replicated in REP1_Kor_ and/or REP3_NHW_, as shown in **Table 3**. Additionally, we included *P* values from the Biobank Japan (BBJ) and the Taiwan Biobank (TWB) as proxy replications to assess generalizability to other EAS populations, despite differences in outcome definitions. Most SNPs met the genome-wide significance thresholds. Notably, all 24 genome-wide significant SNPs have been reported to be associated with obesity-related indicators, such as BMI or WC, in prior studies, including those on populations of European ancestry.

**Table 3:** Significant SNPs identified from GWASs of obesity

Chr	Position	dbSNP ID	Ref/Alt	Gene	MAF	Trait	OR	STAT	*P* value	Replication *P* value			Proxy *P* value	
										REP1_Kor_	REP2_Chi_	REP3_NHW_	BBJ	TWB
1	177873210	**rs574367**	G/T	*SEC16B*	0.25	BMI25	1.12	9.01	2.49 × 10^−19^	**0.04**	0.75	**8.75 × 10^−38^**	5.32 × 10^−29^	4.79 × 10^−18^
2	629244	**rs12463617**	C/A	*TMEM18*	0.09	BMI25	0.90	−5.89	3.19 × 10^−9^	–	0.54	**4.18 × 10^−47^**	1.12 × 10^−16^	1.58 × 10^−22^
2	25109317	**rs7605335**	G/A	*ADCY3*	0.43	BMI25	1.08	6.88	6.26 × 10^−12^	**5.89 × 10^-5^**	–	–	9.55 × 10^−8^	3.02 × 10^−15^
2	54238794	**rs13395751**	G/C	*ACYP2*	0.01	BMI25	0.74	−5.39	3.88 × 10^−8^	–	–	**3.89 × 10^−4^**	–	–
2	54252986	rs6743455	G/A	*ACYP2*	0.38	BMI25	0.94	−5.50	3.76 × 10^−8^	0.44	0.35	0.15	7.85 × 10^−6^	2.29 × 10^−4^
2	169119609	rs4613239	C/G	*STK39*	0.30	WC1	1.08	5.87	4.63 × 10^−9^	0.15	–	–	–	3.09 × 10^−4^
2	169129145	**rs1955337**	G/T	*STK39*	0.30	BMI25	1.07	5.51	3.70 × 10^−8^	0.96	0.54	**0.05**	1.16 × 10^−9^	7.41 × 10^−6^
3	52886605	**rs2276825**	T/C	*TMEM110*	0.37	BMI25	1.08	6.73	1.76 × 10^−11^	**0.04**	0.70	**3.11 × 10^−7^**	6.60 × 10^−8^	7.76 × 10^−5^
3	173045367	rs9859660	T/C	NLGN1*	0.49	BMI25	1.06	5.77	7.96 × 10^−9^	–	**–**	–	9.37 × 10^−3^	0.01
5	95856501	**rs2611742**	T/C	*LOC101929710*	0.43	BMI25	1.06	5.76	8.73 × 10^−9^	0.19	0.50	**8.01 × 10^−7^**	1.92 × 10^−22^	6.92 × 10^−15^
6	20674691	**rs9368219**	C/T	*CDKAL1*	0.47	BMI25	0.94	−6.28	3.23 × 10^−10^	**0.02**	0.70	**7.37 × 10^−5^**	5.52 × 10^−28^	6.03 × 10^−5^
6	34225269	rs9394204	T/C	SMIM29*	0.18	WC1	1.09	5.83	6.47 × 10^−9^	0.96	–	–	–	1.12 × 10^−5^
6	85641628	rs10944051	T/C	TBX18*	0.35	WC1	1.07	5.58	2.48 × 10^−8^	–	0.08	0.07	–	0.91
10	117743698	rs150152426	T/C	ATRNL1*	0.01	WC2	0.25	−4.79	1.58 × 10^−9^	–	–	–	–	–
11	27749725	**rs1491850**	T/C	BDNF*	0.43	BMI25	0.93	−6.96	3.27 × 10^−12^	**0.04**	–	–	4.96 × 10^−24^	1.20 × 10^−11^
13	54084032	rs4883723	G/A	*LINC00558*	0.28	BMI25	1.08	6.39	1.75 × 10^−10^	–	–	–	1.89 × 10^−7^	3.55 × 10^−7^
16	20248599	**rs4281719**	A/G	*GP2*	0.22	BMI25	0.93	−5.75	8.08 × 10^−9^	0.10	0.87	**0.02**	9.00 × 10^−11^	8.32 × 10^−18^
16	53845487	rs11642841	C/A	*FTO*	0.03	BMI25	1.18	5.74	1.19 × 10^−8^	–	–	–	7.69 × 10^−32^	7.76 × 10^−13^
18	57838401	**rs663129**	G/A	*MC4R*	0.24	BMI25	1.13	9.62	7.95 × 10^−22^	**0.02**	0.22	**2.17 × 10^−62^**	4.20 × 10^−32^	5.50 × 10^−19^
18	57867526	**rs688671**	A/G	*MC4R*	0.26	BMI30	1.20	5.79	1.18 × 10^−8^	0.35	0.26	**1.72 × 10^−17^**	1.67 × 10^−22^	3.98 × 10^−12^
18	57867526	**rs688671**	A/G	*MC4R*	0.26	WC1	1.10	7.20	7.06 × 10^−13^	**0.04**	0.93	**1.16 × 10^−40^**	–	3.98 × 10^−9^
19	46177235	rs55669001	C/T	*GIPR*	0.47	BMI25	1.08	7.24	4.56 × 10^−13^	–	–	–	5.56 × 10^-39^	9.12 × 10^−7^
19	46200553	rs79953177	G/A	QPCTL*	0.42	WC2	0.84	−6.32	2.43 × 10^−10^	–	–	–	–	9.77 × 10^−6^
19	46221726	rs11881883	G/A	FBXO46*	0.40	WC1	0.93	−6.06	1.31 × 10^−9^	–	–	–	–	4.17 × 10^−7^

*Note*: SNPs with *P* values satisfying the replication threshold are in bold. * indicates gene regions that do not include genome-wide significant SNPs associated with the obesity-related indicators used to define obesity in the Trait column. Proxy *P* values refer to *P* values from BBJ and TWB for the obesity-related indicators used to define obesity in the Trait column. BBJ, Biobank Japan; TWB, Taiwan Biobank; MAF, minor allele frequency; OR, odds ratio; Kor, Korean; Chi. Chinese; NHW, non-Hispanic White.

For the obesity-related indicators, we identified 50 genome-wide significant SNPs (Table S4), 47 of which were replicated in at least one of the external datasets (REP1_Kor_, REP2_Chi_, REP3_NHW_, BBJ, or TWB). Four of the SNPs (rs220063, rs11881883, rs2296083, and rs4817972) appeared to be novel. As shown in Figures S4 and S5, GWASs for continuous outcomes showed broader ranges of significance and more widespread associated regions across chromosomes than dichotomous obesity. Nevertheless, some SNPs, such as rs9394204, were significant only in GWASs for the dichotomous outcomes.

For obesity-related diseases, we identified 58 genome-wide significant SNPs: 17 for HT, 14 for T2D, 1 for CVD, 15 for hyperlipidemia (HL), and 1 for fatty liver (FL), as shown in Figures S6 and S7 and Table S5. Among these, 19 SNPs were successfully replicated: 4 for HT, 12 for T2D, and 3 for HL. One SNP (rs6499554) associated with HL was novel, whereas two replicated SNPs (rs35612982 in *CDKAL1* and rs66723169 in *MC4R*) were located in overlapping gene regions with obesity. Most SNPs relevant to obesity-related indicators, obesity, and obesity-related diseases in the Korean populations also generalized well to Japanese and Chinese of EAS populations.

As shown in Table S6, genomic inflation factors ($\lambda_{\mathrm{GC}}$) exceeded 1 for all obesity-related indicators, suggesting polygenicity or population structure. However, LD score regression intercepts ($\lambda_{\mathrm{LDSC}}$) were close to 1, indicating no major confounding due to population stratification, while those for continuous traits in REP3_NHW_ were higher, likely due to the large sample size and polygenicity. The SNP heritability estimates were significant for all traits except FL in the large-scale datasets of discovery and REP3_NHW_. Notably, moderate obesity showed higher SNP heritability in the discovery and REP3_NHW_ datasets. In contrast, the estimates in REP1_Kor_ and REP2_Chi_ were mostly negative or non-significant, likely due to the small sample size.

Regarding genetic correlations, obesity was strongly associated with target obesity-related indicators. For BMI-based traits, *GC*_BMI–BMI25_ = 1.01 (*P* = 0) and *GC*_BMI–BMI30_ = 0.94 (*P* = 2.26 × 10^−59^), and for WC-based traits, *GC*_WC–WC1_ = 0.97 (*P* = 0) and *GC*_WC–WC2_ = 0.90 (*P* = 3.33 × 10^−39^), indicating overlapping genetic architectures. Similarly, significant correlations were observed between obesity and its related diseases (Table S6). Among Korean individuals, abdominal obesity showed stronger genetic correlations with related diseases, whereas in the NHW populations, no consistent trend was observed.

Local genetic correlation patterns (Figures S8 and S9; Table S7) revealed obesity–disease correlated regions that were mostly Korean population-specific but showed non-overlapping genomic regions with GWASs of obesity or obesity-related diseases. Transethnic genetic correlation analyses between the EAS meta-analyses and the REP3_NHW_ dataset showed strong correlations with moderate levels of general obesity (0.82, *P* = 4.01 × 10^−3^) and abdominal obesity (0.72, *P* = 6.37 × 10^−4^), suggesting that genetic signals relevant to obesity in Koreans may be significant in NHW populations.

### Gene-based and gene-set analyses

Gene-based analyses were performed using the GWAS results for obesity and related diseases in the discovery dataset. Table S8 presents the genes that reached statistical significance level (α = 2.83 × 10^−6^) adjusted by Bonferroni correction under various definitions of obesity and five obesity-related diseases. While no novel genes were identified for obesity, ten novel genes were associated with two diseases: eight for HT (*SPTBN1*, *STXBP1*, *PTRH1*, *TTC16*, *ARL3*, *SFXN2*, *INA*, and *PDCD11*) and two for HL (*APOA4* and *HP*). No genes were significantly associated with CVD. Notably, most of these genes were replicated in the REP3_NHW_ dataset, supporting the existence of shared genetic mechanisms across populations of different ancestries.

For gene-set analyses, we constructed two sets: one comprising 31 genes related to obesity, and the other including 64 genes associated with obesity-related diseases. CVD was excluded due to the lack of suitable Gene Expression Omnibus (GEO) target data. As summarized in Table S9, 5 of the 24 gene-set combinations showed significant associations, primarily from disease-related gene sets. In contrast, the obesity gene set based on significant genes from gene-based analyses did not exhibit significant enrichment in any of the tested combinations. Further details for HT, HL, and FL are provided in Table S10. These results suggest that the significant gene sets are primarily linked to lipid metabolism pathways driven by specific genes. This indicates that, at the pathway level, obesity gene sets may not directly contribute to the development of associated diseases. These findings highlight the distinction between gene- and pathway-level associations, suggesting that a shared genetic etiology may manifest differently across biological contexts.

### Construction and evaluation of single-trait PRS models for obesity

The prevalence patterns of obesity and obesity-related diseases were consistent between the validation and test datasets, as shown in Table S11. To explore the genetic similarities among EAS populations, we estimated SNP heritability and genetic correlations for obesity-related indicators and metabolic traits across Korean, Japanese, and Chinese populations. The results showed that all traits were mostly heritable in EAS populations, with most exhibiting significantly high genetic correlations (Table S12). Moreover, strong cross-population correlations for key indicators such as BMI and WC were observed (*GC*_BBJ–KOR_ = 0.90, *P* = 0 for BMI; *GC*_TWB–KOR_ = 0.92, *P* = 1.23 × 10^−243^ for BMI; *GC*_T__WB–KOR_ = 0.94, *P* = 3.59 × 10^−128^ for WC) (Table S12), supporting the suitability of the PRS derivation based on these populations for use in the Korean dataset.

In the PRS analyses, the results for obesity from the validation phase (Table S13) indicated that models built using the LDpred2 auto algorithm achieved the best performance across all selection metrics. In addition, we constructed PRSs for metabolic health indicators. The best-performing models for each trait were selected based on the validation results, as shown in Table S14.

### Construction and performance comparison of single-trait and multiple-trait PRS models for obesity and obesity-related diseases

The best-performing PRS models selected during validation were subsequently applied to the test dataset. As shown in Table S15, the single-trait PRS model for BMI (BMI PRS; Model 1) outperformed the single-trait PRS model for WC (WC PRS; Model 2) in predicting general obesity, with significantly higher area under the curve (AUC) values for both BMI25 (DeLong *P* = 1.64 × 10^−18^) and BMI30 (DeLong *P* = 7.76 × 10^−7^). In contrast, the WC PRS did not exhibit significant improvements in predictive performance for any obesity outcome or obesity-related disease, except for T2D (DeLong *P* = 2.40 × 10^−4^).

In comparison, the multiple-trait PRS model (Model 3), which incorporated both obesity metrics (BMI and WC), achieved significantly higher AUCs for obesity and HT than the single-trait PRS models (Model 1 and Model 2). Furthermore, an extended multiple-trait PRS model (Model 4), which included additional PRSs for metabolic health indicators, demonstrated improved predictive performance for obesity-related diseases, except CVD, but did not enhance the prediction for obesity outcomes.

### Association analyses of PRSs on obesity and obesity-related diseases

The associations between PRSs and obesity/obesity-related diseases are summarized in **Table 4** and **Table 5**. These results showed consistent trends in risk across PRS categories [low (L), medium (M), and high (H)] for both single- and multiple-trait models.

**Table 4:** Effect of single-trait PRS on obesity and obesity-related diseases

Trait	PRS group	BMI PRS			WC PRS		
		Case count (prevalence)	OR (95% CI)	*P* value	Case count (prevalence)	OR (95% CI)	*P* value
BMI25	L	1494 (21.43%)	0.54 (0.51, 0.58)	**3.61 × 10^−87^**	1726 (24.76%)	0.65 (0.61, 0.68)	**1.71 × 10^−49^**
	M (Ref)	18,508 (33.16%)	–	–	18,613 (33.35%)	–	**–**
	H	3353 (48.06%)	1.90 (1.80, 2.00)	**4.00 × 10^−135^**	3016 (43.18%)	1.54 (1.46, 1.62)	**1.12 × 10^−60^**
BMI30	L	80 (1.15%)	0.41 (0.32, 0.51)	**6.39 × 10^−15^**	103 (1.48%)	0.50 (0.41, 0.61)	**2.11 × 10^−11^**
	M (Ref)	1549 (2.78%)	–	**–**	1598 (2.86%)	–	**–**
	H	441 (6.32%)	2.36 (2.11, 2.63)	**7.55 × 10^−54^**	369 (5.28%)	1.91 (1.70, 2.15)	**1.12 × 10^−27^**
WC1	L	1188 (18.22%)	0.65 (0.61, 0.70)	**1.15 × 10^−36^**	1192 (18.21%)	0.64 (0.60, 0.68)	**4.05 × 10^−39^**
	M (Ref)	13247 (25.33%)	–	–	13,250 (25.32%)	–	**–**
	H	2279 (34.71%)	1.59 (1.51, 1.68)	**5.43 × 10^−61^**	2272 (34.88%)	1.61 (1.52, 1.70)	**9.37 × 10^−63^**
WC2	L	121 (1.86%)	0.52 (0.43, 0.62)	**2.88 × 10^−12^**	126 (1.92%)	0.52 (0.43, 0.62)	**1.37 × 10^−12^**
	M (Ref)	1844 (3.53%)	–	**–**	1868 (3.57%)	–	**–**
	H	436 (6.64%)	1.96 (1.75, 2.18)	**4.90 × 10^−34^**	407 (6.25%)	1.83 (1.64, 2.05)	**1.39 × 10^−26^**
HT	L+M (Ref)	12,022 (22.07%)	–	–	12,028 (22.04%)	–	–
	H	1507 (24.4%)	1.18 (1.10, 1.25)	**8.94 × 10^−7^**	1501 (24.7%)	1.20 (1.12, 1.28)	**5.03 × 10^−8^**
T2D	L+M (Ref)	4478 (7.78%)		–	4429 (7.69%)	–	**–**
	H	537 (8.36%)	1.10 (1.00, 1.21)	**0.05**	586 (9.21%)	1.23 (1.12, 1.35)	**1.25 × 10^−5^**
CVD	L+M (Ref)	2504 (4.67%)	–	–	2490 (4.63%)	–	–
	H	289 (4.74%)	1.05 (0.92, 1.19)	0.47	303 (5.07%)	1.10 (0.97, 1.25)	0.13
HL	L+M (Ref)	6166 (11.39%)	–	–	6136 (11.32%)	–	–
	H	650 (10.63%)	0.93 (0.85, 1.01)	0.11	680 (11.29%)	1.01 (0.93, 1.10)	0.73
FL	L+M (Ref)	2553 (5.73%)	–	–	2563 (5.73%)	–	–
	H	324 (6.4%)	1.13 (1.00, 1.28)	**0.04**	314 (6.39%)	1.11 (0.98, 1.26)	0.08

*Note*: L, M, and H indicate low (bottom decile), medium (2nd–9th deciles), and high (top decile) PRS groups, respectively. OR and *P* values of PRS groups from logistic model on obesity are adjusted by age, sex, PRS group, and the first five principal components. *P* values satisfying the replication threshold are in bold. PRS, polygenic risk score; CI, confidence interval; OR, odds ratio; Ref, reference.

**Table 5:** Effect of multiple-trait PRSsum on obesity and obesity-related diseases

Trait	PRS group	PRSsum1			PRSsum2		
		Case count (prevalence)	OR (95% CI)	*P* value	Case count (prevalence)	OR (95% CI)	*P* value
BMI25	L	1446 (20.74%)	0.52 (0.49, 0.55)	**7.31 × 10^−100^**	1722 (24.73%)	0.65 (0.62, 0.69)	**1.33 × 10^−47^**
	M (Ref)	18,539 (33.21%)	–	–	18,575 (33.27%)	–	**–**
	H	3370 (48.3%)	1.91 (1.81, 2.01)	**1.92 × 10^−137^**	3058 (43.84%)	1.58 (1.50, 1.66)	**7.63 × 10^−69^**
BMI30	L	85 (1.22%)	0.44 (0.35, 0.55)	**3.42 × 10^−13^**	113 (1.62%)	0.57 (0.47, 0.68)	**7.86 × 10^−9^**
	M (Ref)	1509 (2.70%)	–	**–**	1577 (2.82%)	–	**–**
	H	476 (682%)	2.65 (2.38, 2.95)	**6.38 × 10^−72^**	380 (5.45%)	1.98 (1.77, 2.22)	**1.74 × 10^−31^**
WC1	L	1094 (16.76%)	0.58 (0.54, 0.62)	**1.11 × 10^−53^**	1309 (20.02%)	0.73 (0.68, 0.78)	**4.58 × 10^−22^**
	M (Ref)	13,226 (25.27%)	–	–	13,219 (25.27%)	–	**–**
	H	2394 (36.72%)	1.74 (1.65, 1.84)	**3.22 × 10^−87^**	2186 (33.4%)	1.49 (1.41, 1.58)	**6.30 × 10^−45^**
WC2	L	105 (1.61%)	0.44 (0.36, 0.54)	**1.03 × 10^−15^**	139 (2.13%)	0.58 (0.49, 0.69)	**1.50 × 10^−9^**
	M (Ref)	1829 (3.49%)	–	**–**	1851 (3.54%)	–	**–**
	H	467 (7.16%)	2.15 (1.93, 2.38)	**1.17 × 10^−45^**	411 (6.28%)	1.82 (1.63, 2.04)	**1.48 × 10^−26^**
HT	L+M (Ref)	11,984 (21.98%)	–	**–**	11,722 (21.53%)	–	–
	H	1545 (25.2%)	1.23 (1.16, 1.31)	**1.91 × 10^−10^**	1807 (29.1%)	1.58 (1.48, 1.68)	**5.25 × 10^−48^**
T2D	L+M (Ref)	4393 (7.63%)		–	4372 (7.59%)		**–**
	H	622 (9.76%)	1.33 (1.21, 1.45)	**9.65 × 10^−10^**	643 (10.09%)	1.39 (1.27, 1.52)	**2.59 × 10^−13^**
CVD	L+M (Ref)	2493 (4.64%)	–	**–**	2466 (4.6%)	–	**–**
	H	300 (4.97%)	1.08 (0.95, 1.22)	0.25	327 (5.37%)	1.21 (1.07, 1.37)	**2.00 × 10^−3^**
HL	L+M (Ref)	6116 (11.29%)	–	**–**	6036 (11.15%)	–	**–**
	H	700 (11.53%)	1.04 (0.95, 1.13)	0.38	780 (12.71%)	1.17 (1.08, 1.27)	**1.06 × 10^−4^**
FL	L+M (Ref)	2542 (5.69%)	–	**–**	2522 (5.66%)	–	**–**
	H	335 (6.76%)	1.20 (1.07, 1.36)	**2.22 × 10^−3^**	355 (7.06%)	1.27 (1.13, 1.43)	**4.25 × 10^−5^**

*Note*: PRSsum1 stands for the sum of standardized PRSs for BMI and WC; PRSsum2 stands for the sum of standardized PRSs for BMI, WC, SBP, DBP, BS for FPG, TG, and HDL-C. OR and *P* values of PRS groups from logistic model on obesity are adjusted by age, sex, PRS group, and the first five principal components. *P* values satisfying the replication threshold are in bold. BS, blood sugar; HDL-C, high-density lipoprotein cholesterol.

Table 4 presents the effects of the single-trait PRSs for BMI and WC. Across all obesity types, individuals in the L group showed a decreased risk, whereas those in the H group showed an increased risk. Notably, the BMI PRS more clearly differentiated the risk of general obesity, while the WC PRS showed similar levels of risk for abdominal obesity. Both PRSs were significantly associated with HT and T2D in the H group. The BMI PRS was significantly associated with FL in the H group, with an odds ratio (OR) of 1.13 (*P* = 0.04). Interestingly, in the H group, the WC PRS showed a stronger association with T2D (OR = 1.23, *P* = 1.25 × 10^−5^) than the BMI PRS (OR = 1.10, *P* = 0.05).

In Table 5, risk associations are stratified by multiple-trait PRSs: PRSsum1 for obesity-related indicators only and PRSsum2 for obesity-related indicators along with metabolic health indicators. Both models maintained consistent directional trends across obesity outcomes, with reduced ORs in the L group and elevated ORs in the H group. PRSsum1 showed stronger associations with obesity-related diseases than the single-trait PRSs. PRSsum2 further amplified these effects, with significantly increased risks observed for HT (OR = 1.58, *P* = 5.25 × 10^−48^), T2D (OR = 1.39, *P* = 2.59 × 10^−13^), and FL (OR = 1.27, *P* = 4.25 × 10^−5^). Additionally, associations with CVD (OR = 1.21, *P* = 2.00 × 10^−3^) and HL (OR = 1.17, *P* = 1.06 × 10^−4^), previously non-significant in single-trait models, emerged as significant in PRSsum2.

**Figure 3:**
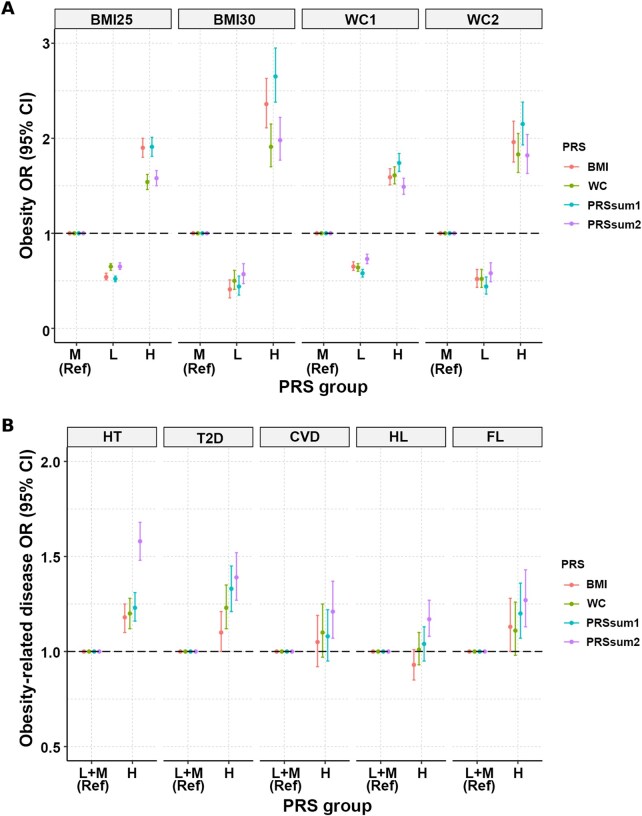
PRS associations on obesity and obesity-related diseases **A**. Associations of PRSs with obesity. **B**. Associations of PRSs with obesity-related diseases. L, M, and H represent low, medium, and high PRS groups of the bottom, middle (2nd–9th), and top deciles, respectively. PRSsum1 indicates the sum of standardized PRSs for BMI and WC, while PRSsum2 indicates the sum of standardized PRSs for BMI, WC, and five metabolic health traits, including SBP, DBP, BS for FPG, TG, and HDL-C. HT, hypertension; T2D, type 2 diabetes; CVD, cardiovascular disease; HL, hyperlipidemia; FL, fatty liver; OR, odds ratio; CI, confidence interval; SBP, systolic blood pressure; DBP, diastolic blood pressure; BS, blood sugar; FPG, fasting plasma glucose; TG, triglyceride; HDL-C, high-density lipoprotein cholesterol.

These findings suggest that multiple-trait PRSs can effectively capture the complex genetic architectures underlying obesity and its comorbidities. The comparative effects of both types of PRSs are visualized in **Figure 3**. These results reinforce the utility of composite genetic models for risk stratification in clinical settings, particularly in EAS populations.

### Interaction and mediation analyses of PRSs for obesity on obesity-related diseases

To evaluate the interactions between obesity and obesity-related diseases, we calculated and validated the PRSs for T2D and CVD, as shown in Table S16. We then assessed the interactions between obesity (defined either by obesity status or PRS) and the corresponding disease PRSs in T2D. As shown in Table S17, both the target disease PRS and obesity classification were highly significant predictors in all regression models. In contrast, the obesity PRSs showed relatively weaker or no significance, with most effects being non-significant. Interestingly, we observed significant interaction effects between obesity status and T2D PRS, indicating antagonistic interactions, while both obesity and T2D PRS independently increased disease risk, their interaction term was associated with a reduction in that risk. In CVD, similar trends were observed, although the interaction effects were not statistically significant, and only some associations with obesity PRS reached significance.

We performed mediation analyses to disentangle the direct and indirect effects of obesity PRSs on T2D and CVD risks. Overall, the mediation effects were significant. Notably, for T2D, mediation effects were significant only when adjusting for disease PRS, whereas for CVD, significant effects were observed both with and without adjustment. Regardless of the significance of direct effects, the indirect mediation effects of obesity PRSs via obesity were consistently significant. This suggests a strong mediation pathway from obesity PRSs to obesity-related diseases, indicating that obesity PRSs exert independent effects on their related comorbidities even after adjusting for the genetic correlations between them. Specifically for CVD with CVD PRS adjustment, the mediation proportion of BMI-related obesity from BMI PRS was 77.96% (*P* = 0.032), and that of WC-related obesity from WC PRS was 40.40% (*P* = 0.002), while the mediation proportion of obesity from PRSsum1 was lower than the BMI-related and higher than WC-related effects (Table S17). These findings highlight the role of obesity as a key intermediate phenotype linking genetic risk factors to cardiometabolic outcomes, particularly in EAS populations.

## Discussion

In this study, we investigated the genetic factors underlying obesity and their influence on obesity-related diseases in a large Korean cohort, representative of the EAS populations. We hypothesized that a genetic predisposition to obesity is shared between general and abdominal obesity, as well as across the EAS and NHW populations, despite differences in genetic backgrounds. Furthermore, we compared the performance of traditional single-trait PRSs with that of multiple-trait approaches, including the PRSsum method, to evaluate their predictive accuracy and potential for identifying shared genetic components. Using the PRS-derived risk profiles, we further explored the mechanisms by which genetic susceptibility to obesity contributes to disease onset through interaction and mediation analyses.

We commenced our investigation by conducting GWASs for two obesity-related indicators, two definitions of obesity (each with two severity levels), and five obesity-related diseases. Our analyses revealed 50 genome-wide significant SNPs for obesity-related indicators, of which 47 were replicated across the populations. These included 34 SNPs for BMI and 13 for WC, with 5 overlapping SNPs (Table S4). Among these, three SNPs, namely rs220063 (*ZEB1*), rs11881883 (*FBXO46*), and rs4817972 (*LOC400867*), were novel. Although these genes have not been directly associated with obesity in previous studies, some literature suggests their indirect relevance. *ZEB1* and *FBXO46* have been associated with BMI [34,35], and CpG representing the LOC400867 gene has been associated with birth weight [36]. In the GWAS of obesity, we identified 24 genome-wide significant SNPs (Table 3), of which 13 were replicated: 12 for general obesity and one for abdominal obesity, with one overlapping. When comparing the GWAS results for continuous and dichotomized outcomes, we observed substantial overlap, with genetic correlations exceeding 0.90 (Figures S4 and S5). Continuous traits exhibited a broader range of associations, whereas dichotomous outcomes retained unique findings, highlighting the importance of considering multiple definitions of a target.

In our analysis of obesity-related diseases, we identified 58 genome-wide significant SNPs (Table S5), including one novel locus (rs6499554 in *PKD1L3*) associated with HL. The overlap between obesity- and disease-associated SNPs was limited, with only two SNPs located in shared gene regions (*CDKAL1* and *MC4R*). Nevertheless, EAS–NHW transethnic genetic correlations were significant: 0.82 for general obesity and 0.72 for abdominal obesity, demonstrating transferability of genetic signals across EAS and NHW populations (Table S6). Furthermore, EAS populations exhibited higher correlations between abdominal obesity and obesity-related diseases than their NHW counterparts. The distinct patterns of local genetic correlations are shown in Figures S8 and S9 and Table S7. Gene-based analyses identified significant genes for both obesity and its related diseases (Table S8), including *CDKAL1* as a shared gene between general obesity and T2D. Gene-set analyses (Tables S9 and S10) revealed significant enrichment, suggesting limited overlap at the fatty acid metabolism pathway level. From the similarities in gene-based analyses and the dissimilar patterns in local genetic correlations between obesity and obesity-related diseases, these findings emphasize the need for ethnicity-specific approaches to decipher the genetic architecture of obesity and related disorders.

Using the single-trait PRSs derived from both BMI and WC, we confirmed that individuals in the high-risk groups showed increased susceptibility to obesity, HT, T2D, and FL (Table 4). The WC PRS was particularly associated with T2D, while the BMI PRS showed a stronger association with FL. Multiple-trait PRSs demonstrated improved predictive accuracy (Table S15), particularly when combining PRSs for obesity-related indicators and metabolic health traits (PRSsum2), which further enhanced the prediction of disease outcomes such as HT, T2D, and CVD (Table 5). These findings suggest that multiple-trait PRSs better capture the polygenic architecture of comorbidities than single-trait PRSs, especially when the PRS traits and outcome are aligned. To further understand the biological mechanisms linking the genetic predisposition of obesity to disease, we performed interaction and mediation analyses on obesity-related diseases, such as T2D and CVD (Table S17). Although the interactions between obesity PRSs and disease PRSs were mostly not significant, we observed significant interactions between the obesity phenotypes and disease PRSs. Mediation analyses revealed that the total effects of obesity PRSs on T2D and CVD were partially significant and primarily driven by the indirect effects through obesity. In particular, the majority of the direct effects on disease were non-significant, suggesting major mediation through obesity in the order of BMI PRS, PRSsum1, WC PRS, and PRSsum2.

In summary, in the Korean population, we identified genetic variants associated with obesity-related indicators, obesity classification, and obesity-related diseases. Among these, obesity-related indicators and obesity classification showed largely overlapping associations with only minor differences, whereas obesity-related diseases exhibited distinct signals from significant genetic correlations observed across GWASs, gene-based analyses, and local genetic correlation analyses. Furthermore, PRS analyses revealed that single-trait BMI/WC PRSs showed strong predictive power for obesity classification but only a modest effect for obesity-related diseases, while multiple-trait PRSs captured additional genetic components not explained by obesity PRS alone. In combination with the interactions and mediation effects of obesity, we concluded that obesity and its related diseases have both independent genetic effects and interconnected pathways, emphasizing that obesity acts both as a mediator and a moderator in its related diseases. Although our findings provide novel insights, several limitations should be acknowledged. First, the study population was limited to Koreans. Although genetic similarity among the EAS populations was supported by high cross-population correlations (Table S12), subtle population-specific differences may affect generalizability. Second, while multiple-trait PRSs improved the performance for many outcomes, the optimal PRS composition for each phenotype requires further evaluation. Third, we still not sure which obesity indicator is the best among obesity-related indicators due to the absence of consistent superiority among BMI, WC, and others in the EAS populations. Despite these limitations, our study, which is the largest genetic study of obesity in the Korean population to date, offers valuable insights into the shared genetic architecture of obesity and related diseases. These findings support the development of population-specific polygenic models to inform precision medicine initiatives.

## Materials and methods

### Research participants and genotype data processing

A comprehensive overview of the study population and genotype data has been described previously [33], and here we provide a brief summary tailored to the present analysis. Genotype data were obtained from a total of 231,302 participants enrolled in four Korean cohorts, all genotyped using the Korean Biobank Array (Koreanchip) [37]. These cohorts include the Korean Genome Epidemiology Study (KoGES; https://nih.go.kr/contents.es?mid=a50401010100) [38], Gene-Environment of Interaction and Phenotype (GENIE; http://en-healthcare.snuh.org/HPEACEstudy) [39], YonSei University Hospital medical center (YSUH), and the Veterans Health Service Medical Center (VHSMC). For replication purposes, we incorporated three independent datasets: (1) REP1_Kor_, an additional Korean subset from KoGES genotyped with Affymetrix Arrays (*n* = 8856); (2) REP2_Chi_, a dataset comprising individuals of Chinese ancestry (*n* = 1503) from the UK Biobank (UKB) [40]; and (3) REP3_NHW_, a large-scale dataset of European ancestry (*n* = 459,259) from the UKB.

For the discovery and REP1_Kor_ datasets, genotype data underwent standardized quality control (QC) procedures. These included filtering at both the individual level (missing genotype call rate > 0.05 or sex inconsistency) and the SNP level [missing genotype call rate or Hardy-Weinberg equilibrium (HWE) *P* < 1 × 10^−5^]. Phasing was performed using Eagle (v2.4) [41], and imputation was carried out using the Northeast Asian Reference Database imputation server [42]. Additional QC steps were applied to exclude individuals with excessive SNP heterozygosity or mean difference in the top two principal components (PCs) > 5 × inter-quantile range (IQR) and to remove SNPs with *R*^2^ < 0.3, non-biallelic variants, missing call rate > 0.05, minor allele frequency (MAF) < 0.005 (Koreanchip) or < 0.05 (Affymetrix), or HWE *P* < 1 × 10^−5^. These steps were conducted using PLINK [43,44] and GCTA [45]. For the REP2_Chi_ and REP3_NHW_ datasets, QC was performed on the downloaded imputed data. SNPs with missing call rate > 0.01, HWE *P* < 1 × 10^−6^, or non-biallelic status were excluded. Participants of Chinese and NHW (coded as White, British, Irish, or any other White background) were retained. Further filtering removed SNPs with MAF < 0.05 (Chinese) or < 0.005 (NHW), and individuals with SNP heterozygosity or mean difference in the top two PCs > 5 × IQR or estimated genetic relatedness > 0.125 (*i.e.*, closer than second-degree relatives).

After applying harmonized inclusion and exclusion criteria, the resulting datasets included a discovery dataset of 85,947 Korean individuals with 4,736,957 SNPs, and three replication datasets consisting of 7726 Koreans with 2,221,602 SNPs, 1496 Chinese individuals with 2,076,717 SNPs, and 392,160 NHW individuals with 4,936,389 SNPs.

### Anthropometric indicators of obesity and obesity-related diseases

Obesity-related phenotypes were evaluated based on BMI and WC following the criteria established for the Korean population [12,14]. For general obesity, individuals of EAS ancestry were classified as obese (case) if their BMI ≥ 25 kg/m^2^, whereas NHW individuals were considered obese at BMI ≥ 30 kg/m^2^. Individuals below these thresholds were categorized as controls (referred to as BMI25 and BMI30 criteria, respectively). Severe obesity was further stratified as BMI ≥ 30 kg/m^2^ for EAS populations (BMI30) and BMI ≥ 40 kg/m^2^ for NHW populations (BMI40). Additionally, abdominal obesity was defined uniformly across all ancestry groups as WC ≥ 85 cm in females and ≥ 90 cm in males (WC1) for moderate level, and WC ≥ 95 cm for females or ≥ 100 cm for males (WC2) for severe level, owing to the absence of ancestry-specific guidelines for this trait. While the BMI-based definitions were adjusted for ancestry, the WC-based definitions remained consistent across populations.

For obesity-related diseases, we assessed five conditions at baseline: HT, T2D, CVD, HL, and FL. CVD encompassed both coronary heart disease and cerebrovascular events. Disease status was primarily determined based on self-reported diagnoses. In case of T2D, diagnosis was based either on self-report or on meeting one or more of the following clinical criteria: (1) FPG level ≥ 126 mg/dl, (2) 2-h postprandial plasma glucose ≥ 200 mg/dl, or (3) glycated hemoglobin of HbA1c ≥ 6.5%.

### GWASs, meta-analyses, and correlation analyses

GWASs were conducted using a modified version of our previously described analytical framework [33], tailored to the design of the present study. We aimed to identify genetic variants associated with obesity, its related indicators, and comorbidities using the discovery dataset. Specifically, for obesity-related indicators, we applied a rank-based inverse normal transformation. Linear and logistic regression models were applied using PLINK and REGENIE [46], respectively, with age, sex, and the top ten PCs as covariates. A genome-wide significance threshold was set at α = 5 × 10^−8^. To reduce redundancy, SNPs in high linkage disequilibrium (LD; *r*^2^ > 0.1) with top signals were excluded. The remaining lead SNPs were annotated using ANNOVAR [47]. All three replication datasets were used to replicate the GWAS findings related to obesity and its related indicators. To evaluate the generalizability of the Korean-derived associations to other EAS populations, we compared the results with publicly available GWAS summary statistics for BMI from the BBJ (http://jenger.riken.jp/en) [48] and for BMI/WC from the TWB (https://www.ebi.ac.uk/gwas/publications/38116116) [49]. For obesity-related diseases, SNP validation was performed primarily in the REP3_NHW_ dataset and partially in the BBJ dataset, limited to T2D and CVD, focusing on large-scale data sources.

Summary-level GWAS results were analyzed using LDSC [50] to assess genomic inflation and to estimate SNP-based heritability. Genetic correlations between obesity-related indicators and between these traits and obesity were quantified in the discovery dataset using LDSC. In addition, correlations between obesity and obesity-related diseases were evaluated using both the discovery and REP3_NHW_ datasets. Although LDSC was used to estimate genome-wide correlations, local genetic correlations were estimated using LAVA [51]. To explore the shared genetic architecture between the EAS and NHW populations, we conducted meta-analyses using GWAMA [52], integrating summary statistics from the discovery and two replication datasets (REP1_Kor_ and REP2_Chi_). With the restriction to SNPs having an effective sample size greater than the mean EAS sample size (*n* = 69,651), transethnic genetic correlations were further examined for moderate obesity (BMI25/WC1 for the EAS population and BMI30/WC1 for the NHW population) using POPCORN [53].

### Gene-based and Gene-set analyses

We performed gene-based association analyses across 18,432 genes using the discovery dataset, REP1_Kor_, and REP2_Chi_. These analyses were conducted using MAGMA [54] by applying the 1000G EAS reference panel. For analysis of the REP3_NHW_ dataset, 1000G EUR reference data were used to account for ancestry-specific genetic structures.

To further examine the biological relevance of obesity- and disease-associated genes, we conducted gene-set analyses focusing on HT, T2D, and HL. This process involved three key steps. First, we defined two separate gene sets derived from the significant gene-based results: one for obesity (BMI25/WC1) and the other for obesity-related diseases (HT, T2D, HL, and FL). Second, we extracted gene expression data from four GEO datasets (GSE24752 for HT, GSE278204 for T2D, GSE1010 for HL, and GSE63067 for FL) and calculated log-fold changes using the DESeq2 package (v1.46.0) [55]. Finally, we performed two types of gene-set analyses using R: (1) gene set enrichment analysis (GSEA) using the fgsea package (v1.32.2) [56] based on the Reactome database; and (2) over-representation analysis (ORA) with the clusterProfiler package (v4.14.6) [57], referring to both GO and KEGG databases.

### Construction and evaluation of single-trait PRS models for obesity

To evaluate genetic susceptibility to obesity, we constructed PRSs using genome-wide summary statistics from the BBJ and the TWB. Variants with MAF > 0.005 were retained, resulting in 5,925,388 SNPs from the BBJ and 7,614,251 SNPs from the TWB for downstream analysis. These sets were used as sources for PRS construction. Multiple widely used PRS methods have been applied, including clumping and thresholding (CT) [58], LDpred2 (infinitesimal, grid, and auto models) [59,60], lassosum [61], and PRS-CS [62].

For CT, we applied a significance threshold of *P* < 1 × 10^−5^ for selecting index SNPs from summary statistics, and clumped SNPs using PLINK with a LD threshold of *r*^2^ = 0.5 and a physical distance of 250 kb. For all LDpred2 models, we used the bigsnpr package (v1.12.18) [63], and SNP heritability was estimated from LDSC. Specifically, for the LDpred2 grid model, we tested various prior probabilities for the proportion of causal variants (*ρ* = 1%, 3%, 10%, 30%, and 100%). For the LDpred2 auto model, we initialized 20 logarithmically spaced values of *ρ* ranging from 1 × 10^−4^ to 1. For lassosum, we used the default shrinkage parameter vector (*s* = 0.2, 0.5, 0.9, and 1.0) and set the maximum reference sample size to 10,000, using the lassosum package (v0.4.5). For PRS-CS, we applied the default gamma-gamma prior (*a* = 1.0, *b* = 0.5) and set the global shrinkage parameter to *ϕ* = 0.01. All LD estimates were obtained using the EAS reference panel from the 1000 Genomes Project.

For model evaluation, we split the discovery dataset by randomly assigning 15,000 individuals for validation and 70,947 individuals for testing. During the validation phase, 10-fold cross-validation was conducted using linear or logistic regression models, incorporating age, sex, corresponding PRS, and the first five PCs as covariates. The optimal PRS for each method was selected based on multiple performance criteria: (1) strength of correlation with obesity-related traits, (2) statistical significance, (3) model fit assessed using Akaike Information Criterion (AIC), and (4) predictive accuracy, as indicated by the AUC for binary outcomes.

### Construction and evaluation of multiple-trait PRS models for obesity and obesity-related diseases

To enhance the genetic prediction of obesity and its comorbidities, we implemented a multiple-trait PRS strategy [64] that incorporates information across several obesity-related traits. Specifically, we constructed PRSs for two primary obesity-related indicators (BMI and WC) and five metabolic traits (SBP, DBP, BS for FPG, TG, and HDL-C). These trait-specific PRSs were generated using single-trait PRS modeling methods, with summary statistics primarily derived from the BBJ, except for WC, which utilized data from the TWB. To assess the transferability of these PRSs from the Japanese and Chinese training populations to the Korean target population, we estimated SNP-based heritability and pairwise genetic correlations within and across the EAS populations using LDSC. PRS validation was performed using the same criteria as those for the single-trait models, excluding the AUC during the initial selection.

For comparative evaluation, four PRS models were examined: two single-trait PRSs for BMI (Model 1, M1) and WC (Model 2, M2), two multiple-trait models for obesity-related indicators only (Model 3, M3), and obesity-related indicators combined with metabolic traits (Model 4, M4). The predictive performance was assessed using AIC and AUC. Model comparisons were performed using the DeLong test [65].

### Association analyses of PRSs on obesity and obesity-related diseases

We evaluated the associations between the PRSs and obesity and obesity-related diseases using logistic regression models. Four PRSs, derived from two distinct modeling strategies (single-trait and multiple-trait approaches), were analyzed. In addition, we implemented the PRSsum method [66], in which multiple standardized PRSs were aggregated with equal weights to create composite scores. Two types of PRSsum were constructed: PRSsum1, composed solely of obesity-related indicators, and PRSsum2, which included both obesity-related indicators and metabolic health traits. This approach was intended to assess whether these composite indices could more effectively identify individuals at elevated risk of obesity and related conditions.

For each PRS model, individuals were stratified into three risk categories comprising of L (bottom 10%), M (middle 80%), and H (top 10%), corresponding to the low, medium, and high risk in PRS distribution. In disease risk associations, we focused on comparing the H group, presumed to carry an elevated genetic risk, with the combined L and M groups to examine their discriminatory capacity.

### Interaction and mediation analyses of the genetic risk for obesity on obesity-related diseases

To investigate how genetic predisposition to obesity influences related comorbidities, we conducted both interaction and mediation analyses. These analyses focused on T2D and CVD, for which summary statistics from the BBJ were publicly available. The validation procedures for PRS construction and performance followed the same protocols described in the previous sections.

For the interaction analyses, two logistic regression models were applied, with the following covariates: age, sex, the top five PCs, obesity status, obesity PRSs (including single-trait PRSs for BMI and WC, and multiple-trait PRSsum for PRSsum1 and PRSsum2), and the target disease PRS. The models differed in interaction terms: one was obesity status × disease PRS, and the other obesity PRS × disease PRS. Mediation analyses [67] were carried out to quantify the indirect effects of obesity between genetic risk and disease outcomes. The model framework specified obesity PRS as the independent variable (X), obesity status as the mediator (M), and obesity-related disease as the dependent variable (Y). This framework paralleled the four PRSs used in the interaction analyses. Mediation was tested in three sequential models: (1) disease outcome ∼ age + sex + obesity PRS, (2) obesity ∼ age + sex + obesity PRS, and (3) disease outcome ∼ age + sex + obesity PRS + obesity. We performed two versions of the analysis, with and without adjustment for disease PRS in the first and third models. All mediation analyses were conducted using the mediation package (v4.5.0) [68] in R.

## Ethical statement

This research was designed in accordance with the principles of the Declaration of Helsinki and conducted after approval of the institutional review board (IRB) of Seoul National University (Approval No. E2303/004-012).

## Supplementary Material

qzaf102_Supplementary_Data

## Data Availability

The GWAS summary statistics generated in this study from the discovery dataset, including BMI, WC, BMI25, BMI30, WC1, and WC2, are publicly available in the GWAS Catalog (GWAS Catalog: GCST90566412–GCST90566417).

## CRediT author statement

**Jinyeon Jo:** Conceptualization, Data curation, Formal analysis, Investigation, Methodology, Validation, Visualization, Writing – original draft, Writing – review & editing. **Nayoung Ha:** Writing – review & editing. **Yunmi Ji:** Writing – review & editing. **Ahra Do:** Data curation. **Je Hyun Seo:** Resources, Writing – review & editing. **Bumjo Oh:** Resources, Writing – review & editing. **Sungkyoung Choi:** Writing – review & editing. **Eun Kyung Choe:** Resources, Writing – review & editing. **Woojoo Lee:** Supervision, Project administration, Writing – review & editing. **Jang Won Son:** Conceptualization, Funding acquisition, Investigation, Project administration, Supervision, Writing – review & editing. **Sungho Won:** Conceptualization, Funding acquisition, Methodology, Project administration, Resources, Supervision, Writing – review & editing. All authors have read and approved the final manuscript.
