# Supplementary material for: Association of Multiple-trait Polygenic Risk Score with Obesity and Cardiometabolic Diseases in Korean Population
Source: Genomics Proteomics Bioinformatics. 2025 Nov 9;23(5):qzaf102. doi: 10.1093/gpbjnl/qzaf102 (PMC12975335; doi:10.1093/gpbjnl/qzaf102)
Supplement: qzaf102_Supplementary_Data [file qzaf102_supplementary_data.zip › 13-Feb-2026_113933_qzaf102_Supplementary_Data_TableS12Revised.zip!/qzaf102_Supplementary_Data_TableS12Revised/Table S13.docx]

**Table S13 Validation results of single trait PRS analyses for obesity**

| **Methods** | **General obesity** | | | | | **Abdominal obesity** | | | | |
| --- | --- | --- | --- | --- | --- | --- | --- | --- | --- | --- |
|  | **Cor.** | ***P* value** | **AIC** | **AUC** | | **Cor.** | ***P* value** | **AIC** | **AUC** | |
|  |  |  |  | **BMI25** | **BMI30** |  |  |  | **WC1** | **WC2** |
| CT | 0.138 | 1.91×10^-67^ | 31434.34 | 0.620 | 0.605 | 0.076 | 5.47×10^-24^ | 56565.70 | 0.614 | 0.572 |
| LDpred: infinitesimal | 0.221 | 5.52×10^-173^ | 30948.80 | 0.645 | 0.672 | 0.128 | 8.93×10^-75^ | 56332.75 | 0.631 | 0.619 |
| LDpred: grid1 ($\rho$ = 1%) | 0.227 | 3.25×10^-185^ | 30892.49 | 0.648 | 0.683 | 0.132 | 1.75×10^-77^ | 56320.30 | 0.632 | 0.629 |
| LDpred: grid2 ($\rho$ = 3%) | 0.238 | 2.10×10^-202^ | 30813.35 | 0.652 | 0.687 | 0.140 | 1.24×10^-86^ | 56278.24 | 0.634 | 0.630 |
| LDpred: grid3 ($\rho$ = 10%) | 0.235 | 2.17×10^-196^ | 30841.04 | 0.650 | 0.683 | 0.135 | 1.99×10^-82^ | 56297.57 | 0.633 | 0.625 |
| LDpred: grid4 ($\rho$ = 30%) | 0.226 | 1.94×10^-181^ | 30909.87 | 0.646 | 0.676 | 0.130 | 6.78×10^-77^ | 56323.00 | 0.631 | 0.621 |
| LDpred: grid5 ($\rho$ = 100%) | 0.220 | 6.84×10^-172^ | 30953.83 | 0.644 | 0.671 | 0.128 | 9.38×10^-75^ | 56332.85 | 0.631 | 0.619 |
| **LDpred: auto** | **0.241** | **4.43×10^-207^** | **30791.81** | **0.653** | **0.687** | **0.140** | **2.04×10^-87^** | **56274.63** | **0.634** | **0.633** |
| lassosum | 0.236 | 8.82×10^-197^ | 30839.23 | 0.650 | 0.682 | 0.137 | 3.41×10^-85^ | 56284.85 | 0.633 | 0.626 |
| PRS-CS | 0.225 | 5.66×10^-181^ | 30912.02 | 0.648 | 0.675 | 0.128 | 2.62×10^-76^ | 56325.70 | 0.630 | 0.622 |

*Note*: Cor, Pearson correlation between BMI and the PRS; *P* value, *P* value for PRS association; AIC, Akaike Information Criterion from linear models for BMI, adjusted for age, sex, PRS, and the first five principal components; AUC, mean area under the receiver operating characteristic curve obtained from 10-fold cross-validated logistic regression models for obesity outcomes, adjusted for age, sex, PRS, and the first five principal components.
