# Supplementary material for: Association of Multiple-trait Polygenic Risk Score with Obesity and Cardiometabolic Diseases in Korean Population
Source: Genomics Proteomics Bioinformatics. 2025 Nov 9;23(5):qzaf102. doi: 10.1093/gpbjnl/qzaf102 (PMC12975335; doi:10.1093/gpbjnl/qzaf102)
Supplement: qzaf102_Supplementary_Data [file qzaf102_supplementary_data.zip › 13-Feb-2026_113933_qzaf102_Supplementary_Data_TableS12Revised.zip!/qzaf102_Supplementary_Data_TableS12Revised/Supplementary material captions.docx]

**Supplementary captions**

**Figure S1 Manhattan plots constructed from GWAS results for obesity in the REP1_Kor_ replication dataset**

Circular and standard Manhattan plots are shown for four obesity phenotypes: BMI25 (BMI ≥ 25 kg/m²), BMI30 (≥ 30 kg/m²), WC1 (WC ≥ 85 cm for females / 90 cm for males), and WC2 (≥ 95 cm for females / 100 cm for males). The red dashed line indicates the genome-wide significance threshold. **A.** Circular plots arranged from inner to outer rings in the order of BMI25, BMI30, WC1, and WC2. **B.** Corresponding standard Manhattan plots. GWAS, genome-wide association study; PRS, polygenic risk score; BMI, body mass index; WC, waist circumference.

**Figure S2 Manhattan plots constructed from GWAS results for obesity in the REP2_Chi_ replication dataset**

Circular and standard Manhattan plots are shown for four obesity phenotypes: BMI25 (BMI ≥ 25 kg/m²), BMI30 (≥ 30 kg/m²), WC1 (WC ≥ 85 cm for females / 90 cm for males), and WC2 (≥ 95 cm for females / 100 cm for males). The red dashed line indicates the genome-wide significance threshold. **A.** Circular plots arranged from inner to outer rings in the order of BMI25, BMI30, WC1, and WC2. **B.** Corresponding standard Manhattan plots.

**Figure S3 Manhattan plots constructed from GWAS results for obesity in the REP3_NHW_ replication dataset**

Circular and standard Manhattan plots are shown for four obesity phenotypes: BMI30 (BMI ≥ 30 kg/m²), BMI40 (≥ 40 kg/m²), WC1 (WC ≥ 85 cm for females / 90 cm for males), and WC2 (≥ 95 cm for females / 100 cm for males). The red dashed line indicates the genome-wide significance threshold. **A.** Circular plots arranged from inner to outer rings in the order of BMI30, BMI40, WC1, and WC2. **B.** Corresponding standard Manhattan plots.

**Figure S4 Comparison of GWAS results for BMI and general obesity in the discovery dataset**

This figure compares genetic associations between continuous obesity-related indicators: BMI and dichotomous obesity definitions: BMI25 (BMI ≥ 25 kg/m²), BMI30 (≥ 30 kg/m²). **A.** QQ plots. **B.** Circular Manhattan plots for BMI and two severity levels of general obesity. **C.** Corresponding standard Manhattan plots. QQ, quantile–quantile.

**Figure S5 Comparison of GWAS results for WC and abdominal obesity in the discovery dataset**

This figure compares genetic associations between continuous obesity-related indicators: WC and dichotomous obesity definitions: WC1 (WC ≥ 85 cm for females / 90 cm for males) / WC2 (≥ 95 cm for females / 100 cm for males). **A.** QQ plots. **B.** Circular Manhattan plots for WC and two severity levels of abdominal obesity. **C.** Corresponding standard Manhattan plots.

**Figure S6 Manhattan plots from GWAS of obesity-related diseases in the discovery dataset**

Circular and standard Manhattan plots are shown for five obesity-related disease phenotypes: HT, T2D, CVD, HL, and FL. The red dashed line indicates the genome-wide significance threshold. **A.** Circular plots arranged from inner to outer rings. **B.** Corresponding standard Manhattan plots. HT, hypertension; T2D, type 2 diabetes; CVD, cardiovascular disease; HL, hyperlipidemia; FL, fatty liver.

**Figure S7 Manhattan plots from GWAS of obesity-related diseases in the REP3_NHW_ replication dataset**

Circular and standard Manhattan plots are shown for five obesity-related disease phenotypes: HT, T2D, CVD, HL, and FL. The red dashed line indicates the genome-wide significance threshold. **A.** Circular plots arranged from inner to outer rings. **B.** Corresponding standard Manhattan plots.

**Figure S8 Local genetic correlation between general obesity and obesity-related diseases**

Local genetic correlation plots of abdominal obesity (WC1, WC ≥ 85 cm for females / 90 cm for males) are shown for five obesity-related disease phenotypes: HT, T2D, CVD, HL, and FL **A.** Plots from the discovery dataset, with the red line indicating signals that meet the Bonferroni-corrected significance threshold. **B.** Plots from the REP3_NHW_ replication dataset, with the blue line indicating signals that surpass the same Bonferroni threshold.

**Figure S9 Local genetic correlation between abdominal obesity and obesity-related diseases**

Local genetic correlation plots of abdominal obesity (WC1, waist circumference ≥ 85 cm for females / 90 cm for males) are shown for five obesity-related disease phenotypes: HT, T2D, CVD, HL, and FL. **A.** Plots from the discovery dataset, with the red line indicating signals that meet the Bonferroni-corrected significance threshold. **B.** Plots from the REP3_NHW_ replication dataset, with the blue line indicating signals that surpass the same Bonferroni threshold.

**Table S1**  **Baseline characteristics according to the incident obesity on REP1_Kor_ replication dataset**

**Table S2 Baseline characteristics according to the incident obesity on REP2_Chi_ replication dataset**

**Table S3**  **Baseline characteristics according to the incident obesity on REP3_NHW_ replication dataset**

**Table S4 Significant SNPs identified from GWASs of obesity-related indicators**

**Table S5 Significant SNPs identified from GWASs of obesity-related diseases**

**Table S6**  **Genomic inflation, SNP heritability, and genetic correlations for obesity-related indicators and obesity-related diseases**

**Table S7 Significant genetic regions identified by local genetic correlations between obesity and obesity-related diseases**

**Table S8 Significant genes identified through gene-based analyses of obesity-related indicators and obesity-related diseases**

**Table S9 Summary for gene-set analyses results for obesity-related diseases**

**Table S10 Significant gene-sets identified through gene-set analyses of obesity-related diseases**

**Table S11 Distribution of obesity and obesity-related diseases in samples used for PRS analyses**

**Table S12 Heritability analyses and genetic correlations** **for PRS target traits in EAS populations**

**Table S13 Validation results of single-trait PRS analyses for obesity**

**Table S14 Validation results of multiple-trait PRS analyses**

**Table S15 Model performance improvement using single-trait and multiple-trait PRS analyses**

**Table S16 Validation results for obesity-related diseases T2D and CVD**

**Table S17 Interaction and mediation effects of obesity on obesity-related diseases T2D and CVD**
