# Supplementary material for: Association of Multiple-trait Polygenic Risk Score with Obesity and Cardiometabolic Diseases in Korean Population
Source: Genomics Proteomics Bioinformatics. 2025 Nov 9;23(5):qzaf102. doi: 10.1093/gpbjnl/qzaf102 (PMC12975335; doi:10.1093/gpbjnl/qzaf102)
Supplement: qzaf102_Supplementary_Data [file qzaf102_supplementary_data.zip › 13-Feb-2026_113933_qzaf102_Supplementary_Data_TableS12Revised.zip!/qzaf102_Supplementary_Data_TableS12Revised/Table S1.docx]

**Table S1 Baseline characteristics according to the incident obesity on REP1_Kor_**

**A. Baseline characteristics according to the incident general obesity**

| **Variables** | **Total**  **(*n* = 7089)** | **BMI25 (prevalence = 39.70%)** | | | **BMI30 (prevalence = 4.57%)** | | | **Missing data**  ***n* (%)** |
| --- | --- | --- | --- | --- | --- | --- | --- | --- |
|  |  | **Non-obese**  **(*n* = 4275)** | **Obese**  **(*n* = 2814)** | ***P* value** | **Non-obese**  **(*n* = 6765)** | **Obese**  **(*n* = 324)** | ***P* value** |  |
| **Age (years)** | 54.55 ± 9.06 | 54.30 ± 9.21 | 54.92 ± 8.82 | 0.004 | 54.52 ± 9.09 | 55.21 ± 8.49 | 0.151 | 0 (0%) |
| **Sex**, *n* (%) |  |  |  | < 0.001 |  |  | < 0.001 | 0 (0%) |
| Female | 3850 (54.31%) | 2279 (59.19%) | 1571 (40.81%) |  | 3615 (93.90%) | 235 (6.10%) |  |  |
| Male | 3239 (45.69%) | 1996 (61.62%) | 1243 (38.38%) |  | 3150 (97.25%) | 89 (2.75%) |  |  |
| **BMI** (kg/m^2^) | 24.37 ± 3.13 | 22.39 ± 1.82 | 27.38 ± 2.14 | < 0.001 | 24.01 ± 2.68 | 31.94 ± 1.91 | < 0.001 | 0 (0%) |
| **WC** (cm) |  |  |  | < 0.001 |  |  | < 0.001 | 29 (0.41%) |
| Female | 81.66 ± 9.34 | 76.79 ± 7.03 | 88.15 ± 7.91 |  | 80.48 ± 8.46 | 96.12 ± 9.04 |  |  |
| Male | 85.54 ± 7.84 | 81.61 ± 6.29 | 91.86 ± 5.67 |  | 85.11 ± 7.47 | 100.98 ± 4.86 |  |  |
| **Body fat ratio** (%) | 27.44 ± 7.30 | 24.23 ± 6.25 | 31.66 ± 6.36 | < 0.001 | 26.82 ± 6.90 | 38.14 ± 5.54 | < 0.001 | 4433 (62.53%) |
| **Abdominal fat ratio** (%) | 0.91 ± 0.05 | 0.88 ± 0.04 | 0.94 ± 0.04 | < 0.001 | 0.90 ± 0.04 | 1.00 ± 0.05 | < 0.001 | 4433 (62.53%) |
| **SBP** (mm Hg) | 124.64 ± 17.90 | 122.46 ± 17.74 | 127.96 ± 17.64 | < 0.001 | 124.33 ± 17.78 | 131.18 ± 19.12 | < 0.001 | 2 (0.03%) |
| **DBP** (mm Hg) | 79.60 ± 11.25 | 78.03 ± 11.15 | 81.99 ± 10.99 | < 0.001 | 79.37 ± 11.22 | 84.46 ± 10.90 | < 0.001 | 2 (0.03%) |
| **Fasting plasma glucose** (mg/dl) | 97.21 ± 33.33 | 94.78 ± 29.98 | 100.91 ± 37.57 | < 0.001 | 96.90 ± 33.29 | 103.56 ± 33.49 | 0.001 | 68 (0.96%) |
| **HbA1c** (%) | 5.86 ± 0.99 | 5.76 ± 0.95 | 5.98 ± 1.03 | < 0.001 | 5.84 ± 0.99 | 6.16 ± 1.03 | < 0.001 | 4434 (62.55%) |
| **Triglyceride** (mg/dl) | 151.38 ± 110.86 | 137.04 ± 104.65 | 173.16 ± 116.36 | < 0.001 | 149.68 ± 109.35 | 186.93 ± 134.01 | < 0.001 | 4 (0.06%) |
| **HDL** (mg/dl) |  |  |  | < 0.001 |  |  | < 0.001 | 0 (0%) |
| Female | 49.93 ± 13.01 | 52.61 ± 13.53 | 47.00 ± 11.79 |  | 50.66 ± 13.17 | 45.13 ± 11.42 |  |  |
| Male | 46.88 ± 12.22 | 48.73 ± 12.72 | 43.90 ± 10.74 |  | 47.02 ± 12.22 | 41.78 ± 10.99 |  |  |
| **Hypertension**, case *n* (%) | 1488 (21.00%) | 660 (15.44%) | 828 (29.43%) | < 0.001 | 1359 (20.09%) | 129 (39.81%) | < 0.001 | 2 (0.03%) |
| **T2D**, case *n* (%) | 973 (14.59%) | 472 (11.63%) | 501 (19.18%) | < 0.001 | 901 (14.15%) | 72 (23.76%) | < 0.001 | 420 (5.92%) |
| **CVD**, case *n* (%) | 249 (3.51%) | 134 (3.14%) | 115 (4.09%) | < 0.001 | 230 (3.40%) | 19 (5.86%) | < 0.001 | 2 (0.03%) |
| **HL**, case *n* (%) | 318 (4.49%) | 157 (3.67%) | 161 (5.72%) | < 0.001 | 296 (4.38%) | 22 (6.79%) | < 0.001 | 2 (0.03%) |
| **FL**, case *n* (%) | 290 (6.54%) | 114 (4.12%) | 176 (10.56%) | < 0.001 | 264 (6.21%) | 26 (14.44%) | < 0.001 | 2657 (37.48%) |

**B. Baseline characteristics according to the incident abdominal obesity**

| **Variables** | **Total**  **(*n* = 7697)** | **WC1 (Prevalence = 33.19%)** | | | **WC2 (Prevalence = 6.51%)** | | | **Missing data**  ***n* (%)** |
| --- | --- | --- | --- | --- | --- | --- | --- | --- |
|  |  | **Non-obese**  **(*n* = 5142)** | **Obese**  **(*n* = 2555)** | ***P* value** | **Non-obese**  **(*n* = 7196)** | **Obese**  **(*n* = 501)** | ***P* value** |  |
| **Age (years)** | 54.42 ± 9.10 | 53.42 ± 9.10 | 57.12 ± 8.50 | < 0.001 | 54.41 ± 9.06 | 58.17 ± 8.48 | < 0.001 | 0 (0%) |
| **Sex**, *n* (%) |  |  |  | < 0.001 |  |  | < 0.001 | 0 (0%) |
| Female | 4145 (63.55%) | 2634 (63.55%) | 1511 (36.45%) |  | 3767 (90.88%) | 378 (9.12%) |  |  |
| Male | 3552 (46.15%) | 2508 (70.61%) | 1044 (29.39%) |  | 3429 (96.54%) | 123 (3.46%) |  |  |
| **BMI** (kg/m^2^) | 24.37 ± 3.12 | 23.05 ± 2.37 | 27.04 ± 2.73 | < 0.001 | 24.01 ± 2.79 | 29.65 ± 2.98 | < 0.001 | 637 (8.28%) |
| **WC** (cm) |  |  |  | < 0.001 |  |  | < 0.001 | 0 (0%) |
| Female | 81.66 ± 9.34 | 76.03 ± 5.70 | 91.48 ± 5.58 |  | 79.89 ± 7.72 | 99.34 ± 4.32 |  |  |
| Male | 85.39 ± 7.88 | 81.57 ± 5.59 | 94.58 ± 4.06 |  | 84.77 ± 7.25 | 102.90 ± 3.06 |  |  |
| **Body fat ratio** (%) | 27.44 ± 7.30 | 25.25 ± 6.68 | 32.51 ± 6.04 | < 0.001 | 26.78 ± 6.99 | 36.29 ± 5.36 | < 0.001 | 5043 (65.52%) |
| **Abdominal fat ratio** (%) | 0.91 ± 0.05 | 0.89 ± 0.04 | 0.95 ± 0.05 | < 0.001 | 0.90 ± 0.05 | 0.97 ± 0.05 | < 0.001 | 5043 (65.52%) |
| **SBP** (mm Hg) | 124.85 ± 18.14 | 122.43 ± 17.83 | 129.73 ± 17.77 | < 0.001 | 124.35 ± 18.03 | 132.04 ± 18.08 | < 0.001 | 2 (0.03%) |
| **DBP** (mm Hg) | 79.82 ± 11.30 | 78.42 ± 11.22 | 82.66 ± 10.91 | < 0.001 | 79.53 ± 11.28 | 84.02 ± 10.64 | < 0.001 | 2 (0.03%) |
| **Fasting plasma glucose** (mg/dl) | 96.39 ± 32.76 | 93.77 ± 31.62 | 101.74 ± 34.36 | < 0.001 | 95.88 ± 32.19 | 103.91 ± 39.54 | < 0.001 | 126 (1.64%) |
| **HbA1c** (%) | 5.87 ± 1.01 | 5.76 ±0.94 | 6.11 ± 1.11 | < 0.001 | 5.85 ± 1.00 | 6.17 ± 1.08 | < 0.001 | 4411 (57.31%) |
| **Triglyceride** (mg/dl) | 152.82 ± 110.57 | 139.59 ± 103.92 | 179.44 ± 118.49 | < 0.001 | 150.51 ± 110.50 | 186.06 ± 106.33 | < 0.001 | 6 (0.08%) |
| **HDL** (mg/dl) |  |  |  | < 0.001 |  |  | < 0.001 | 1 (0.01%) |
| Female | 49.93 ± 13.01 | 52.09 ± 13.51 | 46.26 ± 11.20 |  | 50.53 ± 13.15 | 44.35 ± 10.13 |  |  |
| Male | 46.72 ± 12.18 | 48.11 ± 12.48 | 43.36 ± 10.75 |  | 46.88 ± 12.21 | 41.89 ± 10.60 |  |  |
| **Hypertension**, case *n* (%) | 1613 (20.96%) | 785 (15.27%) | 828 (32.41%) | < 0.001 | 1613 (20.96%) | 1402 (19.49%) | < 0.001 | 1 (0.01%) |
| **T2D**, case *n* (%) | 1067 (14.67%) | 536 (10.89%) | 531 (22.62%) | < 0.001 | 1067 (14.67%) | 955 (14.00%) | < 0.001 | 426 (5.53%) |
| **CVD**, case *n* (%) | 288 (3.74%) | 157 (3.05%) | 131 (5.13%) | < 0.001 | 288 (3.74%) | 253 (3.52%) | < 0.001 | 1 (0.01%) |
| **HL**, case *n* (%) | 324 (4.21%) | 197 (3.83%) | 127 (4.97%) | < 0.001 | 324 (4.21%) | 294 (4.09%) | < 0.001 | 1 (0.01%) |
| **FL**, case *n* (%) | 289 (6.55%) | 140 (4.86%) | 149 (9.74%) | < 0.001 | 289 (6.55%) | 260 (6.27%) | < 0.001 | 3288 (42.72%) |

*Note*: Kor, Korean; BMI, body mass index; WC, waist circumference; BMI25, BMI ≥ 25 kg/m²; BMI30, BMI ≥ 30 kg/m²; SBP, systolic blood pressure; DBP, diastolic blood pressure; HDL, high-density lipoprotein; T2D, type 2 diabetes; CVD, cardiovascular disease; HL, hyperlipidemia; FL, fatty liver.
