# Supplementary material for: Association of Multiple-trait Polygenic Risk Score with Obesity and Cardiometabolic Diseases in Korean Population
Source: Genomics Proteomics Bioinformatics. 2025 Nov 9;23(5):qzaf102. doi: 10.1093/gpbjnl/qzaf102 (PMC12975335; doi:10.1093/gpbjnl/qzaf102)
Supplement: qzaf102_Supplementary_Data [file qzaf102_supplementary_data.zip › 13-Feb-2026_113933_qzaf102_Supplementary_Data_TableS12Revised.zip!/qzaf102_Supplementary_Data_TableS12Revised/Table S2.docx]

**Table S2 Baseline characteristics according to the incident obesity on REP2_Chi_**

**A. Baseline characteristics according to the incident general obesity**

| **Variables** |  | **BMI25 (prevalence = 35.88%)** | | | **BMI30 (prevalence = 5.35%)** | | |  |
| --- | --- | --- | --- | --- | --- | --- | --- | --- |
|  | **Total**  **(*n* = 1494)** | **Non-obese**  **(*n* = 958)** | **Obese**  **(*n* = 536)** | ***P* value** | **Non-obese**  **(*n* = 1414)** | **Obese**  **(*n* = 80)** | ***P* value** | **Missing data**  ***n* (%)** |
| **Age (years)** | 52.44 ± 7.65 | 52.09 ± 7.58 | 53.05 ± 7.74 | 0.020 | 52.48 ± 7.66 | 51.73 ± 7.59 | 0.393 | 0 (0%) |
| **Sex**, *n* (%) |  |  |  | < 0.001 |  |  | < 0.001 | 0 (0%) |
| Female | 933 (62.45%) | 670 (71.81%) | 263 (28.19%) |  | 895 (95.93%) | 38 (4.07%) |  |  |
| Male | 561 (37.55%) | 288 (51.34%) | 273 (48.66%) |  | 519 (92.51%) | 42 (7.48%) |  |  |
| **BMI** (kg/m^2^) | 24.11 ± 3.44 | 22.09 ± 1.84 | 27.72 ± 2.57 | < 0.001 | 23.64 ± 2.82 | 32.45 ± 2.73 | < 0.001 | 0 (0%) |
| **WC** (cm) |  |  |  | < 0.001 |  |  | < 0.001 | 0 (0%) |
| Female | 76.30 ± 8.86 | 72.60 ± 6.15 | 85.56 ± 7.69 |  | 75.43 ± 7.81 | 95.74 ± 9.03 |  |  |
| Male | 87.46 ± 8.88 | 81.98 ± 6.25 | 93.25 ± 7.46 |  | 86.12 ± 7.60 | 104.07 ± 6.38 |  |  |
| **Body fat ratio** (%) | 26.75 ± 7.14 | 24.83 ± 6.29 | 30.18 ± 7.28 | < 0.001 | 26.29 ± 6.85 | 34.79 ± 7.37 | < 0.001 | 18 (1.20%) |
| **Abdominal fat ratio** (%) | 0.46 ± 0.10 | 0.44 ± 0.09 | 0.52 ± 0.08 | < 0.001 | 0.45 ± 0.09 | 0.58 ± 0.02 | < 0.001 | 1365 (91.37%) |
| **SBP** (mm Hg) | 131.51 ± 19.06 | 128.56 ± 19.26 | 136.72 ± 17.55 | < 0.001 | 131.20 ± 19.27 | 136.82 ± 14.11 | 0.001 | 60 (4.02%) |
| **DBP** (mm Hg) | 81.55 ± 10.78 | 79.57 ± 10.63 | 85.05 ± 10.15 | < 0.001 | 81.32 ± 10.84 | 85.47 ± 8.89 | < 0.001 | 60 (4.02%) |
| **Fasting plasma glucose** (mg/dl) | 5.11 ± 0.99 | 5.06 ± 1.03 | 5.18 ± 0.92 | 0.035 | 5.09 ± 0.94 | 5.45 ± 1.64 | 0.087 | 203 (13.59%) |
| **HbA1c** (%) | 37.15 ± 5.96 | 36.17 ± 5.50 | 38.88 ± 6.34 | < 0.001 | 36.94 ± 5.83 | 40.88 ± 7.04 | < 0.001 | 70 (4.69%) |
| **Triglyceride** (mg/dl) | 1.76 ± 1.11 | 1.55 ± 0.98 | 2.12 ± 1.22 | < 0.001 | 1.74 ± 1.10 | 2.16 ± 1.08 | 0.001 | 78 (5.22%) |
| **HDL** (mg/dl) |  |  |  | < 0.001 |  |  | < 0.001 | 203 (13.59%) |
| Female | 1.59 ± 0.37 | 1.67 ± 0.38 | 1.41 ± 0.29 |  | 1.60 ± 0.37 | 1.32 ± 0.22 |  |  |
| Male | 1.25 ± 0.29 | 1.33 ± 0.31 | 1.17 ± 0.24 |  | 1.26 ± 0.29 | 1.11 ± 0.19 |  |  |
| **Hypertension**, case *n* (%) | 599 (40.31%) | 310 (32.53%) | 289 (54.22%) | < 0.001 | 546 (38.83%) | 53 (66.25%) | < 0.001 | 8 (0.54%) |
| **T2D**, case *n* (%) | 115 (9.26%) | 36 (4.57%) | 79 (17.40%) | < 0.001 | 97 (8.23%) | 18 (28.57%) | < 0.001 | 252 (16.87%) |
| **CVD**, case *n* (%) | 68 (6.13%) | 29 (4.17%) | 39 (9.42%) | < 0.001 | 57 (5.50%) | 11 (15.28%) | 0.003 | 385 (25.77%) |
| **HL**, case *n* (%) | 102 (9.19%) | 39 (5.61%) | 63 (15.18%) | < 0.001 | 88 (8.48%) | 14 (19.44%) | 0.004 | 384 (25.70%) |
| **FL**, case *n* (%) | 2 (0.18%) | 1 (0.14%) | 1 (0.24%) | 1.000 | 2 (0.19%) | 0 (0.00%) | 1.000 | 384 (25.70%) |

**B. Baseline characteristics according to the incident abdominal obesity**

| **Variables** |  | **WC1 (prevalence = 25.53%)** | | | **WC2 (prevalence = 5.28%)** | | |  |
| --- | --- | --- | --- | --- | --- | --- | --- | --- |
|  | **Total**  **(*n* = 1496)** | **Non-obese**  **(*n* = 1114)** | **Obese**  **(*n* = 382)** | ***P* value** | **Non-obese**  **(*n* = 1417)** | **Obese**  **(*n* = 79)** | ***P* value** | **Missing data**  ***n* (%)** |
| **Age (years)** | 52.45 ± 7.66 | 51.98 ± 7.54 | 53.82 ± 7.85 | < 0.001 | 52.41 ± 7.62 | 53.19 ± 8.32 | 0.379 | 0 (0%) |
| **Sex**, *n* (%) |  |  |  | < 0.001 |  |  | < 0.001 | 0 (0%) |
| Female | 935 (62.50%) | 774 (82.78%) | 161 (17.22%) |  | 909 (92.22%) | 26 (2.78%) |  |  |
| Male | 561 (37.50%) | 340 (60.61%) | 221 (39.39%) |  | 508 (90.55%) | 53 (9.45%) |  |  |
| **BMI** (kg/m^2^) | 24.09 ± 3.44 | 22.77 ± 2.37 | 28.04 ± 3.07 | < 0.001 | 23.70 ± 2.93 | 31.50 ± 3.59 | < 0.001 | 2 (0.13%) |
| **WC** (cm) |  |  |  | < 0.001 |  |  | < 0.001 | 0 (0%) |
| Female | 76.30 ± 8.86 | 73.33 ± 6.04 | 90.54 ± 5.91 |  | 75.60 ± 7.84 | 100.77 ± 7.54 |  |  |
| Male | 87.46 ± 8.88 | 81.85 ± 5.26 | 96.09 ± 5.90 |  | 85.65 ± 7.06 | 104.84 ± 4.79 |  |  |
| **Body fat ratio** (%) | 26.75 ± 7.14 | 25.62 ± 6.68 | 30.03 ± 7.42 | 0.001 | 26.45 ± 6.98 | 32.33 ± 7.78 | < 0.001 | 20 (1.34%) |
| **Abdominal fat ratio** (%) | 0.46 ± 0.10 | 0.44 ± 0.09 | 0.51 ± 0.08 | < 0.001 | 0.45 ± 0.09 | 0.55 ± 0.08 | 0.016 | 1367 (91.38%) |
| **SBP** (mm Hg) | 131.53 ± 19.06 | 129.23 ± 19.30 | 138.21 ± 16.66 | < 0.001 | 131.09 ± 19.20 | 139.29 ± 4.40 | < 0.001 | 60 (4.01%) |
| **DBP** (mm Hg) | 81.52 ± 10.79 | 80.01 ± 10.67 | 85.91 ± 9.93 | < 0.001 | 81.26 ± 10.81 | 86.22 ± 9.28 | < 0.001 | 60 (4.01%) |
| **Fasting plasma glucose** (mg/dl) | 5.11 ± 1.01 | 5.06 ± 0.98 | 5.27 ± 1.07 | 0.002 | 5.08 ± 0.94 | 5.62 ± 1.72 | 0.013 | 204 (13.64%) |
| **HbA1c** (%) | 37.18 ± 6.05 | 36.34 ± 5.74 | 39.67 ± 6.26 | < 0.001 | 36.94 ± 5.81 | 41.61 ± 8.36 | < 0.001 | 70 (4.68%) |
| **Triglyceride** (mg/dl) | 1.76 ± 1.11 | 1.60 ± 1.00 | 2.23 ± 1.27 | < 0.001 | 1.73 ± 1.08 | 2.34 ± 1.36 | < 0.001 | 79 (5.28%) |
| **HDL** (mg/dl) |  |  |  | < 0.001 |  |  | < 0.001 | 204 (13.64%) |
| Female | 1.59 ± 0.37 | 1.64 ± 0.38 | 1.36 ± 0.26 |  | 1.60 ± 0.37 | 1.36 ± 0.26 |  |  |
| Male | 1.25 ± 0.29 | 1.31 ± 0.30 | 1.17 ± 0.23 |  | 1.26 ± 0.29 | 1.15 ± 0.23 |  |  |
| **Hypertension**, case n (%) | 601 (40.39%) | 373 (33.73%) | 228 (59.69%) | < 0.001 | 545 (38.68%) | 56 (70.89%) | < 0.001 | 8 (0.53%) |
| **T2D**, case *n* (%) | 116 (9.33%) | 54 (5.81%) | 62 (19.81%) | < 0.001 | 98 (8.31%) | 18 (28.12%) | < 0.001 | 253 (16.91%) |
| **CVD**, case *n* (%) | 68 (6.12%) | 39 (4.79%) | 29 (9.80%) | 0.003 | 58 (5.58%) | 10 (14.08%) | 0.009 | 385 (24.74%) |
| **HL**, case *n* (%) | 102 (9.17%) | 53 (6.50%) | 49 (16.50%) | < 0.001 | 89 (8.55%) | 13 (18.31%) | 0.011 | 384 (25.67%) |
| **FL**, case *n* (%) | 2 (0.18%) | 2 (0.25%) | 0 (0.00%) | 1.000 | 2 (0.19%) | 0 (0.00%) | 1.000 | 384 (25.67%) |

*Note*: Chi, Chinese.
