# Supplementary material for: Association of Multiple-trait Polygenic Risk Score with Obesity and Cardiometabolic Diseases in Korean Population
Source: Genomics Proteomics Bioinformatics. 2025 Nov 9;23(5):qzaf102. doi: 10.1093/gpbjnl/qzaf102 (PMC12975335; doi:10.1093/gpbjnl/qzaf102)
Supplement: qzaf102_Supplementary_Data [file qzaf102_supplementary_data.zip › 13-Feb-2026_113933_qzaf102_Supplementary_Data_TableS12Revised.zip!/qzaf102_Supplementary_Data_TableS12Revised/Table S3.docx]

**Table S3 Baseline characteristics according to the incident obesity on REP3_NHW_**

**A. Baseline characteristics according to the incident general obesity**

| **Variables** |  | **BMI30 (prevalence = 24.13%)** | | | | **BMI40 (prevalence = 1.87%)** | | | |  | |
| --- | --- | --- | --- | --- | --- | --- | --- | --- | --- | --- | --- |
|  | **Total**  **(*n* = 391,463)** | **Non-obese**  **(*n* = 297,015)** | **Obese**  **(*n* = 94,448)** | ***P* value** | **Non-obese**  **(*n* = 384,147)** | | **Obese**  **(*n* = 7316)** | ***P* value** | **Missing data**  ***n* (%)** | |  |
| **Age (years)** | 56.77 ± 7.99 | 56.66 ± 8.07 | 57.14 ± 7.75 | < 0.001 | 56.79 ± 8.00 | | 55.67 ± 7.58 | < 0.001 | 0 (0%) | |  |
| **Sex**, *n* (%) |  |  |  | < 0.001 |  | |  | < 0.001 | 0 (0%) | |  |
| Female | 211,227 (53.96%) | 162,713 (77.03%) | 48,514 (22.97%) |  | 206,314 (97.67%) | | 4913 (2.33%) |  |  | |  |
| Male | 180,236 (46.04%) | 134,302 (74.51%) | 45,934 (25.49%) |  | 177,833 (98.67%) | | 2403 (1.33%) |  |  | |  |
| **BMI** (kg/m^2^) | 27.39 ± 4.77 | 25.31 ± 2.71 | 33.92 ± 3.85 | < 0.001 | 27.07 ± 4.21 | | 43.75 ± 3.73 | < 0.001 | 0 (0%) | |  |
| **WC** (cm) |  |  |  | < 0.001 |  | |  | < 0.001 | 82 (0.02%) | |  |
| Female | 84.53 ± 12.50 | 79.77 ± 8.51 | 100.48 ± 10.29 |  | 83.75 ± 11.48 | | 117.00 ± 10.04 |  |  | |  |
| Male | 97.06 ± 11.35 | 92.56 ± 7.89 | 110.16 ± 9.57 |  | 96.58 ± 10.60 | | 131.87 ± 9.76 |  |  | |  |
| **Body fat ratio** (%) | 31.34 ± 8.52 | 29.19 ± 7.57 | 38.12 ± 7.75 | < 0.001 | 31.05 ± 8.29 | | 46.53 ± 6.15 | < 0.001 | 5906 (1.51%) | |  |
| **Abdominal fat ratio** (%) | 0.50 ± 0.11 | 0.47 ± 0.11 | 0.59 ± 0.08 | < 0.001 | 0.49 ± 0.11 | | 0.68 ± 0.06 | < 0.001 | 359,556 (91.85%) | |  |
| **SBP** (mm Hg) | 138.04 ± 18.62 | 136.73 ± 18.70 | 142.14 ± 17.77 | < 0.001 | 137.94 ± 18.62 | | 143.14 ± 17.75 | < 0.001 | 22,506 (5.75%) | |  |
| **DBP** (mm Hg) | 82.22 ± 10.12 | 81.06 ± 9.95 | 85.87 ± 9.77 | < 0.001 | 82.11 ± 10.09 | | 87.87 ± 10.10 | < 0.001 | 22,503 (5.75%) | |  |
| **Fasting plasma glucose** (mg/dl) | 5.12 ± 1.21 | 5.02 ± 1.00 | 5.41 ± 1.67 | < 0.001 | 5.10 ± 1.18 | | 5.84 ± 2.25 | < 0.001 | 50,117 (12.80%) | |  |
| **HbA1c** (%) | 35.95 ± 6.53 | 35.19 ± 5.42 | 38.33 ± 8.74 | < 0.001 | 35.83 ± 6.34 | | 41.81 ± 11.43 | < 0.001 | 18,275 (4.67%) | |  |
| **Triglyceride** (mg/dl) | 1.75 ± 1.02 | 1.62 ± 0.94 | 2.17 ± 1.16 | < 0.001 | 1.75 ± 1.02 | | 2.15 ± 1.08 | < 0.001 | 18,465 (4.72%) | |  |
| **HDL** (mg/dl) | 1.45 ± 0.38 |  |  | < 0.001 |  | |  | < 0.001 | 49,867 (12.74%) | |  |
| Female | 1.60 ± 0.38 | 1.66 ± 0.38 | 1.40 ± 0.31 |  | 1.61 ± 0.38 | | 1.29 ± 0.27 |  |  | |  |
| Male | 1.28 ± 0.31 | 1.33 ± 0.32 | 1.15 ± 0.26 |  | 1.29 ± 0.31 | | 1.07 ± 0.23 |  |  | |  |
| **Hypertension**, case *n* (%) | 205,226 (52.80%) | 139,934 (47.48%) | 65,292 (69.49%) | < 0.001 | 205,226 (52.80%) | | 199,389 (52.28%) | < 0.001 | 2792 (0.71%) | |  |
| **T2D**, case *n* (%) | 24,814 (7.57%) | 10,801 (4.36%) | 14,013 (17.54%) | < 0.001 | 24,814 (7.57%) | | 22,650 (7.05%) | < 0.001 | 63,829 (16.31%) | |  |
| **CVD**, case *n* (%) | 40,084 (12.46%) | 25,924 (10.80%) | 14,160 (17.29%) | < 0.001 | 40,084 (12.46%) | | 38,864 (12.33%) | < 0.001 | 69,657 (17.79%) | |  |
| **HL**, case *n* (%) | 40,426 (12.53%) | 25,230 (10.49%) | 15,196 (18.51%) | < 0.001 | 40,426 (12.53%) | | 39,014 (12.35%) | < 0.001 | 68,921 (17.61%) | |  |
| **FL**, case *n* (%) | 757 (0.23%) | 509 (0.21%) | 248 (0.30%) | < 0.001 | 757 (0.23%) | | 738 (0.23%) | 0.460 | 68,921 (17.61%) | |  |

**B. Baseline characteristics according to the incident abdominal obesity**

| **Variables** |  | **WC1 (prevalence = 58.37%)** | | | **WC2 (prevalence = 27.95%)** | | | |  |
| --- | --- | --- | --- | --- | --- | --- | --- | --- | --- |
|  | **Total**  **(*n* = 392,065)** | **Non-obese**  **(*n* = 163,226)** | **Obese**  **(*n* = 228,839)** | ***P* value** | | **Non-obese**  **(*n* = 282,466)** | **Obese**  **(*n* = 109,599)** | ***P* value** | **Missing data**  ***n* (%)** |
| **Age (years)** | 56.77 ± 7.99 | 55.71 ± 8.14 | 57.54 ± 7.80 | < 0.001 | | 56.36 ± 8.09 | 57.84 ± 7.65 | < 0.001 | 0 (0%) |
| **Sex**, *n* (%) |  |  |  | < 0.001 | |  |  | < 0.001 | 0 (0%) |
| Female | 211,491 (53.94%) | 117,332 (55.48%) | 94,159 (44.52%) |  | | 169,293 (80.05%) | 42,198 (19.95%) |  |  |
| Male | 180,574 (46.06%) | 45,894 (25.42%) | 134,680 (74.58%) |  | | 113,173 (62.67%) | 67,401 (37.33%) |  |  |
| **BMI** (kg/m^2^) | 27.38 ± 4.77 | 23.86 ± 2.49 | 29.90 ± 4.39 | < 0.001 | | 25.38 ± 3.05 | 32.57 ± 4.48 | < 0.001 | 684 (0.17%) |
| **WC** (cm) |  |  |  | < 0.001 | |  |  | < 0.001 | 0 (0%) |
| Female | 84.53 ± 12.50 | 75.61 ± 5.63 | 95.65 ± 9.42 |  | | 79.74 ± 7.93 | 103.77 ± 8.28 |  |  |
| Male | 97.06 ± 11.35 | 83.94 ± 4.50 | 101.53 ± 9.34 |  | | 90.23 ± 6.32 | 108.53 ± 8.27 |  |  |
| **Body fat ratio** (%) | 31.34 ± 8.52 | 28.78 ± 7.73 | 33.17 ± 8.58 | < 0.001 | | 29.70 ± 8.02 | 35.58 ± 8.29 | < 0.001 | 6488 (1.65%) |
| **Abdominal fat ratio** (%) | 0.50 ± 0.11 | 0.46 ± 0.11 | 0.53 ± 0.10 | < 0.001 | | 0.48 ± 0.11 | 0.57 ± 0.09 | < 0.001 | 360,142 (91.86%) |
| **SBP** (mm Hg) | 138.04 ± 18.62 | 133.68 ± 18.84 | 141.13 ± 17.83 | < 0.001 | | 136.30 ± 18.73 | 142.50 ± 17.59 | < 0.001 | 22,557 (5.75%) |
| **DBP** (mm Hg) | 82.22 ± 10.12 | 79.13 ± 9.70 | 84.41 ± 9.83 | < 0.001 | | 80.88 ± 9.90 | 85.66 ± 9.86 | < 0.001 | 22,554 (5.75%) |
| **Fasting plasma glucose** (mg/dl) | 5.12 ± 1.21 | 4.95 ± 0.85 | 5.24 ± 1.40 | < 0.001 | | 5.00 ± 0.95 | 5.41 ± 1.67 | < 0.001 | 50,199 (12.80%) |
| **HbA1c** (%) | 35.95 ± 6.53 | 34.59 ± 4.47 | 36.92 ± 7.53 | < 0.001 | | 35.04 ± 5.16 | 38.28 ± 8.75 | < 0.001 | 18,300 (4.67%) |
| **Triglyceride** (mg/dl) | 1.75 ± 1.02 | 1.36 ± 0.73 | 2.03 ± 1.11 | < 0.001 | | 1.58 ± 0.90 | 2.21 ± 1.17 | < 0.001 | 18,499 (4.72%) |
| **HDL** (mg/dl) |  |  |  | < 0.001 | |  |  | < 0.001 | 49,949 (12.74%) |
| Female | 1.60 ± 0.38 | 1.71 ± 0.38 | 1.46 ± 0.33 |  | | 1.65 ± 0.37 | 1.39 ± 0.31 |  |  |
| Male | 1.28 ± 0.31 | 1.43 ± 0.34 | 1.24 ± 0.29 |  | | 1.35 ± 0.32 | 1.18 ± 0.27 |  |  |
| **Hypertension**, case *n* (%) | 205,637 (52.83%) | 62,670 (38.75%) | 142,967 (62.83%) | < 0.001 | | 129,172 (46.10%) | 76,465 (70.10%) | < 0.001 | 2794 (0.71%) |
| **T2D**, case *n* (%) | 24,915 (7.59%) | 2991 (2.21%) | 21,924 (11.38%) | < 0.001 | | 8751 (3.72%) | 16,164 (17.41%) | < 0.001 | 63,925 (16.30%) |
| **CVD**, case *n* (%) | 40,245 (12.48%) | 9704 (7.48%) | 30,541 (15.86%) | < 0.001 | | 22,643 (9.94%) | 17,602 (18.62%) | < 0.001 | 69,683 (17.77%) |
| **HL**, case *n* (%) | 40,409 (12.53%) | 9083 (6.99%) | 31,326 (16.26%) | < 0.001 | | 21,996 (9.65%) | 18,413 (19.49%) | < 0.001 | 69,601 (17.75%) |
| **FL**, case *n* (%) | 755 (0.23%) | 191 (0.15%) | 564 (0.29%) | < 0.001 | | 400 (0.18%) | 355 (0.38%) | < 0.001 | 69,601 (17.75%) |

*Note*: NHW. non-Hispanic White; BMI40, BMI ≥ 40 kg/m².
