# Supplementary material for: Association of Multiple-trait Polygenic Risk Score with Obesity and Cardiometabolic Diseases in Korean Population
Source: Genomics Proteomics Bioinformatics. 2025 Nov 9;23(5):qzaf102. doi: 10.1093/gpbjnl/qzaf102 (PMC12975335; doi:10.1093/gpbjnl/qzaf102)
Supplement: qzaf102_Supplementary_Data [file qzaf102_supplementary_data.zip › 13-Feb-2026_113933_qzaf102_Supplementary_Data_TableS12Revised.zip!/qzaf102_Supplementary_Data_TableS12Revised/Table S4.docx]

**Table S4** **Significant SNPs identified from GWASs of obesity-related indicators**

| **Chr** | **Bp** | **dbSNP ID** | **Ref/**  **Alt** | **Gene** | **MAF** | **Trait** | **OR** | **STAT** | ***P* value** | **Replication *P*** | | | | |
| --- | --- | --- | --- | --- | --- | --- | --- | --- | --- | --- | --- | --- | --- | --- |
|  |  |  |  |  |  |  |  |  |  | **REP1_Kor_** | **REP2_Chi_** | **REP3_NHW_** | **BBJ** | **TWB** |
| 1 | 177873210 | rs574367 | G/T | 0.25 | *SEC16B* | BMI | 0.06 | 11.02 | 3.03×10^-28^ | **2.18×10^-5^** | 0.82 | **2.48×10^-70^** | **5.32×10^-29^** | **4.79×10^-18^** |
| 1 | 177873210 | rs574367 | G/T | 0.25 | *SEC16B** | WC | 0.04 | 7.49 | 7.15×10^-14^ | **2.33×10^-3^** | 0.86 | **4.89×10^-50^** | - | **3.98×10^-11^** |
| 2 | 632101 | rs11127486 | T/C | 0.09 | *TMEM18* | BMI | −0.07 | −8.45 | 2.94×10^-17^ | - | 0.70 | **1.23×10^-75^** | **1.90×10^-16^** | **1.12×10^-22^** |
| 2 | 632101 | rs11127486 | T/C | 0.09 | *TMEM18** | WC | −0.05 | −6.27 | 3.74×10^-10^ | - | 0.90 | **6.57×10^-59^** | - | **1.55×10^-8^** |
| 2 | 25133148 | rs6744205 | T/C | 0.45 | *ADCY3* | BMI | 0.04 | 8.90 | 5.52×10^-19^ | **5.22×10^-4^** | - | - | **1.39×10^-10^** | **3.47×10^-17^** |
| 2 | 25400559 | rs13022041 | G/T | 0.31 | *POMC** | BMI | 0.03 | 5.66 | 1.51×10^-8^ | 0.44 | - | - | **6.09×10^-4^** | **0.02** |
| 2 | 54240105 | rs6715161 | C/T | 0.01 | *ACYP2* | BMI | −0.17 | −6.99 | 2.70×10^-12^ | - | - | **9.32×10^-5^** | - | - |
| 2 | 169129145 | rs1955337 | G/T | 0.30 | *STK39* | BMI | 0.04 | 7.04 | 1.96×10^-12^ | 0.86 | 0.99 | 0.12 | **1.16×10^-9^** | **7.41×10^-6^** |
| 2 | 169129145 | rs1955337 | G/T | 0.30 | *STK39* | WC | 0.03 | 6.77 | 1.30×10^-11^ | 0.96 | 0.30 | **0.02** | - | **3.39×10^-4^** |
| 2 | 181361163 | rs1453053 | A/G | 0.31 | *SCHLAP1** | BMI | −0.03 | −5.73 | 9.80×10^-9^ | 0.60 | - | - | **9.81×10^-6^** | **2.24×10^-4^** |
| 2 | 227231563 | rs72617133 | C/G | 0.34 | *LOC646736** | WC | 0.03 | 5.69 | 1.29×10^-8^ | 0.73 | - | - | - | 0.26 |
| 3 | 52893465 | rs6798941 | C/T | 0.37 | *TMEM110* | BMI | 0.04 | 8.38 | 5.39×10^-17^ | **5.60×10^-3^** | - | - | **8.94×10^-8^** | **8.51×10^-5^** |
| 4 | 45182527 | rs10938397 | A/G | 0.28 | *GNPDA2** | BMI | 0.04 | 6.84 | 7.76×10^-12^ | 0.07 | 0.19 | **6.11×10^-41^** | **7.98×10^-13^** | **1.10×10^-19^** |
| 4 | 45182527 | rs10938397 | A/G | 0.28 | *GNPDA2** | WC | 0.03 | 5.77 | 8.19×10^-9^ | **0.04** | 0.35 | **8.14×10^-29^** | - | **3.89×10^-12^** |
| 5 | 87970352 | rs112862634 | G/C | 0.50 | *LINC00461** | BMI | 0.03 | 6.16 | 7.51×10^-10^ | 0.21 | - | - | **1.76×10^-13^** | **1.10×10^-7^** |
| 5 | 95835574 | rs7713143 | C/T | 0.44 | *LOC101929710* | BMI | 0.03 | 5.90 | 3.54×10^-9^ | 0.07 | - | - | **1.03×10^-21^** | **5.13×10^-14^** |
| 5 | 122750847 | rs6595447 | T/C | 0.32 | *CEP120** | BMI | −0.03 | −6.1 | 1.05×10^-9^ | **4.78****×10^-3^** | - | - | **2.99×10^-6^** | **9.77×10^-3^** |
| 6 | 20686878 | rs67131976 | C/T | 0.46 | *CDKAL1* | BMI | −0.03 | −6.76 | 1.35×10^-11^ | **0.05** | 0.20 | **1.06×10^-6^** | 1.32×10^-28^ | **2.82×10^-5^** |
| 6 | 20686878 | rs67131976 | C/T | 0.46 | *CDKAL1** | WC | −0.02 | −5.45 | 4.95×10^-8^ | 0.32 | 0.10 | **1.06×10^-4^** | - | 0.14 |
| 6 | 28782998 | rs16897420 | C/T | 0.38 | *LINC01623** | BMI | −0.03 | −5.72 | 1.09×10^-8^ | - | 0.79 | 0.07 | - | **2.09×10^-3^** |
| 7 | 138817193 | rs11525873 | T/C | 0.29 | *TTC26** | BMI | −0.03 | −5.84 | 5.40×10^-9^ | - | 0.51 | **9.05×10^-8^** | **6.26×10^-4^** | **5.25×10^-3^** |
| 10 | 31800583 | **rs220063** | A/G | 0.19 | ***ZEB1**** | BMI | −0.03 | −5.49 | 3.95×10^-8^ | - | - | - | **9.30×10^-4^** | **1.55×10^-4^** |
| 10 | 77437044 | rs2454812 | T/C | 0.48 | *LRMDA** | BMI | −0.03 | −5.83 | 5.47×10^-9^ | 0.62 | - | - | **0.01** | 0.93 |
| 10 | 104222963 | rs12570201 | T/C | 0.25 | *MFSD13A** | BMI | −0.03 | −5.89 | 3.85×10^-9^ | 0.28 | 0.66 | **5.25×10^-4^** | **0.02** | 0.12 |
| 11 | 8612000 | rs4418812 | A/G | 0.41 | *STK33** | BMI | 0.03 | 5.68 | 1.32×10^-8^ | 0.97 | 0.47 | **3.32×10^-8^** | **1.53×10^-6^** | **8.32×10^-5^** |
| 11 | 27650524 | rs10742179 | A/G | 0.50 | *BDNF-AS** | WC | 0.03 | 6.95 | 3.58×10^-12^ | - | - | - | - | **3.80×10^-5^** |
| 11 | 27704209 | rs34379767 | G/A | 0.46 | *BDNF-AS** | BMI | −0.05 | −9.84 | 8.08×10^-23^ | **2.75×10^-3^** | 0.52 | **4.13×10^-45^** | **5.97×10^-25^** | **4.90×10^-14^** |
| 12 | 111684461 | rs59214806 | A/G | 0.49 | *CUX2** | WC | 0.03 | 6.42 | 1.38×10^-10^ | 0.37 | - | - | - | 0.14 |
| 12 | 111963570 | rs11065939 | T/C | 0.47 | *ATXN2** | WC | 0.03 | 5.79 | 6.92×10^-9^ | 0.37 | - | - | - | 0.18 |
| 13 | 54084032 | rs4883723 | G/A | 0.28 | *LINC00558* | BMI | 0.04 | 6.85 | 7.45×10^-12^ | - | - | - | **1.89×10^-7^** | **3.55×10^-7^** |
| 13 | 54084032 | rs4883723 | G/A | 0.28 | *LINC00558** | WC | 0.03 | 5.53 | 3.21×10^-8^ | - | - | - | - | **3.80×10^-3^** |
| 16 | 20037123 | rs57705530 | G/A | 0.39 | *GPR139** | BMI | 0.03 | 5.46 | 4.66×10^-8^ | **0.02** | 0.28 | **2.44×10^-8^** | **8.30×10^-7^** | **1.17×10^-5^** |
| 16 | 20257039 | rs4420537 | C/T | 0.22 | *GP2* | BMI | −0.04 | −6.45 | 1.11×10^-10^ | 0.08 | - | - | **7.87×10^-11^** | **7.24×10^-18^** |
| 16 | 30068354 | rs9939774 | C/T | 0.32 | *ALDOA** | WC | 0.03 | 6.48 | 8.96×10^-11^ | **0.04** | 0.88 | **2.60×10^-30^** | - | **1.35×10^-9^** |
| 16 | 30093779 | rs2278557 | C/G | 0.32 | *PPP4C** | BMI | 0.03 | 6.68 | 2.39×10^-11^ | **7.86×10^-3^** | 0.53 | **4.53×10^-25^** | **1.98×10^-5^** | **9.55×10^-11^** |
| 16 | 53839135 | rs8044769 | C/T | 0.39 | *FTO* | BMI | −0.03 | −5.59 | 2.30×10^-8^ | - | - | - | **3.21×10^-11^** | **2.14×10^-8^** |
| 16 | 53845487 | rs11642841 | C/A | 0.03 | *FTO* | BMI | 0.09 | 7.07 | 1.57×10^-12^ | - | - | - | **7.69×10^-32^** | **7.76×10^-13^** |
| 16 | 53845487 | rs11642841 | C/A | 0.03 | *FTO** | WC | 0.08 | 6.10 | 1.05×10^-9^ | - | - | - | - | **1.74×10^-11^** |
| 17 | 46143042 | rs7209484 | T/C | 0.26 | *NFE2L1******** | WC | −0.03 | −5.80 | 6.77×10^-9^ | - | - | - | - | **1.17×10^-4^** |
| 17 | 79047278 | rs35867081 | G/A | 0.36 | *BAIAP2** | BMI | 0.03 | 5.66 | 1.50×10^-8^ | - | - | - | **1.16×10^-3^** | **2.63×10^-7^** |
| 18 | 57852587 | rs476828 | T/C | 0.27 | *MC4R* | BMI | 0.07 | 12.26 | 1.65×10^-34^ | **0.01** | 0.75 | **5.14×10^-85^** | **2.05×10^-29^** | **1.91×10^-16^** |
| 18 | 57852587 | rs476828 | T/C | 0.27 | *MC4R* | WC | 0.05 | 9.67 | 4.07×10^-22^ | **0.03** | 0.54 | **4.54×10^-78^** | **3.97×10^-5^** | **5.62×10^-5^** |
| 18 | 63441752 | rs8096722 | C/G | 0.31 | *CDH7** | BMI | 0.03 | 5.96 | 2.57×10^-9^ | 0.93 | - | - | - | **9.77×10^-12^** |
| 19 | 46177235 | rs55669001 | C/T | 0.47 | *GIPR* | BMI | 0.04 | 8.25 | 1.60×10^-16^ | - | - | - | **5.56×10^-39^** | **9.12×10^-7^** |
| 19 | 46221726 | **rs11881883** | G/A | 0.40 | ***FBXO46*** | WC | −0.03 | −6.82 | 9.10×10^-12^ | - | - | - | - | **4.17×10^-7^** |
| 19 | 46267453 | rs16980013 | G/T | 0.20 | *BHMG1** | BMI | 0.03 | 5.48 | 4.38×10^-8^ | 0.50 | 0.70 | 0.72 | **3.24×10^-12^** | **0.01** |
| 19 | 47588325 | rs2287842 | G/A | 0.21 | *ZC3H4** | BMI | 0.04 | 6.34 | 2.34×10^-10^ | **9.58×10^-3^** | 0.06 | **2.38×10^-8^** | - | **6.31×10^-8^** |
| 20 | 57469073 | rs6026584 | C/T | 0.35 | *GNAS** | WC | −0.03 | −5.60 | 2.18×10^-8^ | 0.89 | 0.40 | **8.89×10^-4^** | - | **8.91×10^-3^** |
| 20 | 60574216 | **rs2296083** | T/C | 0.31 | *TAF4** | BMI | 0.03 | 5.69 | 1.26×10^-8^ | **0.05** | 0.28 | 0.23 | **7.15×10^-8^** | **8.51×10^-5^** |
| 21 | 40309591 | **rs4817972** | C/G | 0.32 | ***LOC400867**** | BMI | 0.03 | 5.66 | 1.48×10^-8^ | 0.79 | - | - | **2.47×10^-4^** | **1.20×10^-8^** |

*Note*: BBJ, Biobank Japan; TWB, Taiwan Biobank. “*****” mark indicates gene regions that do not contain genome-wide significant SNPs associated with obesity, as defined by the obesity-related indicators listed in the Trait column. MAF, minor allele frequency; OR, odds ratio; Proxy *P* values refer to P values from BBJ and TWB for the obesity-related indicators used to define obesity in the Trait column.
