# Supplementary material for: Association of Multiple-trait Polygenic Risk Score with Obesity and Cardiometabolic Diseases in Korean Population
Source: Genomics Proteomics Bioinformatics. 2025 Nov 9;23(5):qzaf102. doi: 10.1093/gpbjnl/qzaf102 (PMC12975335; doi:10.1093/gpbjnl/qzaf102)
Supplement: qzaf102_Supplementary_Data [file qzaf102_supplementary_data.zip › 13-Feb-2026_113933_qzaf102_Supplementary_Data_TableS12Revised.zip!/qzaf102_Supplementary_Data_TableS12Revised/Table S5.docx]

**Table S5 Significant SNPs identified from GWAS of obesity-related diseases**

| **Chr** | **Bp** | **dbSNP ID** | **Ref/**  **Alt** | **Gene** | **MAF** | **Trait** | **OR** | **STAT** | ***P* value** | **Replication *P*** | | | | |
| --- | --- | --- | --- | --- | --- | --- | --- | --- | --- | --- | --- | --- | --- | --- |
|  |  |  |  |  |  |  |  |  |  | **REP1_Kor_** | **REP2_Chi_** | **REP3_EUR_** | **BBJ** | **TWB** |
| 1 | 10799577 | rs12046278 | C/T | 0.24 | *CASZ1* | HT | 0.91 | −5.62 | 1.68×10^-8^ | - | - | - | - | - |
| 1 | 113046879 | rs3790604 | C/A | 0.31 | *WNT2B* | HT | 1.09 | 6.04 | 1.72×10^-9^ | - | - | **8.42×10^-17^** | - | - |
| 2 | 26916969 | rs1275980 | C/T | 0.22 | *KCNK3* | HT | 0.86 | −9.39 | 2.97×10^-21^ | - | - | - | - | - |
| 2 | 54924901 | rs138938305 | C/T | 0.03 | *SPTBN1* | HT | 1.36 | 8.00 | 4.48×10^-15^ | - | - | - | - | - |
| 2 | 165132714 | rs1561467 | T/A | 0.33 | *GRB14* | HT | 0.92 | −5.72 | 9.68×10^-9^ | - | - | - | - | - |
| 3 | 14938030 | rs139325822 | T/C | 0.28 | *FGD5* | HT | 1.09 | 5.64 | 1.92×10^-8^ | - | - | - | - | - |
| 3 | 53548031 | rs59968724 | C/G | 0.11 | *CACNA1D* | HT | 1.13 | 5.90 | 4.78×10^-9^ | - | - | - | - | - |
| 3 | 53597491 | rs807191 | A/C | 0.47 | *CACNA1D* | HT | 1.10 | 6.78 | 1.25×10^-11^ | - | - | - | - | - |
| 4 | 81155937 | rs79620921 | A/T | 0.26 | *PRDM8* | HT | 1.13 | 7.79 | 9.12×10^-15^ | - | - | - | - | - |
| 4 | 81166686 | rs140801183 | A/G | 0.02 | *FGF5* | HT | 1.31 | 5.97 | 4.60×10^-9^ | - | - | - | - | - |
| 4 | 81207963 | rs3733336 | A/G | 0.21 | *FGF5* | HT | 1.17 | 9.86 | 1.27×10^-22^ | - | - | - | - | - |
| 5 | 32829929 | rs1177765 | C/T | 0.39 | *NPR3* | HT | 0.93 | −5.47 | 4.40×10^-8^ | - | - | **1.01×10^-26^** | - | - |
| 5 | 122460332 | rs2287696 | A/G | 0.43 | *PRDM6* | HT | 0.92 | −6.23 | 4.40×10^-10^ | - | - | - | - | - |
| 6 | 127142458 | rs62426324 | C/T | 0.47 | *RSPO3* | HT | 1.08 | 5.54 | 3.12×10^-8^ | - | - | - | - | - |
| 7 | 80328352 | rs143722364 | T/C | 0.07 | *CD36* | HT | 1.16 | 5.68 | 1.84×10^-8^ | - | - | - | - | - |
| 8 | 144005285 | rs7011901 | A/G | 0.49 | *CYP11B2* | HT | 0.91 | −7.32 | 2.53×10^-13^ | - | - | - | - | - |
| 10 | 104386309 | rs4285804 | T/A | 0.26 | *SUFU* | HT | 1.09 | 5.77 | 9.08×10^-9^ | - | - | 0.08 | - | - |
| 10 | 104928914 | rs12416331 | T/A | 0.24 | *NT5C2* | HT | 0.89 | −7.54 | 3.46×10^-14^ | - | - | - | - | - |
| 10 | 115725280 | rs74157561 | A/G | 0.12 | *NHLRC2* | HT | 1.12 | 5.64 | 2.11×10^-8^ | - | - | - | - | - |
| 11 | 116882638 | rs4245168 | C/A | 0.50 | *SIK3* | HT | 0.92 | −5.99 | 2.12×10^-9^ | - | - | - | - | - |
| 12 | 90058842 | rs111478946 | G/A | 0.37 | *ATP2B1* | HT | 0.87 | −9.70 | 2.24×10^-22^ | - | - | **2.51×10^-29^** | - | - |
| 12 | 111467605 | rs73427603 | C/T | 0.24 | *CUX2* | HT | 0.91 | −6.18 | 5.43×10^-10^ | - | - | - | - | - |
| 12 | 115553034 | rs35442 | G/A | 0.24 | *TBX3* | HT | 0.89 | −7.31 | 2.07×10^-13^ | - | - | - | - | - |
| 13 | 30146201 | rs9508495 | C/T | 0.35 | *SLC7A1* | HT | 0.92 | −6.20 | 5.35×10^-10^ | - | - | - | - | - |
| 17 | 78358945 | rs112735431 | G/A | 0.01 | *RNF213* | HT | 1.78 | 9.87 | 1.28×10^-21^ | - | - | - | - | - |
| 19 | 11526765 | rs167479 | G/T | 0.49 | *RGL3* | HT | 0.91 | −7.29 | 2.98×10^-13^ | - | - | - | - | - |
| 20 | 10965998 | rs1887320 | A/G | 0.48 | *C20orf187* | HT | 0.90 | −7.84 | 4.29×10^-15^ | - | - | **2.08×10^-27^** | - | - |
| 2 | 234346270 | rs117447187 | T/C | 0.03 | *DGKD* | T2D | 1.39 | 6.09 | 4.65×10^-9^ | - | - | - | - | - |
| 3 | 23298683 | rs9866739 | C/T | 0.19 | *UBE2E2* | T2D | 0.86 | −5.86 | 3.07×10^-9^ | - | - | 0.20 | **6.82×10^-20^** | - |
| 3 | 63826364 | rs73120894 | T/C | 0.26 | *C3orf49* | T2D | 0.88 | −5.52 | 2.71×10^-8^ | - | - | 0.83 | **1.39×10^-12^** | - |
| 3 | 187121145 | rs79282614 | A/G | 0.01 | *RTP4* | T2D | 1.54 | 5.74 | 4.84×10^-8^ | - | - | - | - | - |
| 6 | 20682622 | rs35612982 | T/C | 0.46 | *CDKAL1* | T2D | 1.32 | 14.39 | 2.43×10^-46^ | - | - | - | **1.93×10^-91^** | - |
| 7 | 127253550 | rs2233580 | C/T | 0.07 | *PAX4* | T2D | 1.42 | 10.44 | 9.45×10^-24^ | - | - | - | **6.30×10^-90^** | - |
| 8 | 118184783 | rs13266634 | C/T | 0.40 | *SLC30A8* | T2D | 0.87 | −7.13 | 8.69×10^-13^ | - | - | 0.07 | **1.22×10^-37^** | - |
| 9 | 22134068 | rs10811660 | G/A | 0.44 | *CDKN2B-AS1* | T2D | 0.80 | −11.68 | 1.54×10^-31^ | - | - | **0.01** | **5.37×10^-98^** | - |
| 10 | 12309139 | rs11257657 | C/G | 0.45 | *CDC123* | T2D | 1.14 | 6.86 | 7.79×10^-12^ | - | - | - | **2.71×10^-32^** | - |
| 10 | 94150879 | rs12260406 | G/A | 0.23 | *MARK2P9* | T2D | 1.14 | 5.85 | 6.82×10^-9^ | - | - | - | **1.82×10^-9^** | - |
| 10 | 94466439 | rs12219514 | G/A | 0.15 | *HHEX* | T2D | 1.23 | 8.09 | 2.10×10^-15^ | - | - | 0.99 | **1.05×10^-41^** | - |
| 10 | 94735056 | rs549156247 | C/G | 0.04 | *EXOC6* | T2D | 1.32 | 6.04 | 4.89×10^-9^ | - | - | - | **4.76×10^-21^** | - |
| 18 | 57808978 | rs66723169 | C/A | 0.23 | *MC4R* | T2D | 1.14 | 5.89 | 5.35×10^-9^ | - | - | - | **8.76×10^-13^** | - |
| 20 | 42972856 | rs8115481 | G/T | 0.50 | *R3HDML* | T2D | 0.89 | −5.93 | 3.05×10^-9^ | - | - | - | **8.80×10^-6^** | - |
| 17 | 78267829 | rs954846345 | C/G | 0.01 | *RNF213* | CVD | 2.09 | 7.27 | 2.05×10^-11^ | - | - | - | - | - |
| 1 | 109822166 | rs599839 | A/G | 0.06 | *PSRC1* | HL | 0.79 | −6.47 | 3.21×10^-11^ | - | - | - | - | - |
| 2 | 20849856 | rs116948525 | G/C | 0.04 | *HS1BP3* | HL | 1.25 | 5.81 | 1.41×10^-8^ | - | - | - | - | - |
| 2 | 21195522 | rs72788559 | G/T | 0.13 | *APOB* | HL | 0.85 | −6.34 | 1.27×10^-10^ | - | - | 0.72 | - | - |
| 2 | 21242731 | rs13306206 | G/A | 0.01 | *APOB* | HL | 1.85 | 9.20 | 4.08×10^-18^ | - | - | - | - | - |
| 2 | 27742603 | rs780093 | T/C | 0.46 | *GCKR* | HL | 0.90 | −5.95 | 2.61×10^-9^ | - | - | - | - | - |
| 5 | 74617262 | rs2878417 | A/G | 0.48 | *HMGCR* | HL | 0.90 | −6.48 | 9.30×10^-11^ | - | - | 0.60 | - | - |
| 8 | 126482621 | rs2954022 | A/C | 0.44 | *TRIB1* | HL | 1.14 | 8.08 | 7.77×10^-16^ | - | - | **0.05** | - | - |
| 11 | 116639692 | rs1268353 | C/T | 0.22 | *BUD13* | HL | 0.88 | −5.91 | 2.57×10^-9^ | - | - | 0.42 | - | - |
| 11 | 116707684 | rs2070665 | G/A | 0.36 | *APOA1-AS* | HL | 1.11 | 6.08 | 1.36×10^-9^ | - | - | - | - | - |
| 11 | 117001960 | rs12421425 | C/T | 0.12 | *PAFAH1B2* | HL | 0.86 | −5.63 | 1.14×10^-8^ | - | - | 0.40 | - | - |
| 16 | 72009439 | **rs6499554** | G/A | 0.30 | *PKD1L3* | HL | 0.90 | −5.86 | 3.79×10^-9^ | - | - | **1.57×10^-4^** | - | - |
| 18 | 47142243 | rs17726130 | G/A | 0.28 | *LIPG* | HL | 1.12 | 6.17 | 8.81×10^-10^ | - | - | 0.41 | - | - |
| 19 | 11242307 | rs2738464 | C/G | 0.29 | *LDLR* | HL | 0.87 | −7.17 | 5.37×10^-13^ | - | - | - | - | - |
| 19 | 11346155 | rs56322906 | G/A | 0.27 | *DOCK6* | HL | 0.89 | −6.08 | 9.38×10^-10^ | - | - | - | - | - |
| 19 | 45412079 | rs7412 | C/T | 0.06 | *APOE* | HL | 0.69 | −9.78 | 2.38×10^-24^ | - | - | **2.24×10^-4^** | - | - |
| 22 | 44324727 | rs738409 | C/G | 0.42 | *PNPLA3* | FL | 1.22 | 8.04 | 1.42×10^-15^ | - | - | 0.87 | - | - |

*Note*: “*****” mark indicates gene regions that overlap with genome-wide significant SNPs associated with obesity.
