# Supplementary material for: Association of Multiple-trait Polygenic Risk Score with Obesity and Cardiometabolic Diseases in Korean Population
Source: Genomics Proteomics Bioinformatics. 2025 Nov 9;23(5):qzaf102. doi: 10.1093/gpbjnl/qzaf102 (PMC12975335; doi:10.1093/gpbjnl/qzaf102)
Supplement: qzaf102_Supplementary_Data [file qzaf102_supplementary_data.zip › 13-Feb-2026_113933_qzaf102_Supplementary_Data_TableS12Revised.zip!/qzaf102_Supplementary_Data_TableS12Revised/Table S6.docx]

**Table S6** **Genomic inflation, SNP heritability, and genetic correlations for obesity-related indicators and obesity-related diseases**

**A. Genomic inflation factors and SNP heritability estimates for obesity-related indicators**

| **Dataset** |  | **General obesity** | | | **Abdominal obesity** | | |
| --- | --- | --- | --- | --- | --- | --- | --- |
|  | **Estimator** | **BMI** | **BMI25/BMI30** | **BMI30/BMI40** | **WC** | **WC1** | **WC2** |
| Discovery | 𝜆_𝐺𝐶_ | 1.26 | 1.22 | 1.08 | 1.17 | 1.16 | 1.08 |
|  | 𝜆_𝐿𝐷𝑆𝐶_ | 1.04 | 1.04 | 1.00 | 1.03 | 1.03 | 1.02 |
|  | *h^2^* (P) | **0.21 (1.07 × 10^-62^)** | **0.12 (1.36 × 10^-34^)** | **0.04 (6.56 × 10^-11^)** | **0.15 (3.66 × 10^-46^)** | **0.09 (2.14 × 10^-23^)** | **0.04 (8.65 × 10^-8^)** |
| REP1_Kor_ | 𝜆_𝐺𝐶_ | 1.02 | 1.02 | 0.93 | 1.02 | 1.06 | 0.94 |
|  | 𝜆_𝐿𝐷𝑆𝐶_ | 1.01 | 1.01 | 0.91 | 1.01 | 1.06 | 0.93 |
|  | *h^2^* (P) | **0.19 (0.04)** | 0.09 (0.30) | 0.07 (0.30) | **0.15 (0.04)** | 0.03 (0.72) | -0.02 (0.82) |
| REP2_Chi_ | 𝜆_𝐺𝐶_ | 0.99 | 0.83 | 0.72 | 0.98 | 0.81 | 0.75 |
|  | 𝜆_𝐿𝐷𝑆𝐶_ | 1.01 | 0.84 | 0.74 | 1.01 | 0.82 | 0.74 |
|  | *h^2^* (P) | -0.37 (0.27) | -0.13 (0.67) | -0.49 (0.05) | -0.48 (0.21) | -0.36 (0.18) | -0.01 (0.97) |
| REP3_NHW_ | 𝜆_𝐺𝐶_ | 1.91 | 1.92 | 1.27 | 1.76 | 1.76 | 1.75 |
|  | 𝜆_𝐿𝐷𝑆𝐶_ | 1.55 | 1.33 | 1.08 | 1.47 | 1.28 | 1.29 |
|  | *h^2^* (P) | **0.20 (2.44 × 10^-92^)** | **0.12 (6.69 × 10^-77^)** | **0.03 (3.45 × 10^-30^)** | **0.16 (2.55 × 10^-89^)** | **0.10 (4.84 × 10^-87^)** | **0.10 (2.46 × 10^-80^)** |

**B. Genomic inflation factors and SNP heritability estimates for obesity-related diseases**

| **Dataset** | **Estimator** | **HT** | **T2D** | **CVD** | **HL** | **FL** |
| --- | --- | --- | --- | --- | --- | --- |
| Discovery | 𝜆_𝐺𝐶_ | 1.15 | 1.10 | 1.04 | 1.07 | 1.03 |
|  | 𝜆_𝐿𝐷𝑆𝐶_ | 1.02 | 1.02 | 1.01 | 1.03 | 1.01 |
|  | *h^2^* (P) | **0.11 (3.63 × 10^-25^)** | **0.06 (3.31 × 10^-11^)** | **0.01 (0.02)** | **0.04 (3.25 × 10^-7^)** | 0.01 (0.11) |
| REP3_NHW_ | 𝜆_𝐺𝐶_ | 1.75 | 1.36 | 1.25 | 1.25 | 0.98 |
|  | 𝜆_𝐿𝐷𝑆𝐶_ | 1.30 | 1.15 | 1.09 | 1.09 | 1.00 |
|  | *h^2^* (P) | **0.10 (1.39 × 10^-73^)** | **0.05 (4.66 × 10^-25^)** | **0.03 (8.16 × 10^-33^)** | **0.04 (1.68 × 10^-24^)** | -1.20×10^-4^ (0.39) |

**C. Pairwise genetic correlations among obesity-related indicators and obesity-related diseases**

| **Dataset** | **Estimator** | **HT** | **T2D** | **CVD** | **HL** | **FL** |
| --- | --- | --- | --- | --- | --- | --- |
| Discovery | *GC_BMI25_* (P) | **0.27 (6.76 × 10^-6^)** | **0.31 (2.00 × 10^-4^)** | **0.28 (0.05)** | 0.13 (0.16) | 0.29 (0.05) |
|  | *GC_WC1_* (P) | **0.30 (4.38 × 10^-6^)** | **0.42 (2.96 × 10^-6^)** | **0.36 (0.04)** | **0.19 (0.05)** | 0.20 (0.20) |
| REP3_NHW_ | *GC_BMI30_* (P) | **0.36 (1.77 × 10^-34^)** | **0.59 (1.83 × 10^-51^)** | **0.40 (2.24 × 10^-27^)** | **0.44 (2.56 × 10^-19^)** | nan (nan) |
|  | *GC_WC1_* (P) | **0.33 (1.19 × 10^-36^)** | **0.65 (1.06 × 10^-71^)** | **0.42 (1.01 × 10^-34^)** | **0.44 (8.69 × 10^-18^)** | nan (nan) |

*Note*: GC, genetic correlation; 𝜆_𝐺𝐶_, genomic inflation factor; 𝜆_𝐿𝐷𝑆𝐶_., LD score intercept; *h²* (*P* value), SNP heritability estimate with P value; *GC_BMI25/BMI30_* (*P* value) and *GC_WC1_* (*P* value), genetic correlations between obesity and obesity related diseases, with corresponding P values in parentheses; nan, not available due to out-of-bound heritability estimates.
