# Supplementary material for: Association of Multiple-trait Polygenic Risk Score with Obesity and Cardiometabolic Diseases in Korean Population
Source: Genomics Proteomics Bioinformatics. 2025 Nov 9;23(5):qzaf102. doi: 10.1093/gpbjnl/qzaf102 (PMC12975335; doi:10.1093/gpbjnl/qzaf102)
Supplement: qzaf102_Supplementary_Data [file qzaf102_supplementary_data.zip › 13-Feb-2026_113933_qzaf102_Supplementary_Data_TableS12Revised.zip!/qzaf102_Supplementary_Data_TableS12Revised/Table S8.docx]

**Table S8**  **Significant genes identified through gene-based analyses of obesity-related indicators and obesity-related diseases**

|  |  | ***P* for general obesity by BMI** | ***P* for abdominal obesity by WC** | ***P* for HT** | ***P* for T2D** | ***P* for HL** | ***P* for FL** |
| --- | --- | --- | --- | --- | --- | --- | --- |
| **Chr** | **Gene** | **Discovery**  **(REP1_Kor_/REP2_Chi_/REP3_NHW_)** | **Discovery**  **(REP1_Kor_/REP2_Chi_/REP3_NHW_)** | **Discovery**  **(REP3_NHW_)** | **Discovery**  **(REP3_NHW_)** | **Discovery**  **(REP3_NHW_)** | **Discovery**  **(REP3_NHW_)** |
| 1 | *TCEA3* | - (-/-/-) | - (-/-/-) | 2.22×10^-3^ (0.06) | - (-) | - (-) | - (-) |
| 1 | *HIVEP3* | **8.36×10^-7^ (**0.17/0.72/**7.28×10^-3^)** | - (-/-/-) | - (-) | - (-) | - (-) | - (-) |
| 1 | *CELSR2* | - (-/-/-) | - (-/-/-) | - (-) | - (-) | **1.46×10^-9^ (5.00×10^-10^)** | - (-) |
| 1 | *WNT2B* | - (-/-/-) | - (-/-/-) | **7.80×10^-7^ (6.11×10^-16^)** | - (-) | - (-) | - (-) |
| 2 | *ADCY3* | **2.57×10^-13^ (1.82×10^-4^**/0.62/**5.00×10^-10^)** | - (-/-/-) | - (-) | - (-) | - (-) | - (-) |
| 2 | *EFR3B* | **3.73×10^-8^ (**0.11/0.41/**2.81×10^-9^)** | - (-/-/-) | - (-) | - (-) | - (-) | - (-) |
| 2 | *KCNK3* | - (-/-/-) | - (-/-/-) | **8.06×10^-13^ (9.05×10^-15^)** | - (-) | - (-) | - (-) |
| 2 | *GPR75ASB3* | 6.62×10^-7^ (0.29/0.77/0.07) | - (-/-/-) | - (-) | - (-) | - (-) | - (-) |
| 2 | *CHAC2* | 2.53×10^-6^ (0.07/0.40/0.51) | - (-/-/-) | - (-) | - (-) | - (-) | - (-) |
| 2 | *PSME4* | 1.34×10^-7^ (0.56/0.93/0.12) | - (-/-/-) | - (-) | - (-) | - (-) | - (-) |
| 2 | *ACYP2* | **5.88×10^-8^ (**0.19/0.10/**5.14×10^-3^)** | - (-/-/-) | - (-) | - (-) | - (-) | - (-) |
| 2 | ***SPTBN1*** | - (-/-/-) | - (-/-/-) | **4.09×10^-9^ (9.66×10^-3^)** | - (-) | - (-) | - (-) |
| 2 | *EML6* | - (-/-/-) | - (-/-/-) | **3.08×10^-10^ (4.02×10^-3^)** | - (-) | - (-) | - (-) |
| 3 | *FGD5* | - (-/-/-) | - (-/-/-) | **7.73×10^-8^ (2.78×10^-10^)** | - (-) | - (-) | - (-) |
| 3 | *UBE2E2* | - (-/-/-) | - (-/-/-) | - (-) | **3.42×10^-7^ (1.16×10^-9^)** | - (-) | - (-) |
| 3 | *STAB1* | **3.78×10^-7^ (**0.07/0.23/**1.24×10^-3^**) | - (-/-/-) | - (-) | - (-) | - (-) | - (-) |
| 3 | *NT5DC2* | **5.11×10^-8^ (0.05**/0.20/7.95**×10^-6^**) | - (-/-/-) | - (-) | - (-) | - (-) | - (-) |
| 3 | *SMIM4* | **4.31×10^-8^ (0.05**/0.36/**7.25×10^-4^**) | - (-/-/-) | - (-) | - (-) | - (-) | - (-) |
| 3 | *PBRM1* | **3.13×10^-8^ (**0.06/0.41/**9.96×10^-6^**) | - (-/-/-) | - (-) | - (-) | - (-) | - (-) |
| 3 | *GNL3* | **5.08×10^-8^ (**0.07/0.37/**7.38×10^-7^**) | - (-/-/-) | - (-) | - (-) | - (-) | - (-) |
| 3 | *GLT8D1* | **3.82×10^-8^ (**0.08/0.42/**5.19×10^-6^**) | - (-/-/-) | - (-) | - (-) | - (-) | - (-) |
| 3 | *NEK4* | **3.23×10^-8^ (**0.07/0.39/**1.31×10^-5^**) | - (-/-/-) | - (-) | - (-) | - (-) | - (-) |
| 3 | *ITIH1* | **1.11×10^-8^ (**0.06/0.82/**1.68×10^-6^**) | - (-/-/-) | - (-) | - (-) | - (-) | - (-) |
| 3 | *MUSTN1* | **1.47×10^-6^ (**0.12/0.63/**3.10×10^-6^**) | - (-/-/-) | - (-) | - (-) | - (-) | - (-) |
| 3 | *TMEM110* | **8.04×10^-7^ (**0.08/0.74/**2.29×10^-8^**) | - (-/-/-) | - (-) | - (-) | - (-) | - (-) |
| 3 | *SFMBT1* | **4.12×10^-7^ (**0.31/0.99/**2.54×10^-7^**) | - (-/-/-) | - (-) | - (-) | - (-) | - (-) |
| 3 | *CACNA1D* | - (-/-/-) | - (-/-/-) | **3.52×10^-9^ (1.34×10^-9^)** | - (-) | - (-) | - (-) |
| 3 | *IGF2BP2* | - (-/-/-) | - (-/-/-) | - (-) | **3.76×10^-7^ (1.11×10^-12^)** | - (-) | - (-) |
| 4 | *CRIPAK* | - (-/-/-) | - (-/-/-) | - (-) | 1.03×10^-6^ (0.30) | - (-) | - (-) |
| 4 | *FGF5* | - (-/-/-) | - (-/-/-) | **3.89×10^-15^ (6.00×10^-10^)** | - (-) | - (-) | - (-) |
| 4 | *USP38* | - (-/-/-) | - (-/-/-) | **2.05×10^-6^ (7.06×10^-12^)** | - (-) | - (-) | - (-) |
| 5 | *HMGCR* | - (-/-/-) | - (-/-/-) | - (-) | - (-) | **5.05×10^-7^ (1.78×10^-15^**) | - (-) |
| 5 | *PRDM6* | - (-/-/-) | - (-/-/-) | **1.31×10^-9^ (1.98×10^-4^)** | - (-) | - (-) | - (-) |
| 6 | *CDKAL1* | **3.02×10^-8^ (4.12×10^-3^**/0.67/**4.25×10^-3^**) | - (-/-/-) | - (-) | **2.52×10^-16^ (3.19×10^-16^)** | - (-) | - (-) |
| 6 | *C6orf1* | - (-/-/-) | **3.13×10^-8^ (**0.96/0.35/**1.81×10^-10^**) | - (-) | - (-) | - (-) | - (-) |
| 7 | *AUTS2* | **1.57×10^-6^ (**0.66/0.24/**2.67×10^-5^**) | - (-/-/-) | - (-) | - (-) | - (-) | - (-) |
| 8 | *GML* | - (-/-/-) | - (-/-/-) | **1.52×10^-10^ (1.13×10^-4^**) | - (-) | - (-) | - (-) |
| 8 | *CYP11B2* | - (-/-/-) | - (-/-/-) | 8.52×10^-14^ (**1.51×10^-4^**) | - (-) | - (-) | - (-) |
| 9 | *NUTM2F* | 1.19×10^-6^ (-/0.95/0.77) | - (-/-/-) | - (-) | - (-) | - (-) | - (-) |
| 9 | *MAPKAP1* | - (-/-/-) | - (-/-/-) | **1.81×10^-6^ (1.83×10^-6^)** | - (-) | - (-) | - (-) |
| 9 | ***STXBP1*** | - (-/-/-) | - (-/-/-) | **1.26×10^-6^ (2.78×10^-3^)** | - (-) | - (-) | - (-) |
| 9 | ***PTRH1*** | - (-/-/-) | - (-/-/-) | **2.01×10^-6^ (8.24×10^-4^)** | - (-) | - (-) | - (-) |
| 9 | ***TTC16*** | - (-/-/-) | - (-/-/-) | **2.11×10^-6^ (1.11×10^-3^)** | - (-) | - (-) | - (-) |
| 10 | *CDC123* | - (-/-/-) | - (-/-/-) | - (-) | **1.71×10^-6^ (4.29×10^-11^)** | - (-) | - (-) |
| 10 | *C10orf11* | **1.35×10^-6^ (**0.07/0.21/**0.04**) | - (-/-/-) | - (-) | 2.50×10^-6^ (0.18) | - (-) | - (-) |
| 10 | *IDE* | - (-/-/-) | - (-/-/-) | - (-) | **5.87×10^-9^ (1.11×10^-6^)** | - (-) | - (-) |
| 10 | *TMEM180* | 1.25×10^-6^ (0.72/0.72/0.28) | - (-/-/-) | 4.99×10^-9^ (0.28) | - (-) | - (-) | - (-) |
| 10 | *SUFU* | - (-/-/-) | - (-/-/-) | 2.78×10^-9^ (0.21) | - (-) | - (-) | - (-) |
| 10 | *TRIM8* | - (-/-/-) | - (-/-/-) | 2.27×10^-6^ (0.11) | - (-) | - (-) | - (-) |
| 10 | ***ARL3*** | - (-/-/-) | - (-/-/-) | **7.70×10^-11^ (1.63×10^-3^)** | - (-) | - (-) | - (-) |
| 10 | ***SFXN2*** | - (-/-/-) | - (-/-/-) | **1.11×10^-6^ (2.13×10^-3^)** | - (-) | - (-) | - (-) |
| 10 | *WBP1L* | - (-/-/-) | - (-/-/-) | **4.51×10^-7^ (9.37×10^-3^)** | - (-) | - (-) | - (-) |
| 10 | *CYP17A1* | - (-/-/-) | - (-/-/-) | **1.79×10^-8^ (1.74×10^-3^)** | - (-) | - (-) | - (-) |
| 10 | *AS3MT* | - (-/-/-) | - (-/-/-) | **7.27×10^-10^ (1.17×10^-5^)** | - (-) | - (-) | - (-) |
| 10 | *CNNM2* | - (-/-/-) | - (-/-/-) | **5.51×10^-8^ (8.26×10^-4^)** | - (-) | - (-) | - (-) |
| 10 | *NT5C2* | - (-/-/-) | - (-/-/-) | **1.84×10^-7^ (6.18×10^-4^)** | - (-) | - (-) | - (-) |
| 10 | ***INA*** | - (-/-/-) | - (-/-/-) | **1.62×10^-6^ (3.53×10^-8^)** | - (-) | - (-) | - (-) |
| 10 | ***PDCD11*** | - (-/-/-) | - (-/-/-) | **1.11×10^-7^ (8.22×10^-4^)** | - (-) | - (-) | - (-) |
| 11 | *KCNJ11* | - (-/-/-) | - (-/-/-) | - (-) | **1.45×10^-6^ (5.00×10^-8^)** | - (-) | - (-) |
| 11 | *BDNF* | **8.26×10^-12^ (0.02**/0.38/**6.96×10^-14^**) | - (-/-/-) | - (-) | - (-) | - (-) | - (-) |
| 11 | *BUD13* | - (-/-/-) | - (-/-/-) | - (-) | - (-) | **4.57×10^-9^ (5.00×10^-10^)** | - (-) |
| 11 | *ZPR1* | - (-/-/-) | - (-/-/-) | - (-) | - (-) | **1.75×10^-11^ (7.77×10^-16^)** | - (-) |
| 11 | ***APOA4*** | - (-/-/-) | - (-/-/-) | - (-) | - (-) | **1.95×10^-7^ (5.11×10^-7^)** | - (-) |
| 11 | *APOA1* | - (-/-/-) | - (-/-/-) | - (-) | - (-) | 7.18×10^-9^ (0.09) | - (-) |
| 11 | *SIK3* | - (-/-/-) | - (-/-/-) | - (-) | - (-) | **5.73×10^-10^ (0.02)** | - (-) |
| 11 | *PAFAH1B2* | - (-/-/-) | - (-/-/-) | - (-) | - (-) | **7.06×10^-8^ (9.68×10^-3^)** | - (-) |
| 11 | *TAGLN* | - (-/-/-) | - (-/-/-) | 2.21×10^-6^ (0.34) | - (-) | - (-) | - (-) |
| 11 | *PCSK7* | - (-/-/-) | - (-/-/-) | - (-) | - (-) | **1.45×10^-6^ (6.41×10^-3^)** | - (-) |
| 12 | *HOXC4* | - (-/-/-) | - (-/-/-) | **6.39×10^-7^ (1.23×10^-7^)** | - (-) | - (-) | - (-) |
| 12 | *ATP2B1* | - (-/-/-) | - (-/-/-) | **3.16×10^-15^ (5.00×10^-10^)** | - (-) | - (-) | - (-) |
| 12 | *C12orf42* | - (-/-/-) | - (-/-/-) | - (-) | **4.66×10^-8^ (0.01)** | - (-) | - (-) |
| 13 | *SLC7A1* | - (-/-/-) | - (-/-/-) | **2.61×10^-8^ (4.51×10^-6^)** | - (-) | - (-) | - (-) |
| 16 | *PKD1L3* | - (-/-/-) | - (-/-/-) | - (-) | - (-) | **1.40×10^-8^ (6.84×10^-7^)** | - (-) |
| 16 | ***HP*** | - (-/-/-) | - (-/-/-) | - (-) | - (-) | **1.09×10^-6^ (1.87×10^-6^)** | - (-) |
| 16 | *HPR* | - (-/-/-) | - (-/-/-) | - (-) | - (-) | **3.37×10^-7^ (1.56×10^-5^)** | - (-) |
| 16 | *PMFBP1* | - (-/-/-) | - (-/-/-) | - (-) | - (-) | **1.68×10^-6^ (1.11×10^-5^)** | - (-) |
| 17 | *HOXB4* | - (-/-/-) | - (-/-/-) | 1.06×10^-6^ (0.16) | - (-) | - (-) | - (-) |
| 18 | *GAREM* | 1.51×10^-6^ (0.95/0.10/0.07) | - (-/-/-) | - (-) | - (-) | - (-) | - (-) |
| 18 | *LIPG* | - (-/-/-) | - (-/-/-) | - (-) | - (-) | **1.06×10^-7^ (1.50×10^-4^)** | - (-) |
| 19 | *DOCK6* | - (-/-/-) | - (-/-/-) | - (-) | - (-) | **3.95×10^-7^ (4.16×10^-3^)** | - (-) |
| 19 | *TOMM40* | - (-/-/-) | - (-/-/-) | - (-) | - (-) | **4.77×10^-13^ (5.00×10^-10^)** | - (-) |
| 19 | *APOE* | - (-/-/-) | - (-/-/-) | - (-) | - (-) | **9.74×10^-11^ (5.00×10^-10^)** | - (-) |
| 19 | *GIPR* | **1.20×10^-10^ (**-/0.46/**2.33×10^-15^**) | - (-/-/-) | - (-) | - (-) | - (-) | - (-) |
| 19 | *QPCTL* | **4.48×10^-9^ (**-/-/**3.85×10^-8^**) | - (-/-/-) | - (-) | - (-) | - (-) | - (-) |
| 19 | *FBXO46* | **9.19×10^-8^ (**-/0.68/**4.50×10^-5^**) | - (-/-/-) | - (-) | - (-) | - (-) | - (-) |
| 19 | *ZC3H4* | **1.88×10^-7^ (**0.42/0.53/**1.79×10^-9^**) | - (-/-/-) | - (-) | - (-) | - (-) | - (-) |
| 20 | *GDAP1L1* | - (-/-/-) | - (-/-/-) | - (-) | **7.34×10^-8^ (7.72×10^-4^)** | - (-) | - (-) |
| 20 | *R3HDML* | - (-/-/-) | - (-/-/-) | - (-) | **8.41×10^-10^ (8.26×10^-3^)** | - (-) | - (-) |
| 20 | *HNF4A* | - (-/-/-) | - (-/-/-) | - (-) | **1.73×10^-8^ (1.05×10^-4^)** | - (-) | - (-) |
| 20 | *TAF4* | 1.58×10^-6^ (0.28/0.47/0.37) | - (-/-/-) | - (-) | - (-) | - (-) | - (-) |
| 22 | *PNPLA3* | - (-/-/-) | - (-/-/-) | - (-) | - (-) | - (-) | 5.22×10^-15^ (**3.50×10^-7^**) |
| 22 | *SAMM50* | - (-/-/-) | - (-/-/-) | - (-) | - (-) | - (-) | 1.13×10^-9^ (**7.99×10^-5^**) |
