# Supplementary material for: Association of Multiple-trait Polygenic Risk Score with Obesity and Cardiometabolic Diseases in Korean Population
Source: Genomics Proteomics Bioinformatics. 2025 Nov 9;23(5):qzaf102. doi: 10.1093/gpbjnl/qzaf102 (PMC12975335; doi:10.1093/gpbjnl/qzaf102)
Supplement: qzaf102_Supplementary_Data [file qzaf102_supplementary_data.zip › 13-Feb-2026_113933_qzaf102_Supplementary_Data_TableS12Revised.zip!/qzaf102_Supplementary_Data_TableS12Revised/Table S9.docx]

**Table S9** **Summary for gene-set enrichment analyses results for obesity-related diseases**

| **Disease** | **Method: database** | **Gene-set 1: obesity** | | | **Gene-set 2: obesity-related diseases** | | |
| --- | --- | --- | --- | --- | --- | --- | --- |
|  |  | **Pathway counts (Significant / Total)** | **Minimum**  ***P* value** | **Minimum**  **FDR** | **Pathway counts (Significant / Total)** | **Minimum**  ***P* value** | **Minimum**  **FDR** |
| HT | GSEA: Reactome | 0 / 151 | 0.06 | 0.38 | 0 / 121 | 0.07 | 0.67 |
|  | ORA1: GO | 0 / 111 | 1.06 × 10^-3^ | 0.21 | 0 / 437 | 2.18 × 10^-4^ | 0.09 |
|  | ORA2: KEGG | 0 / 2 | 2.53 × 10^-3^ | 0.16 | **3 / 46** | 1.01 × 10^-5^ | **6.74 × 10^-4^** |
| T2D | GSEA: Reactome | 0 / 144 | 0.11 | 0.56 | 0 / 10 | 0.66 | 0.83 |
|  | ORA1: GO | 0 / 163 | 6.74 × 10^-4^ | 0.16 | 0 / 87 | 2.32 × 10^-3^ | 0.06 |
|  | ORA2: KEGG | 0 / 49 | 3.28 × 10^-3^ | 0.20 | 0 / 2 | 0.03 | 0.06- |
| HL | GSEA: Reactome | 0/ 152 | 0.17 | 0.45- | 0 / 34 | 0.24 | 0.81- |
|  | ORA1: GO | 0 / 141 | 7.03 × 10^-3^ | 0.22- | **182 / 615** | 4.38 × 10^-10^ | **2.69 × 10^-7^** |
|  | ORA2: KEGG | 0 / 2 | 1.93 × 10^-3^ | 0.13 | **6 / 15** | 5.19 × 10^-8^ | **7.79 × 10^-7^** |
| FL | GSEA: Reactome | 0 / 151 | 0.09 | 0.44 | 0 / 9 | 1.00 | 1.00 |
|  | ORA1: GO | 0 / 111 | 1.06 × 10^-3^ | 0.21 | **50 / 50** | 1.47 × 10^-3^ | **0.02** |
|  | ORA2: KEGG | 0 / 2 | 2.53 × 10^-3^ | 0.16 | **2 / 2** | 0.01 | **0.02** |
