# Supplementary material for: Association of Multiple-trait Polygenic Risk Score with Obesity and Cardiometabolic Diseases in Korean Population
Source: Genomics Proteomics Bioinformatics. 2025 Nov 9;23(5):qzaf102. doi: 10.1093/gpbjnl/qzaf102 (PMC12975335; doi:10.1093/gpbjnl/qzaf102)
Supplement: qzaf102_Supplementary_Data [file qzaf102_supplementary_data.zip › 13-Feb-2026_113933_qzaf102_Supplementary_Data_TableS12Revised.zip!/qzaf102_Supplementary_Data_TableS12Revised/Table S10.docx]

**Table S10 Significant gene-sets identified through gene-set enrichment analyses of obesity-related diseases**

**A. Significant gene-sets associated with HT from of ORA2: KEGG**

| **ID** | **Category: Subcategory: Description** | **Gene-set** | **GeneRatio** | ***P* value** | **FDR** |
| --- | --- | --- | --- | --- | --- |
| hsa04925 | Organismal Systems: Endocrine system: Aldosterone synthesis and secretion | *CACNA1D, CYP11B2, KCNK3, ATP2B1* | 4/14 | 1.01×10^-5^ | 6.74×10^-4^ |
| hsa04934 | Human Diseases: Endocrine and metabolic disease: Cushing syndrome | *CYP17A1, CACNA1D, WNT2B, KCNK3* | 4/14 | 6.14×10^-5^ | 2.06×10^-3^ |
| hsa04927 | Organismal Systems: Endocrine system: Cortisol synthesis and secretion | *CYP17A1, CACNA1D, KCNK3* | 3/14 | 1.07×10^-4^ | 2.39×10^-3^ |

**B. Significant gene-sets associated with HL from ORA1: GO**

| **ID** | **Description** | **Gene-set** | **GeneRatio** | ***P* value** | **FDR** |
| --- | --- | --- | --- | --- | --- |
| GO:0034375 | high-density lipoprotein particle remodeling | *LIPG, APOE, APOA4, APOA1* | 4/14 | 4.38×10^-10^ | 2.69×10^-7^ |
| GO:0043691 | reverse cholesterol transport | *LIPG, APOE, APOA4, APOA1* | 4/14 | 8.90×10^-10^ | 2.74×10^-7^ |
| GO:0034368 | protein-lipid complex remodeling | *LIPG, APOE, APOA4, APOA1* | 4/14 | 7.48×10^-9^ | 1.15×10^-6^ |
| GO:0034369 | plasma lipoprotein particle remodeling | *LIPG, APOE, APOA4, APOA1* | 4/14 | 7.48×10^-9^ | 1.15×10^-6^ |
| GO:0034367 | protein-containing complex remodeling | *LIPG, APOE, APOA4, APOA1* | 4/14 | 9.56×10^-9^ | 1.18×10^-6^ |
| GO:1905918 | regulation of CoA-transferase activity | *APOE, APOA4, APOA1* | 3/14 | 3.82×10^-6^ | 3.67×10^-6^ |
| GO:0071827 | plasma lipoprotein particle organization | *LIPG, APOE, APOA4, APOA1* | 4/14 | 4.18×10^-6^ | 3.67×10^-6^ |
| GO:0071825 | protein-lipid complex organization | *LIPG, APOE, APOA4, APOA1* | 4/14 | 5.30×10^-6^ | 4.08×10^-6^ |
| GO:0034370 | triglyceride-rich lipoprotein particle remodeling | *APOE, APOA4, APOA1* | 3/14 | 6.99×10^-6^ | 4.30×10^-6^ |
| GO:0034372 | very-low-density lipoprotein particle remodeling | *APOE, APOA4, APOA1* | 3/14 | 6.99×10^-6^ | 4.30×10^-6^ |
| GO:0033700 | phospholipid efflux | *APOE, APOA4, APOA1* | 3/14 | 1.16×10^-7^ | 6.46×10^-6^ |
| GO:0032371 | regulation of sterol transport | *LIPG, APOE, APOA4, APOA1* | 4/14 | 2.43×10^-7^ | 1.14×10^-5^ |
| GO:0032374 | regulation of cholesterol transport | *LIPG, APOE, APOA4, APOA1* | 4/14 | 2.43×10^-7^ | 1.14×10^-5^ |
| GO:0034384 | high-density lipoprotein particle clearance | *LIPG, APOE, APOA1* | 3/14 | 2.59×10^-7^ | 1.14×10^-5^ |
| GO:0097006 | regulation of plasma lipoprotein particle levels | *LIPG, APOE, APOA4, APOA1* | 4/14 | 4.76×10^-7^ | 1.95×10^-5^ |
| GO:0042157 | lipoprotein metabolic process | *LIPG, APOE, APOA4, APOA1* | 4/14 | 7.54×10^-7^ | 2.90×10^-5^ |
| GO:0042632 | cholesterol homeostasis | *LIPG, APOE, APOA4, APOA1* | 4/14 | 8.80×10^-7^ | 2.99×10^-5^ |
| GO:0055092 | sterol homeostasis | *LIPG, APOE, APOA4, APOA1* | 4/14 | 9.14×10^-7^ | 2.99×10^-5^ |
| GO:0034377 | plasma lipoprotein particle assembly | *APOE, APOA4, APOA1* | 3/14 | 9.24×10^-7^ | 2.99×10^-5^ |
| GO:0065005 | protein-lipid complex assembly | *APOE, APOA4, APOA1* | 3/14 | 1.28×10^-6^ | 3.94×10^-5^ |
| GO:0030301 | cholesterol transport | *LIPG, APOE, APOA4, APOA1* | 4/14 | 1.60×10^-6^ | 4.68×10^-5^ |
| GO:0015918 | sterol transport | *LIPG, APOE, APOA4, APOA1* | 4/14 | 2.12×10^-6^ | 5.76×10^-5^ |
| GO:0032373 | positive regulation of sterol transport | *LIPG, APOE, APOA1* | 3/14 | 2.25×10^-6^ | 5.76×10^-5^ |
| GO:0032376 | positive regulation of cholesterol transport | *LIPG, APOE, APOA1* | 3/14 | 2.25×10^-6^ | 5.76×10^-5^ |
| GO:0008203 | cholesterol metabolic process | *APOE, APOA4, HMGCR, APOA1* | 4/14 | 3.34×10^-6^ | 8.22×10^-5^ |
| GO:0032368 | regulation of lipid transport | *LIPG, APOE, APOA4, APOA1* | 4/14 | 4.12×10^-6^ | 9.43×10^-5^ |
| GO:0016125 | sterol metabolic process | *APOE, APOA4, HMGCR, APOA1* | 4/14 | 4.44×10^-6^ | 9.43×10^-5^ |
| GO:1902652 | secondary alcohol metabolic process | *APOE, APOA4, HMGCR, APOA1* | 4/14 | 4.44×10^-6^ | 9.43×10^-5^ |
| GO:0055090 | acylglycerol homeostasis | *APOE, APOA4, APOA1* | 3/14 | 4.45×10^-6^ | 9.43×10^-5^ |
| GO:0055088 | lipid homeostasis | *LIPG, APOE, APOA4, APOA1* | 4/14 | 7.26×10^-6^ | 1.49×10^-4^ |
| GO:0034381 | plasma lipoprotein particle clearance | *LIPG, APOE, APOA1* | 3/14 | 8.19×10^-6^ | 1.62×10^-4^ |
| GO:1905952 | regulation of lipid localization | *LIPG, APOE, APOA4, APOA1* | 4/14 | 8.62×10^-6^ | 1.66×10^-4^ |
| GO:0006695 | cholesterol biosynthetic process | *APOE, HMGCR, APOA1* | 3/14 | 1.36×10^-5^ | 2.45×10^-4^ |
| GO:1902653 | secondary alcohol biosynthetic process | *APOE, HMGCR, APOA1* | 3/14 | 1.36×10^-5^ | 2.45×10^-4^ |
| GO:0016126 | sterol biosynthetic process | *APOE, HMGCR, APOA1* | 3/14 | 1.85×10^-5^ | 3.25×10^-4^ |
| GO:0033344 | cholesterol efflux | *APOE, APOA4, APOA1* | 3/14 | 2.01×10^-5^ | 3.43×10^-4^ |
| GO:0035023 | regulation of Rho protein signal transduction | *APOE, DOCK6, APOA1* | 3/14 | 2.94×10^-5^ | 4.88×10^-4^ |
| GO:1904729 | regulation of intestinal lipid absorption | *APOA4, APOA1* | 2/14 | 3.32×10^-5^ | 5.32×10^-4^ |
| GO:0032370 | positive regulation of lipid transport | *LIPG, APOE, APOA1* | 3/14 | 3.38×10^-5^ | 5.32×10^-4^ |
| GO:0034380 | high-density lipoprotein particle assembly | *APOE, APOA1* | 2/14 | 3.92×10^-5^ | 6.03×10^-4^ |
| GO:1904478 | regulation of intestinal absorption | *APOA4, APOA1* | 2/14 | 4.57×10^-5^ | 6.86×10^-4^ |
| GO:0098869 | cellular oxidant detoxification | *APOE, HP, APOA4* | 3/14 | 4.80×10^-5^ | 6.95×10^-4^ |
| GO:0015850 | organic hydroxy compound transport | *LIPG, APOE, APOA4, APOA1* | 4/14 | 4.86×10^-5^ | 6.95×10^-4^ |
| GO:2001140 | positive regulation of phospholipid transport | *APOE, APOA1* | 2/14 | 5.27×10^-5^ | 7.37×10^-4^ |
| GO:0015914 | phospholipid transport | *APOE, APOA4, APOA1* | 3/14 | 5.72×10^-5^ | 7.82×10^-4^ |
| GO:2001138 | regulation of phospholipid transport | *APOE, APOA1* | 2/14 | 6.02×10^-5^ | 8.05×10^-4^ |
| GO:0006641 | triglyceride metabolic process | *LIPG, APOE, APOA4* | 3/14 | 6.23×10^-5^ | 8.15×10^-4^ |
| GO:0032488 | Cdc42 protein signal transduction | *APOE, APOA1* | 2/14 | 6.82×10^-5^ | 8.56×10^-4^ |
| GO:0055091 | phospholipid homeostasis | *LIPG, APOA1* | 2/14 | 6.82×10^-5^ | 8.56×10^-4^ |
| GO:1905954 | positive regulation of lipid localization | *LIPG, APOE, APOA1* | 3/14 | 7.12×10^-5^ | 8.76×10^-4^ |
| GO:0030299 | intestinal cholesterol absorption | *APOA4, APOA1* | 2/14 | 7.67×10^-5^ | 9.25×10^-4^ |
| GO:0008202 | steroid metabolic process | *APOE, APOA4, HMGCR, APOA1* | 4/14 | 8.19×10^-5^ | 9.69×10^-4^ |
| GO:0090205 | positive regulation of cholesterol metabolic process | *APOE, APOA1* | 2/14 | 8.57×10^-5^ | 9.95×10^-4^ |
| GO:1900221 | regulation of amyloid-beta clearance | *APOE, HMGCR* | 2/14 | 9.52×10^-5^ | 1.08×10^-3^ |
| GO:1990748 | cellular detoxification | *APOE, HP, APOA4* | 3/14 | 9.62×10^-5^ | 1.08×10^-3^ |
| GO:0016042 | lipid catabolic process | *LIPG, APOE, APOA4, PAFAH1B2* | 4/14 | 9.93×10^-5^ | 1.09×10^-3^ |
| GO:0006066 | alcohol metabolic process | *APOE, APOA4, HMGCR, APOA1* | 4/14 | 1.13×10^-4^ | 1.20×10^-3^ |
| GO:0045834 | positive regulation of lipid metabolic process | *APOE, APOA4, APOA1* | 3/14 | 1.13×10^-4^ | 1.20×10^-3^ |
| GO:0044241 | lipid digestion | *APOA4, APOA1* | 2/14 | 1.16×10^-4^ | 1.21×10^-3^ |
| GO:0006639 | acylglycerol metabolic process | *LIPG, APOE, APOA4* | 3/14 | 1.21×10^-4^ | 1.22×10^-3^ |
| GO:0097237 | cellular response to toxic substance | *APOE, HP, APOA4* | 3/14 | 1.21×10^-4^ | 1.22×10^-3^ |
| GO:0006638 | neutral lipid metabolic process | *LIPG, APOE, APOA4* | 3/14 | 1.24×10^-4^ | 1.23×10^-3^ |
| GO:0007266 | Rho protein signal transduction | *APOE, DOCK6, APOA1* | 3/14 | 1.32×10^-4^ | 1.29×10^-3^ |
| GO:0098856 | intestinal lipid absorption | *APOA4, APOA1* | 2/14 | 1.38×10^-4^ | 1.33×10^-3^ |
| GO:0046486 | glycerolipid metabolic process | *LIPG, APOE, APOA4, APOA1* | 4/14 | 1.42×10^-4^ | 1.34×10^-3^ |
| GO:0046165 | alcohol biosynthetic process | *APOE, HMGCR, APOA1* | 3/14 | 1.59×10^-4^ | 1.48×10^-3^ |
| GO:0062013 | positive regulation of small molecule metabolic process | *APOE, APOA4, APOA1* | 3/14 | 1.75×10^-4^ | 1.59×10^-3^ |
| GO:0010875 | positive regulation of cholesterol efflux | *APOE, APOA1* | 2/14 | 1.75×10^-4^ | 1.59×10^-3^ |
| GO:0019433 | triglyceride catabolic process | *LIPG, APOA4* | 2/14 | 1.89×10^-4^ | 1.68×10^-3^ |
| GO:0098754 | detoxification | *APOE, HP, APOA4* | 3/14 | 2.27×10^-4^ | 1.99×10^-3^ |
| GO:0042744 | hydrogen peroxide catabolic process | *HP/ APOA4* | 2/14 | 2.32×10^-4^ | 2.01×10^-3^ |
| GO:0045940 | positive regulation of steroid metabolic process | *APOE, APOA1* | 2/14 | 2.47×10^-4^ | 2.11×10^-3^ |
| GO:0015748 | organophosphate ester transport | *APOE, APOA4, APOA1* | 3/14 | 2.51×10^-4^ | 2.12×10^-3^ |
| GO:0006869 | lipid transport | *LIPG, APOE, APOA4, APOA1* | 4/14 | 2.78×10^-4^ | 2.31×10^-3^ |
| GO:0031329 | regulation of cellular catabolic process | *APOE, HP, APOA4, PAFAH1B2* | 4/14 | 3.10×10^-4^ | 2.54×10^-3^ |
| GO:0006694 | steroid biosynthetic process | *APOE, HMGCR, APOA1* | 3/14 | 3.36×10^-4^ | 2.72×10^-3^ |
| GO:0046461 | neutral lipid catabolic process | *LIPG, APOA4* | 2/14 | 3.68×10^-4^ | 2.90×10^-3^ |
| GO:0046464 | acylglycerol catabolic process | *LIPG, APOA4* | 2/14 | 3.68×10^-4^ | 2.90×10^-3^ |
| GO:0000302 | response to reactive oxygen species | *APOE, HP, APOA4* | 3/14 | 3.85×10^-4^ | 3.00×10^-3^ |
| GO:0097242 | amyloid-beta clearance | *APOE, HMGCR* | 2/14 | 4.07×10^-4^ | 3.13×10^-3^ |
| GO:0044058 | regulation of digestive system process | *APOA4, APOA1* | 2/14 | 4.48×10^-4^ | 3.40×10^-3^ |
| GO:0050892 | intestinal absorption | *APOA4, APOA1* | 2/14 | 4.69×10^-4^ | 3.48×10^-3^ |
| GO:0070328 | triglyceride homeostasis | *APOE, APOA1* | 2/14 | 4.69×10^-4^ | 3.48×10^-3^ |
| GO:0071402 | cellular response to lipoprotein particle stimulus | *APOE, APOA1* | 2/14 | 4.91×10^-4^ | 3.59×10^-3^ |
| GO:0090181 | regulation of cholesterol metabolic process | *APOE, APOA1* | 2/14 | 5.13×10^-4^ | 3.67×10^-3^ |
| GO:0090207 | regulation of triglyceride metabolic process | *APOE/ APOA4* | 2/14 | 5.13×10^-4^ | 3.67×10^-3^ |
| GO:0031331 | positive regulation of cellular catabolic process | *APOE, APOA4, PAFAH1B2* | 3/14 | 6.16×10^-4^ | 4.36×10^-3^ |
| GO:0010874 | regulation of cholesterol efflux | *APOE, APOA1* | 2/14 | 7.07×10^-4^ | 4.94×10^-3^ |
| GO:0042743 | hydrogen peroxide metabolic process | *HP/ APOA4* | 2/14 | 7.33×10^-4^ | 5.07×10^-3^ |
| GO:1901617 | organic hydroxy compound biosynthetic process | *APOE, HMGCR, APOA1* | 3/14 | 7.81×10^-4^ | 5.34×10^-3^ |
| GO:0042158 | lipoprotein biosynthetic process | *APOE, APOA1* | 2/14 | 8.72×10^-4^ | 5.89×10^-3^ |
| GO:0009636 | response to toxic substance | *APOE, HP, APOA4* | 3/14 | 9.22×10^-4^ | 6.16×10^-3^ |
| GO:0046503 | glycerolipid catabolic process | *LIPG, APOA4* | 2/14 | 1.05×10^-3^ | 6.97×10^-3^ |
| GO:0051056 | regulation of small GTPase mediated signal transduction | *APOE, DOCK6, APOA1* | 3/14 | 1.25×10^-3^ | 8.17×10^-3^ |
| GO:0051347 | positive regulation of transferase activity | *APOE, APOA4, APOA1* | 3/14 | 1.30×10^-3^ | 8.40×10^-3^ |
| GO:0050709 | negative regulation of protein secretion | *APOE, HMGCR* | 2/14 | 1.39×10^-3^ | 8.94×10^-3^ |
| GO:0046470 | phosphatidylcholine metabolic process | *APOA4, APOA1* | 2/14 | 1.43×10^-3^ | 9.08×10^-3^ |
| GO:0019216 | regulation of lipid metabolic process | *APOE, APOA4, APOA1* | 3/14 | 1.85×10^-3^ | 0.01 |
| GO:0062012 | regulation of small molecule metabolic process | *APOE, APOA4, APOA1* | 3/14 | 1.91×10^-3^ | 0.01 |
| GO:0046889 | positive regulation of lipid biosynthetic process | *LIPG, APOA4* | 2/14 | 2.12×10^-3^ | 0.01 |
| GO:0019915 | lipid storage | *APOE, APOA1* | 2/14 | 2.26×10^-3^ | 0.01 |
| GO:0006644 | phospholipid metabolic process | *LIPG, APOA4, APOA1* | 3/14 | 2.35×10^-3^ | 0.01 |
| GO:0019218 | regulation of steroid metabolic process | *APOE, APOA1* | 2/14 | 2.89×10^-3^ | 0.02 |
| GO:0060291 | long-term synaptic potentiation | *APOE, HMGCR* | 2/14 | 2.89×10^-3^ | 0.02 |
| GO:0006979 | response to oxidative stress | *APOE, HP, APOA4* | 3/14 | 2.94×10^-3^ | 0.02 |
| GO:0022600 | digestive system process | *APOA4, APOA1* | 2/14 | 3.00×10^-3^ | 0.02 |
| GO:0051224 | negative regulation of protein transport | *APOE, HMGCR* | 2/14 | 3.49×10^-3^ | 0.02 |
| GO:0051223 | regulation of protein transport | *APOE, ZPR1, HMGCR* | 3/14 | 3.86×10^-3^ | 0.02 |
| GO:1904950 | negative regulation of establishment of protein localization | *APOE, HMGCR* | 2/14 | 4.38×10^-3^ | 0.02 |
| GO:0043603 | amide metabolic process | *APOE, PCSK7, HMGCR* | 3/14 | 4.53×10^-3^ | 0.03 |
| GO:0007586 | digestion | *APOA4, APOA1* | 2/14 | 4.90×10^-3^ | 0.03 |
| GO:0007264 | small GTPase-mediated signal transduction | *APOE, DOCK6, APOA1* | 3/14 | 5.06×10^-3^ | 0.03 |
| GO:1903531 | negative regulation of secretion by cell | *APOE, HMGCR* | 2/14 | 5.58×10^-3^ | 0.03 |
| GO:0045807 | positive regulation of endocytosis | *APOE, APOA1* | 2/14 | 5.94×10^-3^ | 0.03 |
| GO:0006633 | fatty acid biosynthetic process | *LIPG, APOA4* | 2/14 | 6.38×10^-3^ | 0.03 |
| GO:0051048 | negative regulation of secretion | *APOE, HMGCR* | 2/14 | 7.15×10^-3^ | 0.04 |
| GO:0050806 | positive regulation of synaptic transmission | *APOE, HMGCR* | 2/14 | 7.23×10^-3^ | 0.04 |
| GO:1902905 | positive regulation of supramolecular fiber organization | *APOE, APOA1* | 2/14 | 7.31×10^-3^ | 0.04 |
| GO:0014012 | peripheral nervous system axon regeneration | *APOA4* | 1/14 | 7.35×10^-3^ | 0.04 |
| GO:0018158 | protein oxidation | *APOA1* | 1/14 | 7.35×10^-3^ | 0.04 |
| GO:0038060 | nitric oxide-cGMP-mediated signaling | *APOE* | 1/14 | 7.35×10^-3^ | 0.04 |
| GO:0045541 | negative regulation of cholesterol biosynthetic process | *APOE* | 1/14 | 7.35×10^-3^ | 0.04 |
| GO:0097113 | AMPA glutamate receptor clustering | *APOE* | 1/14 | 7.35×10^-3^ | 0.04 |
| GO:0097688 | glutamate receptor clustering | *APOE* | 1/14 | 7.35×10^-3^ | 0.04 |
| GO:0106119 | negative regulation of sterol biosynthetic process | *APOE* | 1/14 | 7.35×10^-3^ | 0.04 |
| GO:2000822 | regulation of behavioral fear response | *APOE* | 1/14 | 7.35×10^-3^ | 0.04 |
| GO:0043534 | blood vessel endothelial cell migration | *APOE, APOA1* | 2/14 | 7.71×10^-3^ | 0.04 |
| GO:0010982 | regulation of high-density lipoprotein particle clearance | *LIPG* | 1/14 | 8.08×10^-3^ | 0.04 |
| GO:0010986 | positive regulation of lipoprotein particle clearance | *LIPG* | 1/14 | 8.08×10^-3^ | 0.04 |
| GO:0034115 | negative regulation of heterotypic cell-cell adhesion | *APOA1* | 1/14 | 8.08×10^-3^ | 0.04 |
| GO:0070587 | regulation of cell-cell adhesion involved in gastrulation | *APOA1* | 1/14 | 8.08×10^-3^ | 0.04 |
| GO:0090206 | negative regulation of cholesterol metabolic process | *APOE* | 1/14 | 8.08×10^-3^ | 0.04 |
| GO:1900222 | negative regulation of amyloid-beta clearance | *HMGCR* | 1/14 | 8.08×10^-3^ | 0.04 |
| GO:1903365 | regulation of fear response | *APOE* | 1/14 | 8.08×10^-3^ | 0.04 |
| GO:2000672 | negative regulation of motor neuron apoptotic process | *ZPR1* | 1/14 | 8.08×10^-3^ | 0.04 |
| GO:0017038 | protein import | *APOE* | 1/14 | 8.82×10^-3^ | 0.04 |
| GO:0070586 | cell-cell adhesion involved in gastrulation | *APOA1* | 1/14 | 8.82×10^-3^ | 0.04 |
| GO:0097090 | presynaptic membrane organization | *APOE* | 1/14 | 8.82×10^-3^ | 0.04 |
| GO:2000644 | regulation of receptor catabolic process | *APOE* | 1/14 | 8.82×10^-3^ | 0.04 |
| GO:0046890 | regulation of lipid biosynthetic process | *APOE, APOA1* | 2/14 | 8.89×10^-3^ | 0.04 |
| GO:0001935 | endothelial cell proliferation | *APOE, APOA1* | 2/14 | 9.42×10^-3^ | 0.04 |
| GO:0050728 | negative regulation of inflammatory response | *APOE, APOA1* | 2/14 | 9.51×10^-3^ | 0.04 |
| GO:0010896 | regulation of triglyceride catabolic process | *APOA4* | 1/14 | 9.55×10^-3^ | 0.04 |
| GO:0038203 | TORC2 signaling | *SIK3* | 1/14 | 9.55×10^-3^ | 0.04 |
| GO:0097475 | motor neuron migration | *CELSR2* | 1/14 | 9.55×10^-3^ | 0.04 |
| GO:0007008 | outer mitochondrial membrane organization | *TOMM40* | 1/14 | 0.01 | 0.04 |
| GO:0045040 | protein insertion into mitochondrial outer membrane | *TOMM40* | 1/14 | 0.01 | 0.04 |
| GO:0051004 | regulation of lipoprotein lipase activity | *APOA4* | 1/14 | 0.01 | 0.04 |
| GO:0060354 | negative regulation of cell adhesion molecule production | *APOA1* | 1/14 | 0.01 | 0.04 |
| GO:0061000 | negative regulation of dendritic spine development | *APOE* | 1/14 | 0.01 | 0.04 |
| GO:1900272 | negative regulation of long-term synaptic potentiation | *APOE* | 1/14 | 0.01 | 0.04 |
| GO:1902950 | regulation of dendritic spine maintenance | *APOE* | 1/14 | 0.01 | 0.04 |
| GO:1905809 | negative regulation of synapse organization | *APOE* | 1/14 | 0.01 | 0.04 |
| GO:1905907 | negative regulation of amyloid fibril formation | *APOE* | 1/14 | 0.01 | 0.04 |
| GO:0001833 | inner cell mass cell proliferation | *ZPR1* | 1/14 | 0.01 | 0.04 |
| GO:2000671 | regulation of motor neuron apoptotic process | *ZPR1* | 1/14 | 0.01 | 0.04 |
| GO:0048167 | regulation of synaptic plasticity | *APOE, HMGCR* | 2/14 | 0.01 | 0.04 |
| GO:1903828 | negative regulation of protein localization | *APOE, HMGCR* | 2/14 | 0.01 | 0.04 |
| GO:0072330 | monocarboxylic acid biosynthetic process | *LIPG, APOA4* | 2/14 | 0.01 | 0.04 |
| GO:0034374 | low-density lipoprotein particle remodeling | *APOE* | 1/14 | 0.01 | 0.04 |
| GO:0035641 | locomotory exploration behavior | *APOE* | 1/14 | 0.01 | 0.04 |
| GO:0043117 | positive regulation of vascular permeability | *APOE* | 1/14 | 0.01 | 0.05 |
| GO:0050746 | regulation of lipoprotein metabolic process | *LIPG* | 1/14 | 0.01 | 0.05 |
| GO:0051044 | positive regulation of membrane protein ectodomain proteolysis | *APOE* | 1/14 | 0.01 | 0.05 |
| GO:0055089 | fatty acid homeostasis | *APOE* | 1/14 | 0.01 | 0.05 |
| GO:0090209 | negative regulation of triglyceride metabolic process | *APOE* | 1/14 | 0.01 | 0.05 |
| GO:0044242 | cellular lipid catabolic process | *LIPG, APOA4* | 2/14 | 0.01 | 0.05 |
| GO:0016358 | dendrite development | *CELSR2, APOE* | 2/14 | 0.01 | 0.05 |
| GO:0010310 | regulation of hydrogen peroxide metabolic process | *HP* | 1/14 | 0.01 | 0.05 |
| GO:0010544 | negative regulation of platelet activation | *APOE* | 1/14 | 0.01 | 0.05 |
| GO:0015936 | coenzyme A metabolic process | *HMGCR* | 1/14 | 0.01 | 0.05 |
| GO:0042159 | lipoprotein catabolic process | *APOE* | 1/14 | 0.01 | 0.05 |
| GO:0072578 | neurotransmitter-gated ion channel clustering | *APOE* | 1/14 | 0.01 | 0.05 |
| GO:0090083 | regulation of inclusion body assembly | *APOE* | 1/14 | 0.01 | 0.05 |
| GO:1905906 | regulation of amyloid fibril formation | *APOE* | 1/14 | 0.01 | 0.05 |
| GO:0072593 | reactive oxygen species metabolic process | *HP, APOA4* | 2/14 | 0.01 | 0.05 |
| GO:0030575 | nuclear body organization | *ZPR1* | 1/14 | 0.01 | 0.05 |
| GO:1902931 | negative regulation of alcohol biosynthetic process | *APOE* | 1/14 | 0.01 | 0.05 |
| GO:0030150 | protein import into mitochondrial matrix | *TOMM40* | 1/14 | 0.01 | 0.05 |
| GO:0033194 | response to hydroperoxide | *APOA4* | 1/14 | 0.01 | 0.05 |
| GO:0043116 | negative regulation of vascular permeability | *APOE* | 1/14 | 0.01 | 0.05 |
| GO:0060353 | regulation of cell adhesion molecule production | *APOA1* | 1/14 | 0.01 | 0.05 |

**C. Significant gene-sets associated with HL from ORA2: KEGG**

| **ID** | **Category: Subcategory: Description** | **Gene-set** | **GeneRatio** | ***P* value** | **FDR** |
| --- | --- | --- | --- | --- | --- |
| hsa04979 | Organismal Systems: Digestive system: Cholesterol metabolism | *LIPG, APOE, APOA4, APOA1* | 4/8 | 5.19×10^-8^ | 7.79×10^-7^ |
| hsa04977 | Organismal Systems: Digestive system: Vitamin digestion and absorption | *LIPG, APOE, APOA4, APOA1* | 2/8 | 2.02×10^-4^ | 1.51×10^-3^ |
| hsa05143 | Human Diseases: Infectious disease: parasitic African trypanosomiasis | *LIPG, APOE, APOA4, APOA1* | 2/8 | 4.12×10^-4^ | 2.06×10^-3^ |
| hsa04975 | Organismal Systems: Digestive system: Fat digestion and absorption | *LIPG, APOE, APOA4, APOA1* | 2/8 | 5.57×10^-4^ | 2.09×10^-3^ |
| hsa05417 | Human Diseases: Cardiovascular disease: Lipid and atherosclerosis | *LIPG, APOE, APOA4, APOA1* | 2/8 | 0.13 | 0.04 |
| hsa00900 | Metabolism: Metabolism of terpenoids and polyketides: T erpenoid backbone biosynthesis | *APOE, APOA4, APOA1* | 1/8 | 0.02 | 0.05 |

**D. Significant gene-sets associated with FL from ORA1: GO**

| **ID** | **Description** | **Gene-set** | **GeneRatio** | ***P* value** | **FDR** |
| --- | --- | --- | --- | --- | --- |
| GO:0007008 | outer mitochondrial membrane organization | *SAMM50* | 1/2 | 1.47×10^-3^ | 0.02 |
| GO:0045040 | protein insertion into mitochondrial outer membrane | *SAMM50* | 1/2 | 1.47×10^-3^ | 0.02 |
| GO:0050872 | white fat cell differentiation | *PNPLA3* | 1/2 | 1.79×10^-3^ | 0.02 |
| GO:0042407 | cristae formation | *SAMM50* | 1/2 | 2.11×10^-3^ | 0.02 |
| GO:0019433 | triglyceride catabolic process | *PNPLA3* | 1/2 | 2.95×10^-3^ | 0.02 |
| GO:0006654 | phosphatidic acid biosynthetic process | *PNPLA3* | 1/2 | 3.58×10^-3^ | 0.02 |
| GO:0090151 | establishment of protein localization to mitochondrial membrane | *SAMM50* | 1/2 | 3.57×10^-3^ | 0.02 |
| GO:0046473 | phosphatidic acid metabolic process | *PNPLA3* | 1/2 | 4.00×10^-3^ | 0.02 |
| GO:0034389 | lipid droplet organization | *PNPLA3* | 1/2 | 4.10×10^-3^ | 0.02 |
| GO:0046461 | neutral lipid catabolic process | *PNPLA3* | 1/2 | 4.10×10^-3^ | 0.02 |
| GO:0046464 | acylglycerol catabolic process | *PNPLA3* | 1/2 | 4.10×10^-3^ | 0.02 |
| GO:0019432 | triglyceride biosynthetic process | *PNPLA3* | 1/2 | 4.63×10^-3^ | 0.02 |
| GO:0007007 | inner mitochondrial membrane organization | *SAMM50* | 1/2 | 4.74×10^-3^ | 0.02 |
| GO:0046460 | neutral lipid biosynthetic process | *PNPLA3* | 1/2 | 5.58×10^-3^ | 0.02 |
| GO:0046463 | acylglycerol biosynthetic process | *PNPLA3* | 1/2 | 5.58×10^-3^ | 0.02 |
| GO:0046503 | glycerolipid catabolic process | *PNPLA3* | 1/2 | 6.94×10^-3^ | 0.02 |
| GO:0071230 | cellular response to amino acid stimulus | *PNPLA3* | 1/2 | 9.56×10^-3^ | 0.03 |
| GO:0071229 | cellular response to acid chemical | *PNPLA3* | 1/2 | 0.01 | 0.03 |
| GO:0033108 | mitochondrial respiratory chain complex assembly | *SAMM50* | 1/2 | 0.01 | 0.03 |
| GO:0006626 | protein targeting to mitochondrion | *SAMM50* | 1/2 | 0.01 | 0.03 |
| GO:0006641 | triglyceride metabolic process | *PNPLA3* | 1/2 | 0.01 | 0.03 |
| GO:0001676 | long-chain fatty acid metabolic process | *PNPLA3* | 1/2 | 0.01 | 0.03 |
| GO:0007006 | mitochondrial membrane organization | *SAMM50* | 1/2 | 0.01 | 0.03 |
| GO:0072655 | establishment of protein localization to mitochondrion | *SAMM50* | 1/2 | 0.01 | 0.03 |
| GO:0043200 | response to amino acid | *PNPLA3* | 1/2 | 0.01 | 0.03 |
| GO:0070585 | protein localization to mitochondrion | *SAMM50* | 1/2 | 0.01 | 0.03 |
| GO:0006639 | acylglycerol metabolic process | *PNPLA3* | 1/2 | 0.01 | 0.03 |
| GO:0006638 | neutral lipid metabolic process | *PNPLA3* | 1/2 | 0.01 | 0.03 |
| GO:0001101 | response to acid chemical | *PNPLA3* | 1/2 | 0.02 | 0.03 |
| GO:0055088 | lipid homeostasis | *PNPLA3* | 1/2 | 0.02 | 0.03 |
| GO:0006839 | mitochondrial transport | *SAMM50* | 1/2 | 0.02 | 0.03 |
| GO:0046474 | glycerophospholipid biosynthetic process | *PNPLA3* | 1/2 | 0.02 | 0.03 |
| GO:0032869 | cellular response to insulin stimulus | *PNPLA3* | 1/2 | 0.02 | 0.03 |
| GO:0044242 | cellular lipid catabolic process | *PNPLA3* | 1/2 | 0.02 | 0.03 |
| GO:0045017 | glycerolipid biosynthetic process | *PNPLA3* | 1/2 | 0.03 | 0.03 |
| GO:0008654 | phospholipid biosynthetic process | *PNPLA3* | 1/2 | 0.03 | 0.03 |
| GO:0009743 | response to carbohydrate | *PNPLA3* | 1/2 | 0.03 | 0.03 |
| GO:0045444 | fat cell differentiation | *PNPLA3* | 1/2 | 0.03 | 0.03 |
| GO:0032868 | response to insulin | *PNPLA3* | 1/2 | 0.03 | 0.04 |
| GO:0090150 | establishment of protein localization to membrane | *SAMM50* | 1/2 | 0.03 | 0.04 |
| GO:0006650 | glycerophospholipid metabolic process | *PNPLA3* | 1/2 | 0.03 | 0.04 |
| GO:0071375 | cellular response to peptide hormone stimulus | *PNPLA3* | 1/2 | 0.03 | 0.04 |
| GO:0006605 | protein targeting | *SAMM50* | 1/2 | 0.03 | 0.04 |
| GO:0016042 | lipid catabolic process | *PNPLA3* | 1/2 | 0.04 | 0.04 |
| GO:0006644 | phospholipid metabolic process | *PNPLA3* | 1/2 | 0.04 | 0.04 |
| GO:0046486 | glycerolipid metabolic process | *PNPLA3* | 1/2 | 0.04 | 0.04 |
| GO:0006631 | fatty acid metabolic process | *PNPLA3* | 1/2 | 0.04 | 0.04 |
| GO:0043434 | response to peptide hormone | *PNPLA3* | 1/2 | 0.05 | 0.05 |
| GO:0007005 | mitochondrion organization | *SAMM50* | 1/2 | 0.05 | 0.05 |
| GO:0072594 | establishment of protein localization to organelle | *SAMM50* | 1/2 | 0.05 | 0.05 |

**E. Significant gene-sets associated with FL from ORA2: KEGG**

| **ID** | **Category: Subcategory: Description** | **Gene-set** | **GeneRatio** | ***P* value** | **FDR** |
| --- | --- | --- | --- | --- | --- |
| hsa00561 | Metabolism: Lipid metabolism: Glycerolipid metabolism | *PNPLA3* | 1/2 | 0.01 | 0.02 |
| hsa04137 | Cellular Processes: Transport and catabolism: Mitophagy - animal | *SAMM50* | 1/2 | 0.02 | 0.02 |
