# Supplementary material for: Association of Multiple-trait Polygenic Risk Score with Obesity and Cardiometabolic Diseases in Korean Population
Source: Genomics Proteomics Bioinformatics. 2025 Nov 9;23(5):qzaf102. doi: 10.1093/gpbjnl/qzaf102 (PMC12975335; doi:10.1093/gpbjnl/qzaf102)
Supplement: qzaf102_Supplementary_Data [file qzaf102_supplementary_data.zip › 13-Feb-2026_113933_qzaf102_Supplementary_Data_TableS12Revised.zip!/qzaf102_Supplementary_Data_TableS12Revised/Table S11.docx]

**Table S11 Distribution of obesity and obesity-related diseases in samples used for PRS analyses**

| **Trait** | **Validation dataset** | | | **Test dataset** | | |
| --- | --- | --- | --- | --- | --- | --- |
|  | **Prevalence (%)** | **Control** | **Case** | **Prevalence (%)** | **Control** | **Case** |
| BMI25 | 32.44 % | 10,132 | 4864 | 33.48 % | 46,411 | 23,355 |
| BMI30 | 2.94 % | 14,555 | 441 | 2.97 % | 67,696 | 2070 |
| WC1 | 25.15 % | 10,274 | 3453 | 25.56 % | 48,672 | 16,714 |
| WC2 | 3.72 % | 13,217 | 510 | 3.67 % | 62,985 | 2401 |
| HT | 22.65 % | 9769 | 2860 | 22.31 % | 47,122 | 13,529 |
| T2D | 7.3 % | 12,454 | 981 | 7.84 % | 58,953 | 5015 |
| CVD | 4.31 % | 11,878 | 535 | 4.68 % | 56,935 | 2793 |
| HP | 12.36 % | 10,992 | 1550 | 11.31 % | 53,432 | 6816 |
| FL | 5.7 % | 10,711 | 648 | 5.8 % | 46,742 | 2877 |
