# Supplementary material for: Association of Multiple-trait Polygenic Risk Score with Obesity and Cardiometabolic Diseases in Korean Population
Source: Genomics Proteomics Bioinformatics. 2025 Nov 9;23(5):qzaf102. doi: 10.1093/gpbjnl/qzaf102 (PMC12975335; doi:10.1093/gpbjnl/qzaf102)
Supplement: qzaf102_Supplementary_Data [file qzaf102_supplementary_data.zip › 13-Feb-2026_113933_qzaf102_Supplementary_Data_TableS12Revised.zip!/qzaf102_Supplementary_Data_TableS12Revised/Table S12_Revised.docx]

**Table S12 Heritability analyses and genetic correlations for PRS target traits in EAS populations**

**A. SNP heritability estimates and genetic correlations in EAS populations**

|  | **SBP** | **DBP** | **BS** | **TG** | **HDL** | **BMI** | **WC** |
| --- | --- | --- | --- | --- | --- | --- | --- |
| SBP | **0.10** (1.88×10^-23^) | **0.93** (0) | **0.26** (1.12×10^-5^) | **0.25** (1.11×10^-18^) | **-0.09** (1.11×10^-7^) | 0.29 (0.12) | **0.33** (1.80×10^-8^) |
|  | **0.08** (2.03×10^-30^) | **0.86** (0) | **0.21** (9.21×10^-4^) | **0.15** (2.00×10^-3^) | -0.11 (0.29) | **0.28** (1.24×10^-14^) | **-** |
|  | **0.14** (1.90×10^-46^) | **0.86** (0) | **0.18 (**1.33×10^-5^) | **0.23** (9.69×10^-13^) | **-0.20** (1.37×10^-7^) | **0.28** (1.21×10^-12^) | **0.32** (3.96×10^-12^) |
| DBP |  | **0.09** (1.04×10^-25^) | 0.12 (0.06) | **0.20** (1.64×10^-5^) | -0.09 (0.10) | **0.23** (4.24×10^-7^) | **0.28** (8.39×10^-8^) |
|  |  | **0.06** (1.24×10^-22^) | 0.13 (0.09) | 0.07 (0.14) | -0.01 (0.94) | **0.28** (2.21×10^-14^) | ***-*** |
|  |  | **0.14** (7.65×10^-53^) | **0.10** (0.02) | **0.21** (7.66×10^-9^) | **-0.19** (1.06×10^-6^) | **0.26** (2.30×10^-10^) | **0.29** (1.10×10^-11^) |
| BS |  |  | **0.12** (1.11×10^-18^) | 0.16 (0.06) | **-0.20** (4.00×10^-3^) | **0.20** (3.57×10^-5^) | **0.31** (7.72×10^-9^) |
|  |  |  | **0.05** (1.56×10^-12^) | 0.07 (0.31) | -0.08 (0.44) | **0.09** (0.08) | ***-*** |
|  |  |  | **0.15** (8.81×10^-18^) | **0.24** (4.00×10^-4^) | **-0.20** (1.00×10^-4^) | **0.22** (3.39×10^-6^) | **0.26** (7.26×10^-7^) |
| TG |  |  |  | **0.16** (1.91×10^-7^) | **-0.61** (2.10×10^-16^) | **0.21** (4.31×10^-6^) | **0.27** (4.87×10^-8^) |
|  |  |  |  | **0.12** (4.16×10^-5^) | **-0.72** (1.10×10^-8^) | **0.20** (2.75×10^-4^) | ***-*** |
|  |  |  |  | **0.20** (3.50×10^-7^) | **-0.61** (9.00×10^-18^) | **0.26** (2.76×10^-6^) | **0.27** (1.21×10^-6^) |
| HDL |  |  |  |  | **0.17** (8.70×10^-15^) | **-0.23** (1.22×10^-7^) | **-0.27** (6.70×10^-7^) |
|  |  |  |  |  | **0.16** (1.28×10^-8^) | **-0.27** (1.88×10^-3^) | ***-*** |
|  |  |  |  |  | **0.25** (6.04×10^-19^) | **-0.40** (1.66×10^-12^) | **-0.39** (1.09×10^-10^) |
| BMI |  |  |  |  |  | **0.21** (1.07×10^-62^) | **0.86** (0) |
|  |  |  |  |  |  | **0.17** (1.07×10^-101^) | ***-*** |
|  |  |  |  |  |  | **0.22** (1.69×10^-99^) | **0.88** (0) |
| WC |  |  |  |  |  |  | **0.15** (3.66×10^-46^) |
|  |  |  |  |  |  |  | **-** |
|  |  |  |  |  |  |  | **0.16** (1.12×10^-69^) |

**B. Estimated genetic correlations between Japanese/Chinese and Korean populations**

| **Trait** | **SBP** | **DBP** | **BS** | **TG** | **HDL** | **BMI** | **WC** |
| --- | --- | --- | --- | --- | --- | --- | --- |
| *GC_BBJ-KOR_* | **0.99**  (P = 3.37 × 10^-68^) | **1.02**  (P = 2.77 × 10^-48^) | **0.89**  (P = 2.88 × 10^-30^) | **0.90**  (P = 1.69 × 10^-109^) | **0.95**  (P = 5.25 × 10^-56^) | **0.90**  (P = 0) | - |
| *GC_TWB-KOR_* | **0.99**  (P = 3.35 × 10^-65^) | **0.95**  (P = 1.94 × 10^-66^) | **1.00**  (P = 1.52 × 10^-90^) | **0.91**  (P = 7.76 × 10^-115^) | **0.89**  (P = 3.37 × 10^-85^) | **0.92**  (P = 1.23 × 10^-243^) | **0.94**  (P = 3.59 × 10^-128^) |

*Note*: In table A, diagonal elements denote SNP heritability estimates (*h^2^_KOR_*, *h^2^_BBJ_* and *h^2^_TWB_*) and upper diagonal elements denote genetic correlations (*GC_KOR_*, *GC_BBJ_*, and *GC_TWB_*) estimated from Korean, Japanese (BBJ), and Chinese (TWB) populations; In table B, *GC_BBJ-KOR_* and *GC_TWB-KOR_* represent the genetic correlations estimated between BBJ/TWB and Korean population.
