# Supplementary material for: Association of Multiple-trait Polygenic Risk Score with Obesity and Cardiometabolic Diseases in Korean Population
Source: Genomics Proteomics Bioinformatics. 2025 Nov 9;23(5):qzaf102. doi: 10.1093/gpbjnl/qzaf102 (PMC12975335; doi:10.1093/gpbjnl/qzaf102)
Supplement: qzaf102_Supplementary_Data [file qzaf102_supplementary_data.zip › 13-Feb-2026_113935_qzaf102_Supplementary_Data_TableSRevised.zip!/qzaf102_Supplementary_Data_TableSRevised/Table S7.docx]

**Table S7**  **Significant genetic regions identified by local genetic correlations between obesity and obesity-related diseases**

**A. Significant local genetic correlations between general obesity and obesity-related diseases**

| **Disease** | **Discovery dataset** | | | | **Replication dataset of REP3_NHW_** | | | |  |
| --- | --- | --- | --- | --- | --- | --- | --- | --- | --- |
|  | **Region** | **GC**  **(*P* value)** | **BMI25** $\boldsymbol{h}^{\boldsymbol{2}}$  **(*P* value)** | **Disease** $\boldsymbol{h}^{\boldsymbol{2}}$  **(*P* value)** | **Region** | **GC**  **(*P* value)** | **BMI30** $\boldsymbol{h}^{\boldsymbol{2}}$  **(*P* value)** | **Disease** $\boldsymbol{h}^{\boldsymbol{2}}$  **(*P* value)** |  |
| HT | 2:215569986–217243305 | 1.00  (1.56×10^-6^) | 1.31×10^-3^ (3.12×10^-5^) | 1.66×10^-3^ (5.67×10^-5^) | 2:215899571–217566011 | −0.33  (0.10) | 2.06×10^-4^  (1.85×10^-3^) | 5.20×10^-4^  (2.90×10^-11^) |  |
| **HT** | **3:8327718**–**9760484** | 0.97  (2.56×10^-5^) | 1.09×10^-3^ (3.10×10^-4^) | 1.41×10^-3^ (5.39×10^-5^) | **3:8664893**–**9970731** | **0.52**  **(8.23×10^-4^)** | 5.34×10^-4^  (7.45×10^-12^) | 3.82×10^-4^  (3.75×10^-7^) |  |
| HT | 3:51817883–53541920 | 0.82  (9.38×10^-6^) | 1.34×10^-3^ (4.61×10^-6^) | 1.76×10^-3^ (2.08×10^-7^) | 3:51953969–54074844 | 0.13  (0.25) | 5.09×10^-4^  (3.31×10^-11^) | 8.46×10^-4^  (1.43×10^-24^) |  |
| HT | 7:1355243–2477957 | 0.61  (2.77×10^-5^) | 1.52×10^-3^ (2.94×10^-7^) | 3.19×10^-3^ (2.70×10^-15^) | 7:1366973–2473749 | 0.15  (0.26) | 6.64×10^-4^  (2.02×10^-17^) | 4.54×10^-4^  (8.27×10^-10^) |  |
| HT | 11:6882946–7618719 | 1.00  (3.56×10^-5^) | 6.25×10^-3^ (7.67×10^-3^) | 1.84×10^-3^ (2.13×10^-7^) | 11:7191231–7915071 | −0.23  (0.35) | 3.20×10^-4^  (5.76×10^-7^) | 1.53×10^-4^  (6.30×10^-3^) |  |
| **HT** | **12:109020532**–**110116508** | 0.82  (6.17×10^-7^) | 1.55×10^-3^ (7.78×10^-8^) | 2.01×10^-3^ (1.35×10^-9^) | **12:109031820**–**110111514** | −**0.63**  **(0.02)** | 9.65×10^-5^  (0.05) | 4.00×10^-4^  (1.23×10^-9^) |  |
| HT | 12:110116509–112206126 | 0.70  (4.25×10^-6^) | 1.23×10^-3^  (1.08×10^-5^) | 3.42×10^-3^ (9.36×10^-20^) | 12:110111515–111592381 | 0.09  (0.61) | 2.71×10^-4^  (8.15×10^-7^) | 3.99×10^-4^  (2.59×10^-11^) |  |
| HT | 13:27952091–29255849 | 1.00  (2.33×10^-5^) | 1.63×10^-3^  (2.08×10^-7^) | 6.82×10^-4^ (0.02) | 13:27392006–28587125 | 0.06  (0.68) | 5.85×10^-4^  (9.64×10^-15^) | 4.18×10^-4^  (7.87×10^-9^) |  |
| HT | 14:20714628–21982816 | 0.54  (1.49×10^-5^) | 3.05×10^-3^ (1.29×10^-17^) | 2.57×10^-3^ (1.70×10^-11^) | 14:20876081–21982654 | 0.13  (0.45) | 2.94×10^-4^  (3.42×10^-5^) | 5.06×10^-4^  (5.54×10^-11^) |  |
| HT | 15:25803835–26656298 | 0.46  (2.62×10^-6^) | 3.02×10^-3^ (4.82×10^-19^) | 5.04×10^-3^ (5.21×10^-31^) | 15:25384328–26392947 | 0.12  (0.61) | 3.83×10^-4^  (1.56×10^-7^) | 1.84×10^-4^  (4.32×10^-3^) |  |
| HT | 16:5782847–6446996 | 0.81  (1.42×10^-6^) | 2.20×10^-3^ (1.60×10^-10^) | 1.74×10^-3^ (3.57×10^-6^) | 16:5782969–6446081 | 0.02  (0.87) | 6.35×10^-4^  (1.88×10^-17^) | 7.06×10^-4^  (3.43×10^-19^) |  |
| HT | 22:48274208–48979272 | 0.96  (1.81×10^-5^) | 1.66×10^-3^  (3.11×10^-7^) | 9.49×10^-4^ (2.11×10^-3^) | 22:48269178–48977538 | 0.05  (0.74) | 5.18×10^-5^  (1.13×10^-12^) | 3.83×10^-4^  (5.25×10^-8^) |  |
| T2D | 1:183930245–184982129 | 1.00  (1.39×10^-6^) | 1.74×10^-3^ (6.36×10^-9^) | 5.73×10^-4^ (0.02) | 1:183920617–184953089 | 0.43  (0.08) | 3.98×10^-4^  (1.27×10^-9^) | 1.55×10^-4^  (0.02) |  |
| T2D | 3:76161795–77157986 | 0.87  (8.39×10^-6^) | 1.25×10^-3^ (1.19×10^-5^) | 9.51×10^-4^ (2.07×10^-6^) | 3:76157471–77507832 | −0.39  (0.24) | 1.89×10^-4^  (1.29×10^-3^) | 1.50×10^-4^  (0.02) |  |
| T2D | 10:68374233–69909446 | 0.96  (3.03×10^-6^) | 1.24×10^-3^  (1.82×10^-5^) | 1.12×10^-3^  (1.04×10^-5^) | 10:69904171–71050504 | −0.08  (0.76) | 1.79×10^-4^  (1.42×10^-3^) | 2.96×10^-4^  (8.61×10^-5^) |  |
| T2D | 12:110116509–112206126 | 0.79  (3.84×10^-7^) | 1.23×10^-3^  (1.08×10^-5^) | 2.79×10^-3^ (7.94×10^-19^) | 12:110111515–111592381 | 0.08  (0.74) | 2.71×10^-4^  (8.15×10^-7^) | 1.86×10^-4^  (1.47×10^-3^) |  |
| T2D | 14:98987578–100039414 | 0.94  (4.09×10^-6^) | 1.45×10^-3^  (3.04×10^-6^) | 1.27×10^-3^  (4.85×10^-5^) | 14:98885323–99474533 | 0.17  (0.45) | 2.10×10^-4^  (2.51×10^-4^) | 3.14×10^-4^  (2.95×10^-6^) |  |
| T2D | 15:25803835–26656298 | 0.47  (1.10×10^-5^) | 3.02×10^-3^  (4.82×10^-19^) | 1.64×10^-3^  (1.55×10^-19^) | 15:25384328–26392947 | −0.22  (0.25) | 3.83×10^-4^  (1.56×10^-7^) | 3.10×10^-4^  (4.34×10^-5^) |  |
| T2D | 15:35034446–36592289 | 1.00  (1.70×10^-5^) | 8.29×10^-3^  (4.04×10^-3^) | 1.60×10^-3^  (2.50×10^-6^) | 15:35118928–36583327 | −0.02  (0.91) | 4.42×10^-4^  (3.42×10^-10^) | 4.98×10^-4^  (2.48×10^-9^) |  |
| **T2D** | **18:30265237**–**32456385** | 1.00  (1.65×10^-6^) | 1.54×10^-3^ (9.14×10^-7^) | 4.26×10^-3^  (8.03×10^-3^) | **18:30401129**–**32458907** | **0.56**  **(0.01)** | 4.68×10^-4^  (1.18×10^-10^) | 1.79×10^-4^  (5.38×10^-3^) |  |
| **T2D** | **18:57130984**–**58092858** | 0.56  (4.00×10^-5^) | 2.77×10^-3^  (6.20×10^-16^) | 1.59×10^-3^  (3.19×10^-9^) | **18:57635822**–**58527236** | **0.87**  **(2.37×10^-18^)** | 2.24×10^-3^  (5.54×10^-121^) | 4.51×10^-4^ (6.44×10^-11^) |  |
| T2D | 20:1769210–2663437 | 0.49  (3.52×10^-5^) | 2.32×10^-3^  (1.98×10^-12^) | 2.43×10^-3^  (1.53×10^-19^) | 20:1771286–2668108 | 0.01  (0.98) | 4.04×10^-4^  (4.47×10^-9^) | (1.28×10^-4^) (0.04) |  |
| CVD | 1:169848437–170916644 | 0.87  (1.20×10^-5^) | 1.02×10^-3^  (7.70×10^-5^) | 1.12×10^-3^  (8.22×10^-8^) | 1:171044256–172465153 | 0.22  (0.32) | 4.94×10^-4^  (4.81×10^-13^) | 1.70×10^-4^  (0.01) |  |
| **CVD** | **2:49913792**–**50821547** | 0.86  (2.29×10^-5^) | 9.50×10^-4^  (3.91×10^-4^) | 1.55×10^-3^  (2.80×10^-8^) | **2:49849186**–**50756240** | **0.53**  **(0.03)** | 2.54×10^-4^  (1.65×10^-5^) | 1.96×10^-4^ (1.38×10^-3^) |  |
| **CVD** | **5:52129899**–**53693420** | 1.00  (1.04×10^-5^) | 1.20×10^-3^  (9.95×10^-5^) | 4.06×10^-3^ (1.25×10^-5^) | **5:55221399**–**55968966** | **0.36**  **(0.01)** | 2.16×10^-4^  (3.28×10^-4^) | 3.31×10^-4^  (5.67×10^-5^) |  |
| CVD | 6:11228239–12372255 | 0.82  (1.46×10^-5^) | 1.43×10^-3^  (2.98×10^-6^) | 1.09×10^-3^  (1.18×10^-6^) | 6:10416551–11790671 | −0.28  (0.32) | 1.50×10^-4^  (0.01) | 3.18×10^-4^  (9.08×10^-5^) |  |
| CVD | 6:23197541–24101709 | −0.71  (3.62×10^-5^) | 8.45×10^-4^  (2.74×10^-3^) | 1.89×10^-3^  (4.19×10^-25^) | 6:23192338–23939306 | 0.20  (0.45) | 1.71×10^-4^  (2.48×10^-3^) | 2.38×10^-4^  (5.50×10^-4^) |  |
| CVD | 13:53609234–55588449 | 0.95  (2.65×10^-5^) | 1.56×10^-3^  (7.36×10^-7^) | 7.42×10^-4^  (2.06×10^-3^) | 13:54684857–55576912 | 0.17  (0.45) | 4.41×10^-4^  (5.17×10^-13^) | 1.37×10^-4^  (0.01) |  |
| LP | 3:2147501–3242009 | −0.51  (1.93×10^-6^) | 3.68×10^-3^  (5.95×10^-23^) | 2.80×10^-3^  (1.11×10^-15^) | 3:2140012–3128152 | −0.09  (0.72) | 1.69×10^-4^  (5.13×10^-3^) | 3.93×10^-4^  (1.76×10^-6^) |  |
| LP | 3:72012791–73244159 | 1.00  (3.73×10^-7^) | 1.60×10^-3^  (2.72×10^-6^) | 1.43×10^-3^  (1.79×10^-5^) | 3:72334705–73606612 | 0.16  (0.38) | 2.58×10^-4^  (1.19×10^-4^) | 5.87×10^-4^  (1.18×10^-10^) |  |
| **LP** | **6:105630354**–**106629704** | 0.96  (6.68×10^-9^) | 1.81×10^-3^  (6.28×10^-9^) | 1.77×10^-3^  (1.15×10^-7^) | **6:104951345**–**106053915** | **0.63**  **(0.05)** | 2.73×10^-4^  (1.35×10^-5^) | 1.16×10^-4^  (0.05) |  |
| **LP** | **7:101436195**–**103206379** | 1.00  (4.01×10^-5^) | 1.01×10^-3^  (3.02×10^-4^) | 9.72×10^-4^  (5.67×10^-4^) | **7:103191323**–**104109231** | **0.55**  **(0.05)** | 2.87×10^-4^  (1.11×10^-6^) | 1.30×10^-4^  (0.03) |  |
| LP | 15:73262649–74454643 | 0.91  (6.34×10^-7^) | 1.08×10^-3^  (1.08×10^-4^) | 2.21×10^-3^  (2.59×10^-11^) | 15:73375719–74458113 | 0.26  (0.14) | 7.50×10^-4^  (6.65×10^-24^) | 1.86×10^-4^  (4.01×10^-3^) | |
| FL | 1:202030681–203047059 | 0.61  (1.70×10^-5^) | 1.26×10^-3^  (1.85×10^-5^) | 3.38×10^-3^  (1.67×10^-26^) | **-** | **-** | **-** | **-** | |
| FL | 3:186602334–188069213 | 0.88  (6.16×10^-8^) | 2.00×10^-3^  (4.88×10^-9^) | 1.49×10^-3^  (2.70×10^-7^) | **-** | **-** | **-** | **-** | |
| FL | 6:2898448–4319834 | 0.73  (1.16×10^-10^) | 2.19×10^-3^  (4.61×10^-11^) | 4.27×10^-3^  (4.02×10^-30^) |  |  |  |  | |
| FL | 6:155739846–157043139 | −0.93  (1.99×10^-5^) | 7.72×10^-4^  (5.01×10^-3^) | 1.71×10^-3^  (1.38×10^-9^) |  |  |  |  | |
| FL | 12:52930959–53727562 | 1.00  (2.55×10^-5^) | 7.85×10^-4^  (1.42×10^-3^) | 1.22×10^-3^  (7.24×10^-5^) |  |  |  |  | |
| FL | 17:9751976–10575270 | −1.00  (5.03×10^-6^) | 2.01×10^-3^  (2.33×10^-10^) | 6.42×10^-4^  (7.30×10^-3^) |  |  |  |  | |

**B. Significant local genetic correlation between abdominal obesity and obesity-related diseases**

| **Disease** | **Discovery dataset** | | | | **Replication dataset of REP3_NHW_** | | | |
| --- | --- | --- | --- | --- | --- | --- | --- | --- |
|  | **Region** | **GC**  **(*P* value)** | **Disease** | **Region** | **GC**  **(*P* value)** | **Disease** | **Region** | **GC**  **(*P* value)** |
| **HT** | **1:32037899**–**33920739** | 0.79  (4.96×10^-5^) | 1.01  (9.00×10^-4^) | 2.47×10^-3^ (2.91×10^-11^) | **1:33066847**–**34371022** | **0.50**  **(8.00×10^-4^)** | 5.32×10^-4^  (5.26×10^-13^) | 3.93×10^-4^  (2.44×10^-8^) |
| HT | 2:1376568–2648919 | 0.46  (2.97×10^-5^) | 3.35×10^-3^ (4.16×10^-18^) | 4.00×10^-3^ (3.91×10^-19^) | 2:1875061–2680063 | 0.11  (0.57) | 3.63×10^-4^  (3.29×10^-8^) | 2.77×10^-4^  (1.09×10^-5^) |
| **HT** | **3:76161795**–**77157986** | 0.98  (2.22×10^-5^) | 1.57  (1.29×10^-6^) | 9.89×10^-4^ (1.87×10^-3^) | **3:76157471**–**77507832** | **0.56**  **(5.61×10^-3^)** | 4.02×10^-4^  (6.01×10^-9^) | 2.19×10^-4^  (5.13×10^-4^) |
| **HT** | **7:38970303**–**40921737** | 1.00  (1.57×10^-6^) | 6.32×10^-4^ (0.02) | 2.68×10^-3^ (9.30×10^-13^) | **7:40261241**–**41415925** | **0.54**  **(3.18×10^-5^)** | 5.13×10^-4^  (2.33×10^-13^) | 5.39×10^-4^  (3.08×10^-14^) |
| HT | 8:123448394–124868507 | 0.91  (5.06×10^-6^) | 1.74×10^-3^ (2.99×10^-7^) | 1.45×10^-3^ (1.16×10^-4^) | 8:124001020–125453322 | 0.21  (0.22) | 2.56×10^-4^  (1.27×10^-4^) | 5.87×10^-4^  (3.11×10^-14^) |
| HT | 10:133514844–134600671 | 1.00  (1.74×10^-6^) | 9.61×10^-4^ (1.96×10^-3^) | 1.64×10^-3^ (2.72×10^-5^) | 10:133818037–134856054 | −0.01  (0.95) | 2.67×10^-4^  (7.80×10^-5^) | 3.05×10^-4^  (1.14×10^-5^) |
| HT | 11:129394217–130424776 | 0.66  (3.83×10^-5^) | 1.90×10^-3^ (4.11×10^-9^) | 2.06×10^-3^ (7.29×10^-8^) | 11:129451785–130500783 | 0.11  (0.54) | 4.97×10^-4^  (1.09×10^-11^) | 2.34×10^-4^  (3.16×10^-4^) |
| HT | 12:110116509–112206126 | 0.66  (2.24×10^-7^) | 2.04×10^-3^ (1.68×10^-10^) | 3.42×10^-3^ (9.36×10^-20^) | 12:110111515–111592381 | 0.09  (0.65) | 1.90×10^-4^  (2.33×10^-4^) | 3.99×10^-4^  (2.59×10^-11^) |
| HT | 12:112206127–113925391 | 0.91  (5.03×10^-5^) | 9.77×10^-4^ (5.32×10^-4^) | 1.52×10^-3^ (3.35×10^-6^) | 12:111592382–113947983 | 0.12  (0.29) | 4.20×10^-4^  (2.15×10^-9^) | 1.07×10^-3^  (1.30×10^-36^) |
| HT | 15:80884682–82192960 | 0.75  (3.76×10^-5^) | 1.30×10^-3^ (5.91×10^-5^) | 2.30×10^-3^ (2.26×10^-9^) | 15:81077026–82191077 | −0.05  (0.75) | 3.19×10^-4^  (1.23×10^-6^) | 4.68×10^-4^  (3.34×10^-11^) |
| HT | 18:72211138–73568040 | 1.00  (3.91×10^-6^) | 1.10×10^-3^ (4.47×10^-4^) | 1.74×10^-3^  (2.05×10^-6^) | 18:72785207–73568260 | 0.02  (0.92) | 2.17×10^-4^  (3.30×10^-4^) | 4.80×10^-4^  (7.21×10^-12^) |
| HT | 19:40168770–41325021 | 1.00  (9.43×10^-6^) | 1.08×10^-3^ (8.51×10^-5^) | 1.01×10^-3^  (1.48×10^-4^) | 19:41004106–41733463 | −0.26  (0.31) | 1.62×10^-4^  (2.25×10^-3^) | 2.29×10^-4^  (6.15×10^-5^) |
| HT | 21:16262048–17832028 | 0.54  (8.32×10^-6^) | 2.90×10^-3^ (2.40×10^-15^) | 3.18×10^-3^  (2.54×10^-14^) | 21:16873444–18222037 | 0.17  (0.26) | 3.97×10^-4^  (7.75×10^-9^) | 4.50×10^-4^  (2.14×10^-10^) |
| T2D | 6:133866617–135139727 | −0.90 (4.11×10^-5^) | 1.14×10^-3^ (1.18×10^-4^) | 1.40×10^-3^  (3.10×10^-5^) | 6:134313644–135544083 | −0.11  (0.62) | 3.81×10^-4^  (4.79×10^-8^) | 2.02×10^-4^  (4.66×10^-3^) |
| T2D | 10:80219079–81191572 | 0.58  (1.42×10^-5^) | 2.58×10^-3^  (7.59×10^-13^) | 1.95×10^-3^  (3.15×10^-11^) | 10:79952997–81190573 | 0.07  (0.63) | 3.36×10^-4^  (4.74×10^-6^) | 7.86×10^-4^  (3.92×10^-16^) |
| T2D | 12:107850176–109020531 | 0.82  (8.98×10^-6^) | 1.99×10^-3^ (1.41×10^-9^) | 1.08×10^-3^  (6.62×10^-5^) | 12:108183189–109031819 | 0.08  (0.57) | 4.86×10^-4^  (1.96×10^-12^) | 4.43×10^-4^  (1.83×10^-9^) |
| T2D | 12:110116509–112206126 | 0.75  (6.81×10^-9^) | 2.04×10^-3^ (1.68×10^-10^) | 2.79×10^-3^ (7.94×10^-19^) | 12:110111515–111592381 | 0.41  (0.12) | 1.90×10^-4^  (2.33×10^-4^) | 1.86×10^-4^  (1.47×10^-3^) |
| T2D | 12:112206127–113925391 | 0.85  (1.93×10^-5^) | 9.77×10^-4^ (5.32×10^-4^) | 1.94×10^-4^  (6.02×10^-10^) | 12:111592382–113947983 | 0.20  (0.25) | 4.20×10^-4^  (2.15×10^-9^) | 3.32×10^-4^  (6.67×10^-6^) |
| T2D | 13:79597789–80800514 | 1.00  (1.65×10^-5^) | 1.32×10^-3^ (1.27×10^-5^) | 7.46×10^-4^  (4.34×10^-3^) | 13:79489046–80778339 | −0.40  (3.18×10^-3^) | 5.36×10^-4^  (1.77×10^-14^) | 4.93×10^-4^  (1.31×10^-10^) |
| T2D | 13:113803026–115109851 | 0.84  (7.37×10^-6^) | 1.41×10^-3^ (1.66×10^-5^) | 1.48×10^-3^  (4.99×10^-8^) | 13:113573119–114302561 | 0.02  (0.95) | 1.42×10^-4^  (8.72×10^-3^) | 4.76×10^-4^  (3.78×10^-10^) |
| **T2D** | **16:52662976**–**54022423** | 0.76  (1.05×10^-5^) | 1.74×10^-3^ (8.07×10^-8^) | 1.76×10^-3^  (4.01×10^-7^) | **16:52041337**–**53393882** | **0.52**  **(2.04×10^-5^)** | 5.88×10^-4^  (6.40×10^-15^) | 6.76×10^-4^  (7.90×10^-15^) |
| **T2D** | **17:1388659**–**2627122** | 0.83  (9.02×10^-6^) | 2.77×10^-3^ (1.68×10^-12^) | 8.66×10^-4^  (1.09×10^-3^) | **17:1480057**–**2699135** | **0.40**  **(6.52×10^-3^)** | 5.91×10^-4^  (2.04×10^-14^) | 4.03×10^-4^  (2.36×10^-7^) |
| **T2D** | **18:53755971**–**55101869** | 1.00  (1.71×10^-5^) | 1.71×10^-3^  (5.69×10^-7^) | 5.56×10^-4^  (0.01) | **18:53762997**–**54957867** | **0.43**  **(0.03)** | 2.61×10^-4^  (2.23×10^-5^) | 3.80×10^-4^  (1.15×10^-6^) |
| CVD | 2:100204144–101342115 | 1.00  (2.46×10^-5^) | 8.44×10^-4^  (1.03×10^-3^) | 7.94×10^-4^  (7.25×10^-4^) | 2:100202437–101556799 | 0.31  (0.10) | 5.71×10^-4^  (1.36×10^-15^) | 2.29×10^-4^  (1.88×10^-3^) |
| **CVD** | **7:80834729**–**82021734** | 1.00  (2.73×10^-6^) | 8.59×10^-4^  (4.43×10^-3^) | 1.24×10^-3^  (2.39×10^-8^) | **7:80055722**–**81533566** | **0.50**  **(0.02)** | 2.06×10^-4^  (1.40×10^-3^) | 3.96×10^-4^  (1.06×10^-6^) |
| CVD | 13:53609234–55588449 | 1.00  (2.43×10^-5^) | 9.74×10^-4^  (5.61×10^-4^) | 7.42×10^-4^  (2.06×10^-3^) | 13:54684857–55576912 | 0.35  (0.22) | 2.16×10^-4^  (5.58×10^-5^) | 1.37×10^-4^  (0.01) |
| CVD | 16:84987986–85890007 | −0.94  (3.09×10^-5^) | 4.61×10^-4^  (0.05) | 9.78×10^-4^  (9.87×10^-21^) | 16:85146806–86058597 | 0.11  (0.39) | 6.54×10^-4^  (7.48×10^-16^) | 5.60×10^-4^  (3.08×10^-9^) |
| **CVD** | **17:9751976**–**10575270** | 0.82  (3.95×10^-6^) | 3.14×10^-3^  (2.57×10^-18^) | 6.98×10^-4^  (1.04×10^-3^) | **17:9619357**–**10572617** | −**0.51**  **(0.02)** | 3.07×10^-4^  (4.67×10^-6^) | 2.81×10^-4^  (1.61×10^-4^) |
| HL | 1:234846609–236061177 | −1.00  (1.62×10^-5^) | 1.24×10^-3^  (4.58×10^-5^) | 7.13×10^-4^  (5.00×10^-3^) | 1:235097114–236199671 | 0.07  (0.78) | 1.86×10^-4^  (9.57×10^-4^) | 3.26×10^-4^  (2.57×10^-5^) |
| **HL** | **3:67856978**–**69346534** | 1.00  (4.61×10^-6^) | 1.05×10^-3^ (1.42×10^-3^) | 9.93×10^-4^  (6.72×10^-4^) | **3:68319771**–**69784969** | −**0.85**  **(6.73×10^-4^)** | 1.57×10^-4^  (8.78×10^-3^) | 3.58×10^-4^  (1.08×10^-6^) |
| HL | 6:14325796–15374604 | 0.82  (3.42×10^-5^) | 1.01×10^-3^  (4.84×10^-4^) | 2.06×10^-3^  (2.03×10^-9^) | 6:13352327–15069421 | 0.17  (0.34) | 3.95×10^-4^  (1.08×10^-7^) | 3.65×10^-4^  (1.03×10^-5^) |
| HL | 6:153085987–154461574 | −1.00  (2.52×10^-5^) | 8.25×10^-4^  (2.56×10^-3^) | 1.35×10^-3^  (3.48×10^-5^) | 6:154462011–155523957 | −0.20  (0.19) | 4.93×10^-4^  (3.81×10^-11^) | 4.06×10^-4^  (2.01×10^-7^) |
| HL | 11:114864027–116311324 | 0.68  (4.60×10^-6^) | 1.55×10^-3^  (2.75×10^-6^) | 2.66×10^-3^  (6.65×10^-16^) | 11:114742318–116247377 | −0.05  (0.79) | 4.53×10^-4^ (1.91×10^-9^) | 3.98×10^-4^  (4.00×10^-6^) |
| HL | 14:81807690–83342343 | 1.00  (3.38×10^-5^) | 1.81×10^-3^  (3.70×10^-7)^ | 4.55×10^-4^  (0.03) | 14:84087373–85562936 | 0.28  (0.24) | 2.79×10^-4^  (1.44×10^-5^) | 2.33×10^-4^  (1.30×10^-3^) |
| HL | 15:92881034–93723088 | 0.81  (9.01×10^-7^) | 1.79×10^-3^  (2.14×10^-7^) | 2.01×10^-3^  (8.05×10^-9^) | 15:92675406–93707010 | 0.08  (0.60) | 4.36×10^-4^  (2.63×10^-9^) | 5.18×10^-4^  (3.43×10^-9^) |
| HL | 15:95662426–96803466 | 1.00  (1.65×10^-5^) | 9.72×10^-4^  (1.40×10^-3^) | 1.09×10^-3^  (7.62×10^-6^) | 15:94565522–95662425 | 0.06  (0.78) | 5.47×10^-4^  (1.46×10^-12^) | 1.68×10^-4^  (0.02) |
| FL | 1:202030681–203047059 | 0.61  (1.70×10^-5^) | 1.26×10^-3^  (1.85×10^-5^) | 3.38×10^-3^  (1.67×10^-26^) |  |  |  |  |
| FL | 3:186602334–188069213 | 0.88  (6.16×10^-8^) | 2.00×10^-3^  (4.88×10^-9^) | 1.49×10^-3^  (2.70×10^-7^) |  |  |  |  |
| FL | 6:2898448–4319834 | 0.61  (1.56×10^-6^) | 1.66×10^-3^  (4.49×10^-7^) | 4.27×10^-3^  (4.02×10^-30^) | - | - | **-** | **-** |
| FL | 6:155739846–157043139 | −1.00  (3.83×10^-11^) | 2.15×10^-3^  (9.07×10^-9^) | 1.71×10^-3^  (1.38×10^-9^) | - | - | **-** | **-** |
| FL | 14:104620203–105279637 | −1.00  (3.84×10^-5^) | 6.49×10^-4^  (0.02) | 1.61×10^-3^  (1.91×10^-9^) | - | - | **-** | **-** |
| FL | 15:40554834–42332112 | 1.00  (2.16×10^-5^) | 6.81×10^-4^  (0.01) | 1.51×10^-3^  (1.09×10^-7^) | - | - | **-** | **-** |
