# Supplementary material for: Association of Multiple-trait Polygenic Risk Score with Obesity and Cardiometabolic Diseases in Korean Population
Source: Genomics Proteomics Bioinformatics. 2025 Nov 9;23(5):qzaf102. doi: 10.1093/gpbjnl/qzaf102 (PMC12975335; doi:10.1093/gpbjnl/qzaf102)
Supplement: qzaf102_Supplementary_Data [file qzaf102_supplementary_data.zip › 13-Feb-2026_113935_qzaf102_Supplementary_Data_TableSRevised.zip!/qzaf102_Supplementary_Data_TableSRevised/Table S14_Omitted.docx]

**Table S14 Validation results of each single trait PRS analyses**

(A) PRS validation results for SBP

| **Methods** | **Cor.** | ***P* value** | **AIC** |
| --- | --- | --- | --- |
| CT | 0.086 | 3.51×10^-26^ | 74058.11 |
| LDpred: infinitesimal | 0.112 | 8.40×10^-43^ | 73982.00 |
| LDpred: grid1 ($\rho$ = 1%) | 0.128 | 2.04×10^-56^ | 73919.52 |
| LDpred: grid2 ($\rho$ = 3%) | 0.128 | 4.69×10^-56^ | 73921.18 |
| LDpred: grid4 ($\rho$ = 10%) | 0.119 | 9.70×10^-49^ | 73954.76 |
| LDpred: grid3 ($\rho$ = 30%) | 0.115 | 4.40×10^-45^ | 73971.54 |
| LDpred: grid5 ($\rho$ = 100%) | 0.112 | 8.63×10^-43^ | 73982.05 |
| **LDpred: auto** | **0.132** | **1.31×10^-59^** | **73904.86** |
| lassosum | 0.129 | 1.04×10^-56^ | 73918.17 |
| PRS-CS | 0.113 | 5.83×10^-44^ | 73976.68 |

(B) PRS validation results for DBP

| **Methods** | **Cor.** | ***P* value** | **AIC** |
| --- | --- | --- | --- |
| CT | 0.074 | 8.87×10^-20^ | 62949.91 |
| LDpred: infinitesimal | 0.107 | 5.15×10^-39^ | 62861.94 |
| LDpred: grid1 ($\rho$ = 1%) | 0.123 | 7.49×10^-51^ | 62807.64 |
| LDpred: grid2 ($\rho$ = 3%) | 0.119 | 3.31×10^-48^ | 62819.78 |
| LDpred: grid3 ($\rho$ = 10%) | 0.111 | 3.16×10^-42^ | 62847.21 |
| LDpred: grid4 ($\rho$ = 30%) | 0.108 | 1.81×10^-39^ | 62859.86 |
| LDpred: grid5 ($\rho$ = 100%) | 0.107 | 4.58×10^-39^ | 62861.70 |
| **LDpred: auto** | **0.125** | **7.27×10^-53^** | **62798.40** |
| lassosum | 0.114 | 3.75×10^-44^ | 62838.38 |
| PRS-CS | 0.104 | 1.97×10^-36^ | 62873.78 |

(C) PRS validation results for BS

| **Methods** | **Cor.** | ***P* value** | **AIC** |
| --- | --- | --- | --- |
| CT | 0.105 | 6.53×10^-37^ | 79360.22 |
| LDpred: infinitesimal | 0.055 | 2.53×10^-13^ | 79467.89 |
| LDpred: grid1 ($\rho$ = 1%) | 0.103 | 2.93×10^-38^ | 79354.05 |
| LDpred: grid2 ($\rho$ = 3%) | 0.085 | 2.06×10^-27^ | 79403.70 |
| LDpred: grid3 ($\rho$ = 10%) | 0.065 | 2.00×10^-17^ | 79449.26 |
| LDpred: grid4 ($\rho$ = 30%) | 0.058 | 2.43×10^-14^ | 79463.28 |
| LDpred: grid5 ($\rho$ = 100%) | 0.055 | 2.46×10^-13^ | 79467.83 |
| LDpred: auto | 0.112 | 2.20×10^-44^ | 79325.97 |
| **lassosum** | **0.124** | **2.81×10^-51^** | **79294.33** |
| PRS-CS | 0.061 | 2.36×10^-15^ | 79458.68 |

(D) PRS validation results for TG

| **Methods** | **Cor.** | ***P* value** | **AIC** |
| --- | --- | --- | --- |
| CT | 0.184 | 4.07×10^-109^ | 122473.10 |
| LDpred: infinitesimal | 0.131 | 7.46×10^-58^ | 122708.70 |
| LDpred: grid1 ($\rho$ = 1%) | 0.238 | 3.84×10^-186^ | 122118.50 |
| LDpred: grid2 ($\rho$ = 3%) | 0.226 | 2.30×10^-168^ | 122200.40 |
| LDpred: grid3 ($\rho$ = 10%) | 0.199 | 1.81×10^-130^ | 122374.80 |
| LDpred: grid4 ($\rho$ = 30%) | 0.166 | 6.28×10^-92^ | 122552.10 |
| LDpred: grid5 ($\rho$ = 100%) | 0.130 | 4.57×10^-57^ | 122712.40 |
| LDpred: auto | 0.239 | 1.01×10^-188^ | 122106.70 |
| **lassosum** | **0.246** | **6.07×10^-199^** | **122059.60** |
| PRS-CS | 0.168 | 1.44×10^-92^ | 122549.20 |

(E) PRS validation results for HDL

| **Methods** | **Cor.** | ***P* value** | **AIC** |
| --- | --- | --- | --- |
| CT | 0.255 | 2.57×10^-221^ | 69083.89 |
| LDpred: infinitesimal | 0.163 | 3.98×10^-96^ | 69660.17 |
| LDpred: grid1 ($\rho$ = 1%) | 0.301 | 2.77×10^-322^ | 68618.75 |
| LDpred: grid2 ($\rho$ = 3%) | 0.287 | 2.12×10^-292^ | 68756.47 |
| LDpred: grid3 ($\rho$ = 10%) | 0.242 | 1.23×10^-208^ | 69142.28 |
| LDpred: grid4 ($\rho$ = 30%) | 0.195 | 1.01×10^-135^ | 69477.98 |
| LDpred: grid5 ($\rho$ = 100%) | 0.163 | 1.06×10^-96^ | 69657.53 |
| LDpred: auto | 0.278 | 5.22×10^-296^ | 68864.22 |
| **lassosum** | **0.312** | **0** | **68545.46** |
| PRS-CS | 0.220 | 2.07×10^-171^ | 69313.70 |

*Note:* CT, clumping and thresholding; *P* value, P value for PRS association; AIC, Akaike Information Criterion from linear models for BMI, adjusted for age, sex, PRS, and the first five principal components.
