# Supplementary material for: Association of Multiple-trait Polygenic Risk Score with Obesity and Cardiometabolic Diseases in Korean Population
Source: Genomics Proteomics Bioinformatics. 2025 Nov 9;23(5):qzaf102. doi: 10.1093/gpbjnl/qzaf102 (PMC12975335; doi:10.1093/gpbjnl/qzaf102)
Supplement: qzaf102_Supplementary_Data [file qzaf102_supplementary_data.zip › 13-Feb-2026_113935_qzaf102_Supplementary_Data_TableSRevised.zip!/qzaf102_Supplementary_Data_TableSRevised/Table S15_Omitted.docx]

**Table S15** **Model performance improvement using single trait and multiple trait PRS analyses**

| **Trait** | **AIC** | | | | | **AUC** | | | | | | |  |
| --- | --- | --- | --- | --- | --- | --- | --- | --- | --- | --- | --- | --- | --- |
|  | **M1** | **M2** | **M3** | **M4** | **M1** | | **M2** | ***P* value: M1vs.M2** | **M3** | **P: M1/2vs.M3** | **M4** | ***P* value: M3vs.M4** | |
| BMI25 | 85801.97 | 86697.21 | 85454.59 | 85453.69 | 0.628 | | 0.611 | **1.64×10^-18^** | 0.635 | **1.22×10^-59^** | 0.635 | 0.13 | |
| BMI30 | 18106.06 | 18346.71 | 17995.36 | 17998.04 | 0.645 | | 0.610 | **7.76×10^-7^** | 0.659 | **5.42×10^-23^** | 0.659 | 0.53 | |
| WC1 | 72070.99 | 71991.22 | 71604.23 | 71603.43 | 0.623 | | 0.626 | 0.23 | 0.634 | **1.03×10^-17^** | 0.635 | 0.08 | |
| WC2 | 20031.98 | 20081.08 | 19893.26 | 19896.41 | 0.638 | | 0.631 | 0.19 | 0.653 | **2.50×10^-12^** | 0.654 | 0.18 | |
| HT | 59319.81 | 59319.34 | 59284.60 | 58473.65 | 0.698 | | 0.698 | 0.77 | 0.698 | **0.02** | 0.712 | **1.36×10^-44^** | |
| T2D | 33204.80 | 33136.06 | 33138.05 | 32831.37 | 0.679 | | 0.682 | **2.40×10^-4^** | 0.682 | 0.92 | 0.697 | **8.31×10^-22^** | |
| CVD | 20426.18 | 20419.98 | 20420.34 | 20416.28 | 0.742 | | 0.742 | 0.49 | 0.742 | 0.41 | 0.743 | 0.06 | |
| HL | 42038.24 | 42034.94 | 42036.52 | 41814.87 | 0.592 | | 0.592 | 0.65 | 0.592 | 0.78 | 0.606 | **7.11×10^-11^** | |
| FL | 21239.98 | 21229.98 | 21228.91 | 21216.84 | 0.653 | | 0.653 | 0.69 | 0.654 | 0.55 | 0.657 | **0.03** | |

*Note: P* value, DeLong test P value for comparing AUC between models. Model descriptions — M1, adjusted for age, sex, BMI PRS, and the first five principle components; M2, adjusted for age, sex, WC PRS, and PC1–5; M3, adjusted for age, sex, BMI PRS, WC PRS, and PC1–5; M4, adjusted for age, sex, PRSs for BMI, WC, SBP, DBP, BS, TG, HDL, and PC1–5.
