# Supplementary material for: Association of Multiple-trait Polygenic Risk Score with Obesity and Cardiometabolic Diseases in Korean Population
Source: Genomics Proteomics Bioinformatics. 2025 Nov 9;23(5):qzaf102. doi: 10.1093/gpbjnl/qzaf102 (PMC12975335; doi:10.1093/gpbjnl/qzaf102)
Supplement: qzaf102_Supplementary_Data [file qzaf102_supplementary_data.zip › 13-Feb-2026_113935_qzaf102_Supplementary_Data_TableSRevised.zip!/qzaf102_Supplementary_Data_TableSRevised/Table S16_Omitted.docx]

**Table S16 Validation results for obesity-related diseases: T2D and CVD**

| **Methods** | **T2D** | | | **CVD** | | |
| --- | --- | --- | --- | --- | --- | --- |
|  | ***P* value** | **AIC** | **AUC** | ***P* value** | **AIC** | **AUC** |
| CT | 2.01×10^-38^ | 6454.28 | 0.716 | 1.86×10^-3^ | 4000.05 | 0.746 |
| LDpred: infinitesimal | 5.68×10^-36^ | 6462.21 | 0.716 | 1.99×10^-6^ | 3987.01 | 0.747 |
| LDpred: grid1 ($\rho$ = 1%) | 1.69×10^-57^ | 6355.18 | 0.734 | 3.93×10^-7^ | 3983.89 | 0.750 |
| LDpred: grid2 ($\rho$ = 3%) | 3.50×10^-55^ | 6366.96 | 0.732 | **2.56×10^-7^** | **3983.04** | **0.749** |
| LDpred: grid4 ($\rho$ = 10%) | 5.67×10^-48^ | 6403.10 | 0.726 | 5.37×10^-7^ | 3984.45 | 0.748 |
| LDpred: grid3 ($\rho$ = 30%) | 1.00×10^-40^ | 6439.01 | 0.719 | 1.82×10^-6^ | 3986.82 | 0.747 |
| LDpred: grid5 ($\rho$ = 100%) | 6.04×10^-36^ | 6462.34 | 0.715 | 2.29×10^-6^ | 3987.28 | 0.747 |
| **LDpred: auto** | **1.05×10^-57^** | **6354.16** | **0.734** | 2.37×10^-6^ | 3987.38 | 0.749 |
| lassosum | 6.73×10^-30^ | 6491.62 | 0.709 | 4.27×10^-7^ | 3983.99 | 0.749 |
| PRS-CS | 5.07×10^-46^ | 6413.33 | 0.724 | 4.43×10^-6^ | 3988.55 | 0.747 |
