# Supplementary material for: Association of Multiple-trait Polygenic Risk Score with Obesity and Cardiometabolic Diseases in Korean Population
Source: Genomics Proteomics Bioinformatics. 2025 Nov 9;23(5):qzaf102. doi: 10.1093/gpbjnl/qzaf102 (PMC12975335; doi:10.1093/gpbjnl/qzaf102)
Supplement: qzaf102_Supplementary_Data [file qzaf102_supplementary_data.zip › 13-Feb-2026_113935_qzaf102_Supplementary_Data_TableSRevised.zip!/qzaf102_Supplementary_Data_TableSRevised/Table S17_Omitted.docx]

**Table S17 Interaction and mediation effects of obesity on obesity-related diseases: T2D and CVD**

(A) Interaction effect of obesity on obesity-related diseases

| **Disease PRS** | **Obesity PRS** | **Obesity** | **Interaction**  **with disease**  **PRS** | **Disease PRS** | | **Obesity PRS** | | **Obesity** | | **Interaction** | |
| --- | --- | --- | --- | --- | --- | --- | --- | --- | --- | --- | --- |
|  |  |  |  | **OR (CI)** | ***P* value** | **OR (CI)** | ***P* value** | **OR (CI)** | ***P* value** | **OR (CI)** | ***P* value** |
| T2D | BMI | BMI25 | BMI PRS | 1.57 (1.52,1.62) | **3.12×10^-174^** | 0.92 (0.89,0.96) | **2.39×10^-6^** | 2.04 (1.92,2.17) | **5.63×10^-114^** | 1.00 (0.97,1.04) | 0.79 |
| T2D | BMI | BMI25 | BMI25 | 1.67 (1.60,1.74) | **4.58×10^-116^** | 0.93 (0.90,0.96) | **1.21×10^-6^** | 2.12 (1.99,2.26) | **7.88×10^-115^** | 0.88 (0.83,0.94) | **9.90×10^-5^** |
| T2D | WC | WC1 | WC PRS | 1.56 (1.51,1.61) | **5.27×10^-162^** | 1.00 (0.97,1.03) | 0.93 | 2.21 (2.07,2.35) | **1.32×10^-135^** | 1.00 (0.97,1.03) | 0.95 |
| T2D | WC | WC1 | WC1 | 1.63 (1.57,1.70) | **9.83×10^-120^** | 1.00 (0.97,0.13) | 0.93 | 2.29 (2.15,2.45) | **6.29×10^-135^** | 0.89 (0.83,0.95) | **2.22×10^-4^** |
| T2D | PRSsum1 | BMI25 | PRSsum1 | 1.56 (1.52,1.62) | **1.59×10^-167^** | 0.96 (0.93,0.99) | **0.02** | 2.01 (1.89,2.14) | **4.24×10^-110^** | 1.00 (0.97,1.03) | 0.93 |
| T2D | PRSsum1 | BMI25 | BMI25 | 1.66 (1.59,1.74) | **1.16×10^-113^** | 0.96 (0.93,0.99) | **0.01** | 2.10 (1.96,2.24) | **3.56×10^-111^** | 0.88 (0.83,0.94) | **9.31×10^-5^** |
| T2D | PRSsum2 | WC1 | PRSsum2 | 1.56 (1.51,1.61) | **3.85×10^-165^** | 0.98 (0.95,1.02) | 0.33 | 2.21 (2.08,2.36) | **6.09×10^-137^** | 1.00 (0.97,1.03) | 0.80 |
| T2D | PRSsum2 | WC1 | WC1 | 1.64 (1.57,1.71) | **2.82×10^-121^** | 0.98 (0.95,1.01) | 0.26 | 2.30 (2.16,2.46) | **3.73×10^-136^** | 0.89 (0.83,0.95) | **2.20×10^-4^** |
| CVD | BMI | BMI25 | BMI PRS | 1.23 (1.18,1.28) | **1.30×10^-24^** | 1.01 (0.97,1.05) | 0.78 | 1.45 (1.34,1.57) | **1.22×10^-19^** | 1.02 (0.98,1.06) | 0.33 |
| CVD | BMI | BMI25 | BMI25 | 1.24 (1.17,1.30) | **3.71×10^-15^** | 1.01 (0.97,1.05) | 0.66 | 1.45 (1.34,1.58) | **2.42×10^-19^** | 0.99 (0.91,1.07) | 0.79 |
| CVD | WC | WC1 | WC PRS | 1.24 (1.19,1.29) | **1.70×10^-24^** | 1.03 (0.99,1.07) | 0.16 | 1.51 (1.38,1.64) | **1.15×10^-21^** | 1.02 (0.98,1.06) | 0.29 |
| CVD | WC | WC1 | WC1 | 1.25 (1.19,1.32) | **6.47×10^-18^** | 1.03 (0.99,1.08) | 0.10 | 1.52 (1.39,1.65) | **1.11×10^-21^** | 0.97 (0.89,1.05) | 0.44 |
| CVD | PRSsum1 | BMI25 | PRSsum1 | 1.23 (1.18,1.28) | **3.69×10^-24^** | 1.02 (0.98,1.06) | 0.28 | 1.44 (1.33,1.56) | **5.68×10^-19^** | 1.03 (0.99,1.07) | 0.14 |
| CVD | PRSsum1 | BMI25 | BMI25 | 1.24 (1.17,1.30) | **5.01×10^-15^** | 1.03 (0.99,1.07) | 0.18 | 1.44 (1.33,1.57) | **1.09×10^-18^** | 0.99 (0.91,1.07) | 0.79 |
| CVD | PRSsum2 | WC1 | PRSsum2 | 1.23 (1.18,1.28) | **7.51×10^-24^** | 1.05 (1.01,1.10) | **0.01** | 1.50 (1.38,1.63) | **3.58×10^-21^** | 1.01 (0.98,1.06) | 0.46 |
| CVD | PRSsum2 | WC1 | WC1 | 1.25 (1.19,1.31) | **1.47×10^-17^** | 1.06 (1.01,1.10) | **7.81×10^-3^** | 1.51 (1.38,1.64) | **3.31×10^-21^** | 0.97 (0.89,1.05) | 0.43 |

*Note:* Model, Disease ~ age + sex + PC1-5 + obesity status + obesity PRSs (BMI PRS, WC PRS, PRSsum1, PRSsum2) + target disease PRS + obesity status/obesity PRS × disease PRS.

(B) Mediation effects of obesity on the associations between obesity PRS and obesity-related diseases

| **Disease (Y)** | **Obesity PRS (X)** | **Obesity (M)** | **Disease PRS**  **adjustment** | **Direct effect** | | **Indirect effect** | | **Prop. of med. effect** | |
| --- | --- | --- | --- | --- | --- | --- | --- | --- | --- |
|  |  |  |  | **Beta** | ***P* value** | **Beta** | ***P* value** | **Beta** | ***P* value** |
| T2D | BMI PRS | BMI25 | unadjusted | -0.00084 | 0.470 | 0.00431 | **< 2×10^-16^** | 1.24214 | < 2×10^-16^ |
| T2D | BMI PRS | BMI25 | adjusted | -0.00511 | **< 2×10^-16^** | 0.00431 | **< 2×10^-16^** | 5.34000 | 0.370 |
| T2D | WC PRS | WC1 | unadjusted | 0.00579 | **< 2×10^-16^** | 0.00374 | **< 2×10^-16^** | 0.39241 | **< 2×10^-16^** |
| T2D | WC PRS | WC1 | adjusted | -0.00012 | 0.900 | 0.00326 | **< 2×10^-16^** | 1.03800 | 0.008 |
| T2D | PRSsum1 | BMI25 | unadjusted | 0.00382 | **< 2×10^-16^** | 0.00464 | **< 2×10^-16^** | 0.54846 | **< 2×10^-16^** |
| T2D | PRSsum1 | BMI25 | adjusted | -0.00271 | **0.020** | 0.00477 | **< 2×10^-16^** | 2.32000 | 0.106 |
| T2D | PRSsum1 | WC1 | unadjusted | 0.00365 | **< 2×10^-16^** | 0.00423 | **< 2×10^-16^** | 0.53627 | **< 2×10^-16^** |
| T2D | PRSsum1 | WC1 | adjusted | -0.00272 | **0.018** | 0.00391 | **< 2×10^-16^** | 3.28000 | 0.252 |
| T2D | PRSsum2 | BMI25 | unadjusted | 0.00459 | **< 2×10^-16^** | 0.00399 | **< 2×10^-16^** | 0.46483 | **< 2×10^-16^** |
| T2D | PRSsum2 | BMI25 | adjusted | -0.00101 | 0.358 | 0.00379 | **< 2×10^-16^** | 1.36073 | 0.024 |
| T2D | PRSsum2 | WC1 | unadjusted | 0.00429 | **< 2×10^-16^** | 0.00362 | **< 2×10^-16^** | 0.45733 | **< 2×10^-16^** |
| T2D | PRSsum2 | WC1 | adjusted | -0.00117 | 0.244 | 0.00320 | **< 2×10^-16^** | 1.57563 | 0.074 |
| CVD | BMI PRS | BMI25 | unadjusted | 0.00064 | 0.474 | 0.00132 | **< 2×10^-16^** | 0.67360 | **0.018** |
| CVD | BMI PRS | BMI25 | adjusted | 0.00039 | 0.634 | 0.00139 | **< 2×10^-16^** | 0.77956 | **0.032** |
| CVD | WC PRS | WC1 | unadjusted | 0.00184 | **0.042** | 0.00103 | **< 2×10^-16^** | 0.35800 | **< 2×10^-16^** |
| CVD | WC PRS | WC1 | adjusted | 0.00140 | 0.112 | 0.00095 | **< 2×10^-16^** | 0.40395 | **0.002** |
| CVD | PRSsum1 | BMI25 | unadjusted | 0.00161 | 0.064 | 0.00143 | **< 2×10^-16^** | 0.47000 | **< 2×10^-16^** |
| CVD | PRSsum1 | BMI25 | adjusted | 0.00118 | 0.190 | 0.00153 | **< 2×10^-16^** | 0.56403 | **< 2×10^-16^** |
| CVD | PRSsum1 | WC1 | unadjusted | 0.00186 | **0.038** | 0.00126 | **< 2×10^-16^** | 0.40432 | **< 2×10^-16^** |
| CVD | PRSsum1 | WC1 | adjusted | 0.00141 | 0.096 | 0.00123 | **< 2×10^-16^** | 0.46552 | **0.006** |
| CVD | PRSsum2 | BMI25 | unadjusted | 0.00297 | **0.002** | 0.00119 | **< 2×10^-16^** | 0.28543 | **< 2×10^-16^** |
| CVD | PRSsum2 | BMI25 | adjusted | 0.00230 | **0.006** | 0.00121 | **< 2×10^-16^** | 0.34491 | **< 2×10^-16^** |
| CVD | PRSsum2 | WC1 | unadjusted | 0.00295 | **< 2×10^-16^** | 0.00100 | **< 2×10^-16^** | 0.25246 | **< 2×10^-16^** |
| CVD | PRSsum2 | WC1 | adjusted | 0.00227 | **0.002** | 0.00087 | **< 2×10^-16^** | 0.27610 | **< 2×10^-16^** |

*Note:* Y, dependent variable (obesity-related diseases); X, independent variable (obesity PRS); M, mediator (obesity); Prop. of med. effect, Proportion of mediation effect; Model, unadjusted, without adjustment of diseases PRS, adjusted, with adjustment of disease PRS in first/third sequential models.
